# Supplementary material for: Molecular Tetris by sequence-specific stacking of hydrogen bonding molecular clips
Source: Commun Chem. 2022 Dec 28;5:180. doi: 10.1038/s42004-022-00802-4 (PMC9814962; doi:10.1038/s42004-022-00802-4)
Supplement: Supplementary file 2 — Supplementary Information [file 42004_2022_802_MOESM2_ESM.pdf]

Supplementary Information

Molecular Tetris by sequence-specific stacking of hydrogen bonding molecular clips

Hyun Lee and Dongwhan Lee*

Department of Chemistry, Seoul National University, 1 Gwanak-ro, Gwanak-gu, Seoul 08826, Korea

Supplementary Methods

General Considerations. Unless otherwise noted, all reagents were purchased from commercial suppliers and used as received. The compounds 4,7-dibromobenzo[*c*]-1,2,5-thiadiazole[1], 2,1,3-benzothiadiazole-4,7-bis(boronic acid pinacol ester)[2], 2-bromo-5-(2-methoxyethoxy)pyridine[3], 8-bromo-2,3,4,9-tetrahydro-1*H*-carbazol-1-one[4,5], pyrene-2-boronic acid pinacol ester[6], and pyrene-4-boronic acid pinacol ester[7] were prepared according to literature procedures.

Supplementary Scheme 1. Synthetic route to **C-NI**, **C-P1**, **C-P2**, and **C-P4**.

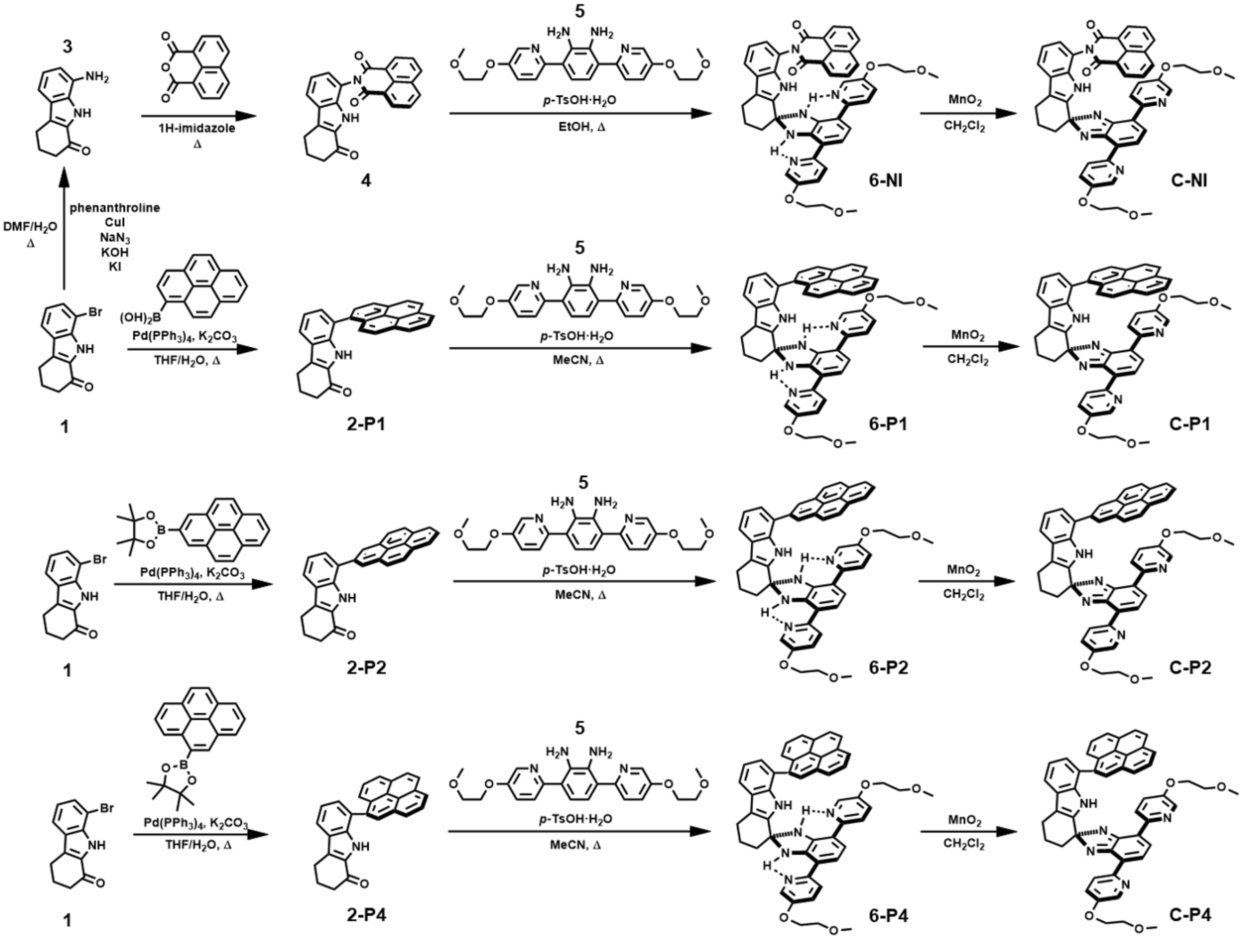

2-(4,7-Bis(5-(2-methoxyethoxy)pyridin-2-yl)-2',3',4',9'-tetrahydrospiro[benzo[*d*] imidazole-2,1'-carbazol]-8'-yl)-1*H*-benzo[*de*]isoquinoline-1,3(2*H*)-dione (C-NI). A 50 mL round-bottom flask was loaded with **6-NI** (0.0764 g, 98.9 μmol), MnO_2 (0.0912 g, 1.05 mmol), and CH_2Cl_2 (50 mL). The mixture was stirred at r.t. for 10 min, filtered through Celite, and concentrated under reduced pressure. The residual material was taken up in CH_2Cl_2 (100 mL), washed with water (100 mL × 3), dried over anhyd $MgSO_4$, filtered, and concentrated under reduced pressure. Flash column chromatography on SiO_2 (CH_2Cl_2:EtOAc = 100:1 to 1:2, v/v) furnished **C-NI** as a red solid (0.0670 g, 86.9 μmol, yield = 88%). ^{1}H NMR (400 MHz, $CDCl_3$, 9.6 mM, 298 K): δ 11.07 (s, 1H), 8.25 (d, J = 8.5 Hz, 2H), 7.95 (d, J = 7.0 Hz, 2H), 7.75–7.73 (m, 3H),

7.65 (s, 2H), 7.24–7.20 (m, 1H), 7.17 (t, J = 7.5 Hz, 2H), 7.06 (s, 2H), 7.01 (d, J = 7.4 Hz, 1H), 6.72–6.63 (m, 2H), 3.85–3.76 (m, 2H), 3.62–3.52 (m, 6H), 3.43 (s, 6H), 3.19 (t, J = 5.8 Hz, 2H), 2.55–2.47 (m, 2H), 2.27–2.18 (m, 2H). ^{13}C NMR (125 MHz, $CDCl_3$, 9.2 mM, 298 K): δ 164.11, 160.20, 153.99, 144.20, 136.30, 134.08, 133.37, 132.16, 131.15, 130.87, 130.41, 129.99, 127.68, 127.02, 126.01, 125.04, 122.40, 122.31, 120.81, 119.63, 119.38, 119.07, 115.09, 104.56, 70.78, 67.40, 59.41, 29.85, 23.27, 21.94. FT-IR (ATR, cm^{-1}): 2924, 2853, 1735, 1706, 1661, 1587, 1499, 1372, 1262, 1235, 1201, 1124, 1095, 1058, 1032, 925, 832. HRMS (ESI) calcd for $C_{46}H_{39}N_6O_6$ $[M + H]^+$ 771.2926, found 771.2928.

4,7-Bis(5-(2-methoxyethoxy)pyridin-2-yl)-8'-(pyren-1-yl)-2',3',4',9'-tetrahydrospiro[benzo[*d*]imidazole-2,1'-carbazole] (C-P1). A 50 mL round-bottom flask was loaded with **6-P1** (62.7 mg, 80.6 μmol), MnO_2 (69.1 mg, 0.795 mmol) and CH_2Cl_2 (10 mL). The mixture was stirred at r.t. for 10 min, filtered through Celite, and concentrated under reduced pressure. The residual material was taken up in CH_2Cl_2 (100 mL), washed with water (100 mL $\times$ 3), dried over anhyd $MgSO_4$, filtered, and concentrated under reduced pressure. Flash column chromatography on SiO_2 (CH_2Cl_2:EtOAc = 100:1 to 1:2 $\rightarrow$ CH_2Cl_2:EtOAc:MeOH = 45:50:5 v/v) furnished **C-P1** as a red solid (54.1 mg, 69.7 μmol, yield = 87%). ^{1}H NMR (400 MHz, $CDCl_3$, 43.0 mM, 298 K): δ 9.85 (br, 1H), 8.23 (d, J = 8.7 Hz, 1H), 8.01 (d, J = 8.4 Hz, 1H), 7.81–7.65 (m, 2H), 7.62 (d, J = 7.5 Hz, 1H), 7.56 (d, J = 8.9 Hz, 1H), 7.51–7.42 (m, 5H), 7.40 (d, J = 7.4 Hz, 1H), 7.34 (d, J = 8.8 Hz, 1H), 7.29–7.23 (t, J = 9.9 Hz, 1H), 7.17 (d, J = 6.9 Hz, 1H), 7.11 (br, 1H), 6.80–6.69 (br, 1H), 6.56–6.45 (br, 1H), 6.44–6.30 (br, 1H), 6.25–6.09 (br, 1H), 3.73–3.61 (br, 2H), 3.57–3.47 (br, 2H), 3.46–3.28 (m, 10H), 3.28–3.12 (br, 2H), 2.60–2.45 (br, 2H), 2.25–2.06 (m, J = 29.9, 5.5 Hz, 2H). ^{13}C NMR (100 MHz, $CDCl_3$, 24.7 mM, 298 K): δ 160.14, 159.95, 153.56, 153.30, 144.01, 143.55, 137.25, 137.09, 136.99, 134.35, 131.49, 131.27, 130.78, 130.67, 130.08, 129.89, 129.59, 129.51, 127.75, 127.51, 127.48, 127.42, 126.83, 126.46, 125.48, 125.21, 124.73, 124.67, 124.63, 124.29, 124.25, 124.14, 124.10, 123.28, 119.73, 119.27, 118.70, 118.11, 114.21, 104.23, 70.79, 70.69, 67.27, 66.94, 59.36, 59.34, 32.21, 23.41, 21.94. FT-IR (ATR, cm^{-1}): 3047, 2925, 1586, 1551, 1467, 1411, 1291, 1227, 1267, 1122, 1033, 1010, 922, 848. HRMS (ESI) calcd for $C_{50}H_{42}N_5O_4$ $[M + H]^+$ 776.3231, found 776.3234.

4,7-Bis(5-(2-methoxyethoxy)pyridin-2-yl)-8'-(pyren-2-yl)-2',3',4',9'-tetrahydrospiro[benzo[*d*]imidazole-2,1'-carbazole] (C-P2). To a CH_2Cl_2 solution (10 mL) of **6-P2** (23.3 mg, 30.0 μmol), MnO_2 (51.0 mg, 0.587 mmol) was loaded. The mixture was stirred at r.t. for 10 min, filtered through Celite, and concentrated under reduced pressure. The residual material was taken up in CH_2Cl_2 (100 mL), washed with water (100 mL $\times$ 3), dried over anhyd $MgSO_4$, filtered, and concentrated under reduced pressure. Flash column chromatography on SiO_2 (CH_2Cl_2:EtOAc = 100:1 to 1:2 $\rightarrow$ CH_2Cl_2:EtOAc:MeOH = 45:50:5, v/v) furnished **C-P2** as a red solid (20.7 mg, 26.7 μmol, yield = 89%). ^{1}H NMR (500 MHz, $CDCl_3$, 13.3 mM, 298 K): δ 9.51 (s, 1H), 8.21 (d, J = 8.7 Hz, 2H), 7.90 (s, 2H), 7.87 (d, J = 7.5 Hz, 2H), 7.80 (dd, J = 8.3, 6.7 Hz, 1H), 7.70 (dd, J = 7.3, 1.7 Hz, 1H), 7.67 (s, 2H), 7.59 (d, J = 8.8 Hz, 2H), 7.46 (d, J = 8.8 Hz, 2H), 7.25–7.21 (m, 2H), 6.95 (s, 2H), 6.67 (d, J = 7.0 Hz, 2H), 3.84–3.70 (m, 4H), 3.60 (dd, J = 9.3, 4.5 Hz, 4H), 3.43 (s, 6H), 3.21 (t, J = 6.1 Hz, 2H), 2.60–2.53 (m, 2H), 2.20–2.12 (m, 2H). ^{13}C NMR (125 MHz, $CDCl_3$, 20.0 mM, 298 K): δ 160.50, 153.94, 144.20, 137.34, 137.17, 136.28, 131.84, 131.10, 130.79, 130.71, 127.97, 127.28, 127.17, 127.06, 126.66, 125.58, 125.50, 125.13, 124.65, 124.14, 123.44, 122.84, 119.69, 119.10, 118.03, 114.55, 104.54, 70.80, 67.32, 59.41, 32.92, 23.37, 21.86. FT-IR (ATR, cm^{-1}): 3052, 2925, 2843, 1587, 1555, 1484, 1394, 1368, 1290, 1260, 1219, 1198, 1125, 1058, 1030, 975, 924, 880, 841. HRMS (ESI) calcd for $C_{50}H_{42}N_5O_4$ $[M + H]^+$ 776.3231, found 776.3236.

4,7-Bis(5-(2-methoxyethoxy)pyridin-2-yl)-8'-(pyren-4-yl)-2',3',4',9'-

tetrahydrospiro[benzo[*d*]imidazole-2,1'-carbazole] (C-P4). To a CH_2Cl_2 solution (10 mL) of **6-P4** (0.0779 g, 0.100 mmol), MnO_2 (0.0911 g, 1.05 mmol) was loaded. The mixture was stirred at r.t. for 10 min, filtered through Celite, and concentrated under reduced pressure. The residual material was taken up in CH_2Cl_2 (100 mL), washed with water (100 mL × 3), dried over anhyd $MgSO_4$, filtered, and concentrated under reduced pressure. Flash column chromatography on SiO_2 (CH_2Cl_2:EtOAc = 100:1 to 1:2 → CH_2Cl_2:EtOAc:MeOH = 45:50:5, v/v) furnished **C-P4** as a red solid (71.3 mg, 91.9 μmol, yield = 92%). ^{1}H NMR (500 MHz, $CDCl_3$, 28.2 mM, 298 K): δ 10.34 (s, 1H), 8.12–7.97 (br, 2H), 7.82 (d, J = 8.0 Hz, 1H), 7.73–7.67 (m, 2H), 7.67–7.58 (m, 4H), 7.54 (d, J = 8.8 Hz, 1H), 7.46 (d, J = 7.4 Hz, 1H), 7.40 (t, J = 7.4 Hz, 1H), 7.31 (dd, J = 14.1, 6.1 Hz, 1H), 7.25 (d, J = 6.9 Hz, 1H), 7.50–7.22 (br, 1H), 6.65– 6.47 (br, 2H), 6.41– 5.95 (br, 3H), 3.90–3.50 (br, 8H), 3.48 (s, 3H), 3.47 (s, 3H), 3.35– 3.24 (br, 2H), 2.68–2.54 (br, 2H), 2.47–2.39 (m, 1H), 2.15–2.06 (m, 1H). ^{13}C NMR (125 MHz, $CDCl_3$, 20.0 mM, 298 K): δ 160.29, 159.73, 153.23, 153.15, 143.59, 143.35, 136.80, 136.67, 136.13, 130.99, 130.60, 130.32, 129.84, 129.65, 129.53, 127.80, 127.77, 127.05, 126.62, 126.45, 125.50, 124.91, 124.87, 124.76, 124.38, 124.27, 124.23, 123.99, 123.95, 123.73, 123.53, 122.99, 119.25, 119.05, 118.56, 118.04, 114.05, 104.17, 70.64, 70.62, 66.88, 66.80, 59.28, 59.26, 32.22, 23.30, 21.77. FT-IR (ATR, cm^{-1}): 3044, 2923, 1732, 1587, 1556, 1472, 1449, 1392, 1261, 1217, 1197, 1124, 1055, 1030, 880. HRMS (ESI) calcd for $C_{50}H_{42}N_5O_4$ [M + H]$^+$ 776.3231, found 776.3235.

2-(4,7-Bis(5-(2-methoxyethoxy)pyridin-2-yl)-1,2',3,3',4',9'-hexahydrospiro[benzo[*d*] imidazole-2,1'-carbazol]-8'-yl)-[*H*]-benzo[*de*]isoquinoline-1,3(2*H*)-dione (6-NI). An oven-dried 100 mL round-bottom flask was loaded with **4** (0.191 g, 0.502 mmol), **5** (0.206 g, 0.502 mmol), *p*-toluenesulfonic acid monohydrate (10.9 mg, 57.3 μmol), and anhyd EtOH (95 mL). The mixture was heated at reflux for 14 h, cooled to r.t., and concentrated under reduced pressure. Flash column chromatography on SiO_2 (CH_2Cl_2:EtOAc = 100:1 to 1:1 → CH_2Cl_2:EtOAc:MeOH = 45:50:5 v/v) furnished **6-NI** as a yellow solid (0.108 g, 0.140 mmol, yield = 28%). ^{1}H NMR (850 MHz, CD_2Cl_2, 298 K) δ 8.57–8.45 (m, 3H), 8.27–8.19 (m, 4H), 7.72 (t, J = 7.7 Hz, 2H), 7.69 (d, J = 8.1 Hz, 1H), 7.63 (d, J = 8.9 Hz, 2H), 7.50 (s, 2H), 7.27–7.21 (m, 3H), 7.12 (d, J = 7.4 Hz, 1H), 6.96 (s, 2H), 4.14–4.10 (m, 4H), 3.71–3.67 (m, 4H), 3.39 (s, 6H), 2.88 (t, J = 6.1 Hz, 2H), 2.24–2.19 (m, 2H), 2.11–2.06 (m, 2H).^{13}C NMR (213 MHz, CD_2Cl_2, 298 K) δ 164.68, 153.21, 151.06, 139.00, 136.82, 136.21, 134.83, 133.71, 132.27, 132.02, 129.70, 129.15, 127.43, 123.45, 123.30, 123.04, 120.50, 120.37, 120.14, 120.03, 115.90, 115.66, 115.16, 78.68, 71.39, 68.40, 59.41, 40.20, 21.56, 21.51. FT-IR (ATR, cm^{-1}): 3328, 2923, 1736, 1707, 1677, 1666, 1589, 1501, 1462, 1261, 1235, 1128, 1057, 972, 899, 890. HRMS (ESI) calcd for $C_{46}H_{41}N_6O_6$ [M + H]$^+$ 773.3082, found 773.3085.

4,7-Bis(5-(2-methoxyethoxy)pyridin-2-yl)-8'-(pyren-1-yl)-1,2',3,3',4',9'-hexahydrospiro[benzo[*d*]imidazole-2,1'-carbazole] (6-P1). An oven-dried 250 mL round-bottom flask was loaded with **2-P1** (0.123 g, 0.319 mmol), **5** (0.125 g, 0.304 mmol), *p*-toluenesulfonic acid monohydrate (19.1 mg, 99.9 μmol), and MeCN (95 mL). The mixture was heated at reflux for 18 h, cooled to r.t., and concentrated under reduced pressure. Flash column chromatography on SiO_2 (CH_2Cl_2:EtOAc = 100:1 to 1:1 → CH_2Cl_2:EtOAc:MeOH = 45:50:5 v/v) furnished **6-P1** as a yellow solid (75.7 mg, 97.3 μmol, yield = 31%). ^{1}H NMR (850 MHz, CD_2Cl_2, 298 K): δ 8.22 (t, J = 2.4 Hz, 2H), 8.19–8.13 (m, 3H), 8.07–8.02 (m, 4H), 7.96 (t, J = 7.5 Hz, 1H), 7.86 (q, J = 9.1 Hz, 2H), 7.70 (d, J = 7.8 Hz, 1H), 7.60 (d, J = 8.9 Hz, 1H), 7.56 (d, J = 8.9 Hz, 1H), 7.46 (d, J = 36.7 Hz, 2H), 7.36–7.30 (m, 2H), 7.23 (ddd, J = 27.8, 8.9, 3.0 Hz, 2H), 6.87 (q, J = 8.7 Hz, 2H), 4.17–4.11 (m, 4H), 3.75–3.68 (m, 4H), 3.40 (s, 3H), 3.39 (s, 3H), 2.95–2.90 (m, 2H), 2.27–2.19 (m, 2H), 2.15–2.11 (m, 2H). ^{13}C NMR (213 MHz, CD_2Cl_2, 298 K): δ 153.23, 153.20, 151.12, 151.07, 139.07, 139.00, 136.86, 136.28, 135.95, 134.49, 131.87, 131.48, 131.39, 129.49, 128.32, 128.00, 127.94, 127.90, 127.83, 126.49, 125.61, 125.60, 125.52, 125.48, 125.34, 125.22, 125.04, 122.86,

122.76, 120.39, 120.36, 120.03, 118.95, 115.90, 115.88, 115.70, 115.66, 114.35, 78.78, 71.42, 68.45, 68.43, 59.42, 40.12, 31.16, 21.62. FT-IR (ATR, cm^{-1}): 3372, 2923, 1557, 1504, 1459, 1381, 1359, 1338, 1323, 1296, 1279, 1232, 1126, 1037, 970, 843. HRMS (ESI) calcd for $C_{50}H_{44}N_5O_4$ $[M + H]^+$ 778.3388, found 778.3390.

4,7-Bis(5-(2-methoxyethoxy)pyridin-2-yl)-8'-(pyren-2-yl)-1,2',3,3',4',9'-hexahydrospiro[benzo[*d*]imidazole-2,1'-carbazole] (6-P2). An oven-dried 250 mL round-bottom flask was loaded with **2-P2** (0.116 g, 0.301 mmol), **5** (0.124 g, 0.302 mmol), *p*-toluenesulfonic acid monohydrate (6.9 mg, 36 μmol), and MeCN (100 mL). The mixture was heated at reflux for 19 h, cooled to r.t., and concentrated under reduced pressure. Flash column chromatography on SiO_2 (CH_2Cl_2:EtOAc = 100:1 to 1:1 → CH_2Cl_2:EtOAc:MeOH = 45:50:5, v/v) furnished **6-P2** as a yellow solid (77.0 mg, 99.0 μmol, yield = 33%).^{1}H NMR (400 MHz, CD_2Cl_2, 298 K): δ 8.73 (s, 1H), 8.34 (s, 2H), 8.25 (d, J = 3.0 Hz, 2H), 8.17 (d, J = 7.6 Hz, 2H), 8.03 (d, J = 9.0 Hz, 2H), 7.99 (t, J = 7.7 Hz, 1H), 7.93 (d, J = 9.0 Hz, 2H), 7.67 (d, J = 9.0 Hz, 2H), 7.64 (d, J = 7.8 Hz, 1H), 7.57 (s, 2H), 7.43 (d, J = 7.2 Hz, 1H), 7.33–7.24 (m, 3H), 6.99 (s, 2H), 4.17–4.09 (m, 4H), 3.74–3.65 (m, 4H), 3.39 (s, 6H), 2.92 (t, J = 6.0 Hz, 2H), 2.32–2.26 (m, 2H), 2.20–2.12 (m, 2H). ^{13}C NMR (100 MHz, CD_2Cl_2, 298 K): δ 153.34, 151.22, 139.39, 137.57, 137.22, 136.38, 134.88, 132.23, 131.60, 128.28, 128.21, 127.94, 126.52, 126.47, 125.64, 125.17, 124.88, 124.22, 124.15, 122.94, 120.53, 120.51, 118.91, 116.21, 116.07, 114.35, 79.09, 71.43, 68.49, 59.43, 39.99, 21.79, 21.65. FT-IR (ATR, cm^{-1}): 3366, 3042, 2925, 2890, 1591, 1504, 1449, 1461, 1383, 1336, 1281, 1266, 1233, 1129, 1062, 1041, 975, 885. HRMS (ESI) calcd for $C_{50}H_{44}N_5O_4$ $[M + H]^+$ 778.3388, found 778.3390.

4,7-Bis(5-(2-methoxyethoxy)pyridin-2-yl)-8'-(pyren-4-yl)-1,2',3,3',4',9'-hexahydrospiro[benzo[*d*]imidazole-2,1'-carbazole] (6-P4). An oven-dried 250 mL round-bottom flask was loaded with **2-P4** (0.345 g, 0.896 mmol), **5** (0.369 g, 0.900 mmol), *p*-toluenesulfonic acid monohydrate (18.1 mg, 95.2 μmol), and MeCN (50 mL). The mixture was heated at reflux for 11 h, cooled to r.t., and concentrated under reduced pressure. Flash column chromatography on SiO_2 (CH_2Cl_2:EtOAc = 100:1 to 1:1 → CH_2Cl_2:EtOAc:MeOH = 45:50:5, v/v) furnished **6-P4** as a yellow solid (0.245 g, 0.315 mmol, yield = 35%). ^{1}H NMR (850 MHz, CD_2Cl_2, 298 K): δ 8.22 (d, J = 3.0 Hz, 1H), 8.20–8.17 (m, 2H), 8.16 (d, J = 7.4 Hz, 1H), 8.11 (s, 1H), 8.10 (d, J = 7.5 Hz, 2H), 8.07–8.04 (m, 2H), 7.96 (t, J = 7.5 Hz, 1H), 7.91 (d, J = 7.7 Hz, 1H), 7.78 (t, J = 7.6 Hz, 1H), 7.72 (d, J = 8.1 Hz, 1H), 7.58 (d, J = 8.9 Hz, 1H), 7.56 (d, J = 8.9 Hz, 1H), 7.47 (s, 1H), 7.40 (s, 1H), 7.38 (dd, J = 7.0, 1.0 Hz, 1H), 7.32 (dd, J = 8.1, 7.1 Hz, 1H), 7.22 (td, J = 9.0, 3.0 Hz, 2H), 6.89–6.85 (m, 2H), 4.15–4.09 (m, 4H), 3.70 (ddd, J = 9.2, 5.4, 4.0 Hz, 4H), 3.39 (s, 3H), 3.39 (s, 3H), 2.94–2.91 (m, 2H), 2.25–2.19 (m, 2H), 2.15–2.11 (m, 2H). ^{13}C NMR (213 MHz, CD_2Cl_2, 298 K): δ 153.21, 153.18, 151.11, 151.06, 139.04, 138.99, 136.74, 136.39, 136.27, 136.23, 135.89, 131.88, 131.52, 131.32, 130.89, 128.92, 128.00, 127.69, 126.58, 126.32, 125.79, 125.75, 125.50, 124.84, 124.79, 124.64, 124.18, 122.82, 122.79, 120.38, 120.36, 120.01, 119.07, 115.91, 115.89, 115.72, 115.69, 114.36, 78.80, 71.40, 68.42, 68.41, 59.41, 40.09, 21.62, 21.60. FT-IR (ATR, cm^{-1}): 3401, 2925, 1593, 1504, 1459, 1381, 1359, 1323, 1270, 1233, 1128, 1041, 924, 822. HRMS (ESI) calcd for $C_{50}H_{44}N_5O_4$ $[M + H]^+$ 778.3388, found 778.3393.

8-(Pyren-1-yl)-2,3,4,9-tetrahydro-1*H*-carbazol-1-one (2-P1). A mixture of **2** (0.261 g, 0.989 mmol), pyrene-1-boronic acid (0.300 g, 1.22 mmol), $Pd(PPh_3)_4$ (58.0 mg, 50.1 μmol), and K_2CO_3 (0.562 g, 4.07 mmol) in THF (30 mL) and H_2O (10 mL) was bubbled with Ar for 5 min. The mixture was heated at reflux for 11 h, cooled to r.t., filtered through Celite, and concentrated under reduced pressure. The residual material was taken up in CH_2Cl_2 (150 mL), washed with water (100 mL × 3), dried over anhyd $MgSO_4$, filtered, and concentrated under reduced pressure. Flash column chromatography on SiO_2 (hexane:CH_2Cl_2 = 1:1 to 1:10, v/v) furnished **2-P1** as a white

solid (0.316 g, 0.820 mmol, yield = 83%). ^{1}H NMR (400 MHz, $CDCl_3$, 298 K): δ 8.72 (s, 1H), 8.28–8.21 (m, 2H), 8.16–8.09 (m, 3H), 8.08–8.00 (m, 2H), 7.96–7.87 (m, 2H), 7.77 (d, J = 8.0 Hz, 1H), 7.56 (d, J = 7.1 Hz, 1H), 7.39 (dd, J = 8.0, 7.2 Hz, 1H), 2.96–2.87 (m, 2H), 2.40–2.32 (m, 2H), 2.18–2.10 (m, 2H). ^{13}C NMR (100 MHz, $CDCl_3$, 298 K): δ 190.99, 137.31, 132.80, 131.55, 131.46, 131.25, 131.03, 129.67, 129.00, 128.86, 127.99, 127.89, 127.77, 127.43, 126.23, 126.19, 126.00, 125.45, 125.31, 125.09, 125.06, 124.88, 124.86, 120.89, 120.78, 37.98, 24.83, 21.41. FT-IR (ATR, cm^{-1}): 3283, 3046, 2933, 1648, 1603, 1584, 1547, 1471, 1412, 1377, 1319, 1303, 1243, 1175, 1137, 1077, 1043, 847, 830. HRMS (ESI) calcd for $C_{28}H_{19}NONa$ [M + Na]$^+$ 408.1359, found 408.1360.

8-(Pyren-2-yl)-2,3,4,9-tetrahydro-1*H*-carbazol-1-one (2-P2). A mixture of **2** (0.240 g, 0.908 mmol), pyrene-2-boronic acid pinacol ester (0.329 g, 1.00 mmol), $Pd(PPh_3)_4$ (58.4 mg, 50.5 μmol), and K_2CO_3 (0.699 g, 5.06 mmol) in THF (30 mL) and H_2O (10 mL) was bubbled with Ar for 5 min. The mixture was heated at reflux for 20 h, cooled to r.t., filtered through Celite, and concentrated under reduced pressure. The residual material was taken up in CH_2Cl_2 (150 mL), washed with water (100 mL × 3), dried over anhyd $MgSO_4$, filtered, and concentrated under reduced pressure. Flash column chromatography on SiO_2 (hexane:CH_2Cl_2 = 5:1 to 1:5, v/v) furnished **2-P2** as a white solid (0.284 g, 0.737 mmol, yield = 81%). ^{1}H NMR (400 MHz, $CDCl_3$, 298 K): δ 9.09 (s, 1H), 8.38 (s, 2H), 8.23 (d, J = 7.6 Hz, 2H), 8.14 (d, J = 9.0 Hz, 2H), 8.10 (d, J = 9.0 Hz, 2H), 8.05 (t, J = 7.6 Hz, 1H), 7.72 (d, J = 8.0 Hz, 1H), 7.62 (d, J = 7.0 Hz, 1H), 7.36 (t, J = 7.6 Hz, 1H), 3.05 (t, J = 6.0 Hz, 2H), 2.64–2.59 (m, 2H), 2.31–2.22 (m, 2H). ^{13}C NMR (100 MHz, $CDCl_3$, 298 K): δ 191.31, 136.34, 135.92, 131.97, 131.74, 131.26, 130.06, 128.28, 127.84, 127.48, 127.41, 126.73, 126.25, 125.52, 124.58, 124.53, 124.23, 121.32, 120.81, 38.37, 25.07, 21.63. FT-IR (ATR, cm^{-1}): 3293, 3036, 2935, 1650, 1601, 1548, 1443, 1319, 1168, 1135, 878, 813. HRMS (ESI) calcd for $C_{28}H_{19}NONa$ [M + Na]$^+$ 408.1359, found 408.1361.

8-(Pyren-4-yl)-2,3,4,9-tetrahydro-1*H*-carbazol-1-one (2-P4). A mixture of **2** (0.352 g, 1.33 mmol), pyrene-4-boronic acid pinacol ester (0.393 g, 1.20 mmol), $Pd(PPh_3)_4$ (71.0 mg, 61.4 μmol), and K_2CO_3 (0.667 g, 4.82 mmol) in THF (40 mL) and H_2O (10 mL) was bubbled with Ar for 5 min. The mixture was heated at reflux for 14 h, cooled to r.t., filtered through Celite, and concentrated under reduced pressure. The residual material was taken up in CH_2Cl_2 (150 mL), washed with water (100 mL × 3), dried over anhyd $MgSO_4$, filtered, and concentrated under reduced pressure. Flash column chromatography on SiO_2 (hexane:CH_2Cl_2 = 10:1 to 1:10, v/v) furnished **2-P4** as a white solid (0.345 g, 0.896 mmol, yield = 67%). ^{1}H NMR (500 MHz, $CDCl_3$, 298 K): δ 8.39 (s, 1H), 8.27 (d, J = 7.6 Hz, 1H), 8.24–8.18 (m, 2H), 8.17–8.13 (m, 3H), 8.07 (t, J = 7.6 Hz, 1H), 7.94–7.86 (m, 2H), 7.82 (d, J = 8.1 Hz, 1H), 7.62 (d, J = 7.0 Hz, 1H), 7.39 (t, J = 7.6 Hz, 1H), 3.11 (t, J = 6.0 Hz, 2H), 2.59 (t, J = 5.9 Hz, 2H), 2.32–2.26 (m, 2H). ^{13}C NMR (125 MHz, $CDCl_3$, 298 K): δ 191.10, 137.28, 134.90, 131.71, 131.58, 131.33, 130.81, 130.03, 129.66, 128.79, 128.51, 127.82, 127.59, 126.41, 126.39, 126.18, 125.77, 125.65, 125.60, 125.48, 125.25, 124.66, 123.62, 121.14, 120.99, 38.36, 25.10, 21.70. FT-IR (ATR, cm^{-1}): 3278, 3045, 2926, 1646, 1604, 1545, 1470, 1410, 1319, 1297, 1237, 1170, 1136, 1093, 1044, 1004, 882, 827. HRMS (ESI) calcd for $C_{28}H_{19}NONa$ [M + Na]$^+$ 408.1359, found 408.1360.

2-(1-Oxo-2,3,4,9-tetrahydro-1*H*-carbazol-8-yl)-1*H*-benzo[*de*]isoquinoline-1,3(2*H*)-dione (4). A 50 mL round-bottom flask was loaded with **3** (0.145 g, 0.725 mmol), 1,8-naphthalic anhydride (0.421 g, 2.12 mmol) and 1H-imidazole (4.003 g) in 5 mL of EtOH. The mixture was heated at 120 °C for 20 h, cooled to r.t., filtered through Celite, and concentrated under reduced pressure. The residual material was taken up in CH_2Cl_2 (150 mL), washed with aq. HCl (0.1 N, 100 mL × 3). The combined extracts were dried over anhyd. $MgSO_4$, filtered, and concentrated under reduced pressure. Flash column chromatography on SiO_2 (hexane:EtOAc = 100:1 to 1:1, v/v)

furnished **4** as a white solid (0.223 g, 0.586 mmol, yield = 81%). ^{1}H NMR (400 MHz, DMSO-d_6, 298 K): δ 11.87 (s, 1H), 8.50 (m, 4H), 7.91 (t, J = 7.7 Hz, 2H), 7.79 (d, J = 8.0 Hz, 1H), 7.33 (d, J = 7.3 Hz, 1H), 7.21 (t, J = 7.7 Hz, 1H), 3.02 (t, J = 5.8 Hz, 2H), 2.55 (t, J = 6.0, 2H), 2.22–2.14 (m, 2H). ^{13}C NMR (100 MHz, DMSO-d_6, 298 K): δ 190.66, 163.88, 135.36, 134.16, 131.54, 131.49, 130.36, 128.52, 128.32, 127.19, 126.98, 126.81, 123.47, 121.69, 121.67, 119.86, 38.20, 24.77, 21.07. FT-IR (ATR, cm^{-1}): 3263, 2925, 2958, 1709, 1666, 1643, 1624, 1586, 1546, 1474, 1435, 1379, 1354, 1323, 1237, 1190, 1167, 1028, 1000, 905, 893. HRMS (ESI) calcd for $C_{24}H_{16}N_2O_3Na$ [M + Na]$^+$ 403.1053, found 403.1056.

8-Amino-2,3,4,9-tetrahydro-1*H*-carbazol-1-one (3). This compound was prepared by adapting a literature procedure[8], but using different reagents for an improvement in the protocol. A mixture of **1** (0.661 g, 2.50 mmol), CuI (24.1 mg, 0.127 mmol), NaN_3 (0.976 g, 15.0 mmol), KOH (0.155 g, 2.76 mmol), KI (0.457 g, 2.75 mmol), and phenanthroline (45.4 mg, 0.252 mmol) in DMF (8 mL) and H_2O (6mL) was heated at 130 °C for 36 h in pressure tube, cooled to r.t., filtered through Celite, and concentrated under reduced pressure. The residual material was taken up in CH_2Cl_2 (100 mL), washed with water (100 mL × 3), dried over anhyd $MgSO_4$, filtered, and concentrated under reduced pressure. Flash column chromatography on SiO_2 (CH_2Cl_2:EtOAc = 100:1 to 1:1, v/v) furnished **3** as a brownish yellow solid (0.311 g, 1.55 mmol, yield = 62%). ^{1}H NMR (400 MHz, DMSO-d_6, 298 K): δ 11.15 (s, 1H), 6.83 (m, 2H), 6.47 (dd, J = 7.0, 1.2 Hz, 1H), 5.32 (s, 2H), 2.88 (t, J = 6.0 Hz, 2H), 2.54 (t, J = 5.9 Hz, 2H), 2.13 (m, 2H). ^{13}C NMR (100 MHz, DMSO-d_6, 298 K): δ 190.32, 134.83, 130.36, 128.45, 127.89, 125.94, 121.06, 108.51, 107.95, 38.06, 24.79, 21.09. FT-IR (ATR, cm^{-1}): 3266, 2957, 2923, 2853, 1737, 1633, 1545, 1481, 1467, 1398, 1375, 1286, 1179, 1136, 1091, 1017, 909, 894, 854. HRMS (ESI) calcd for $C_{12}H_{13}N_2O$ [M + H]$^+$ 201.1022, found 201.1025.

Supplementary Scheme 2. Synthetic route to **C-P2Me**, and **C-NIMe**.

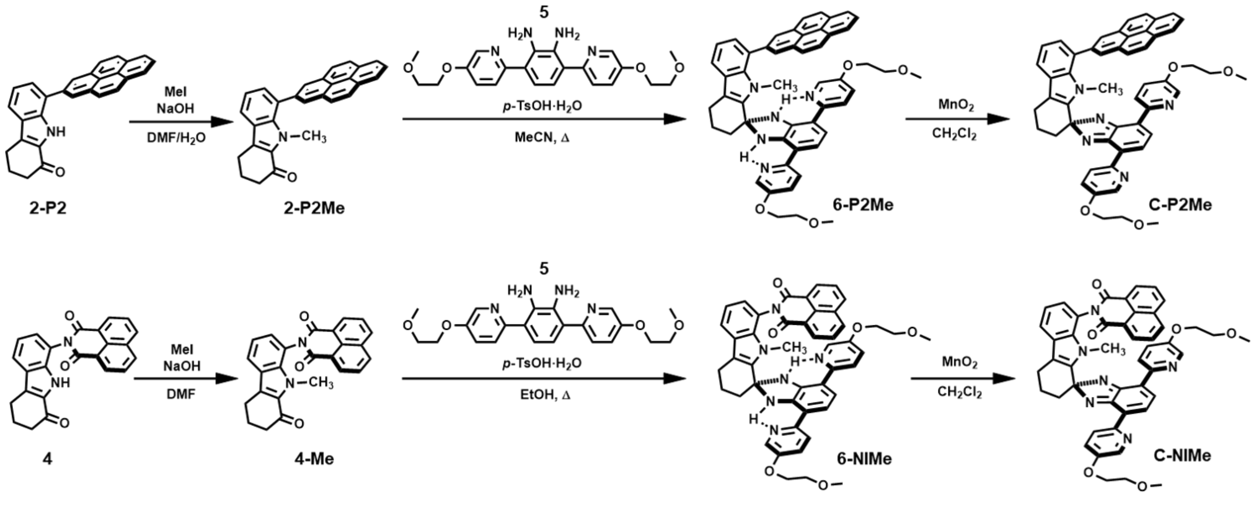

2-(4,7-Bis(5-(2-methoxyethoxy)pyridin-2-yl)-9'-methyl-2',3',4',9'-tetrahydrospiro[benzo[*d*]imidazole-2,1'-carbazol]-8'-yl)-[*H*]-benzo[*de*]isoquinoline-1,3(2*H*)-dione (C-NIMe). To a CH_2Cl_2 solution (10 mL) of **6-NIMe** (78.6 mg, 99.9 μmol), MnO_2 (86.9 mg, 1.00 mmol) was loaded. The mixture was stirred at r.t. for 10 min, filtered through Celite, and concentrated under reduced pressure. The residual material was taken up in CH_2Cl_2 (100 mL), washed with water (100 mL × 3), dried over anhyd $MgSO_4$, filtered, and concentrated

under reduced pressure. Flash column chromatography on SiO_2 (CH_2Cl_2:EtOAc = 100:1 to 1:2 → CH_2Cl_2:EtOAc:MeOH = 45:50:5, v/v) furnished **C-NIMe** as a red solid (67.5 mg, 86.0 μmol, yield = 86%). ^{1}H NMR (400 MHz, $CDCl_3$, 298 K): δ 8.79 (d, J = 8.8 Hz, 2H), 8.57 (dd, J = 7.3, 0.9 Hz, 2H), 8.36 (d, J = 2.8 Hz, 2H), 8.20 (dd, J = 8.3, 0.8 Hz, 2H), 8.13 (s, 2H), 7.76–7.68 (m, 3H), 7.28–7.20 (m, 3H), 7.01 (dd, J = 7.4, 1.0 Hz, 1H), 4.19 (dd, J = 5.3, 3.8 Hz, 4H), 3.78 (dd, J = 5.4, 3.8 Hz, 4H), 3.46 (s, 6H), 3.14 (t, J = 6.0 Hz, 2H), 2.65 (s, 3H), 2.46–2.37 (m, 2H), 1.98–1.91 (m, 2H).^{13}C NMR (100 MHz, $CDCl_3$, 298 K): δ 164.76, 161.23, 154.98, 145.13, 138.57, 134.47, 133.52, 132.86, 132.79, 132.05, 131.90, 129.88, 128.69, 128.10, 127.13, 125.96, 123.88, 122.57, 120.68, 120.05, 119.27, 119.14, 114.75, 105.65, 70.94, 67.89, 59.45, 36.99, 30.85, 23.91, 22.34. FT-IR (ATR, cm^{-1}): 2927, 1736, 1707, 1671, 1552, 1475, 1454, 1347, 1274, 1261, 1235, 1124, 1055, 900, 845. HRMS (ESI) calcd for $C_{47}H_{41}N_6O_6$ [M + H]$^+$ 785.3082, found 785.3087.

4,7-Bis(5-(2-methoxyethoxy)pyridin-2-yl)-9'-methyl-8'-(pyren-2-yl)-2',3',4',9'-tetrahydrospiro[benzo[*d*]imidazole-2,1'-carbazole] (C-P2Me). To a CH_2Cl_2 solution (10 mL) of **6-P2Me** (11.9 mg, 15.0 μmol), MnO_2 (19.1 mg, 0.220 mmol) was loaded. The mixture was stirred at r.t. for 10 min, filtered through Celite, and concentrated under reduced pressure. The residual material was taken up in CH_2Cl_2 (100 mL), washed with water (100 mL × 3), dried over anhyd $MgSO_4$, filtered, and concentrated under reduced pressure. Flash column chromatography on SiO_2 (CH_2Cl_2:EtOAc = 100:1 to 1:2 → CH_2Cl_2:EtOAc:MeOH = 45:50:5, v/v) furnished **C-P2Me** as a red solid (10.3 mg, 13.0 μmol, yield = 87%). ^{1}H NMR (850 MHz, $CDCl_3$, 298 K): δ 8.74 (d, J = 8.8 Hz, 2H), 8.36 (d, J = 3.0 Hz, 2H), 8.15–8.11 (m, 4H), 8.09 (s, 2H), 8.02 (d, J = 8.9 Hz, 2H), 7.98–7.93 (m, 3H), 7.72 (dd, J = 8.0, 1.1 Hz, 1H), 7.24 (dd, J = 8.8, 3.0 Hz, 2H), 7.22–7.20 (m, 1H), 7.18 (dd, J = 7.0, 1.2 Hz, 1H), 4.22–4.17 (m, 4H), 3.79–3.75 (m, 4H), 3.46 (s, 6H), 3.21 (t, J = 6.3 Hz, 2H), 2.51–2.46 (m, 2H), 2.29 (s, 3H), 2.05–2.00 (m, 2H). ^{13}C NMR (213 MHz, $CDCl_3$, 298 K) δ 161.21, 154.80, 145.11, 138.50, 138.25, 135.81, 132.94, 132.64, 131.01, 130.46, 127.89, 127.84, 127.80, 127.22, 126.48, 126.16, 126.10, 125.83, 125.54, 125.11, 124.45, 123.54, 120.67, 118.55, 118.22, 114.54, 105.60, 70.80, 67.74, 59.31, 36.77, 33.03, 23.86, 22.21. The resonance of one aromatic carbon was not found and it is presumably overlaped. FT-IR (ATR, cm^{-1}): 3053, 2927, 2856, 1731, 1654, 1586, 1553, 1449, 1475, 1397, 1370, 1236, 1125, 1096, 1020, 965, 843.HRMS (ESI) calcd for $C_{51}H_{44}N_5O_4$ [M + H]$^+$ 790.3388, found 790.3388.

2-(4,7-Bis(5-(2-methoxyethoxy)pyridin-2-yl)-9'-methyl-1,2',3,3',4',9'-hexahydrospiro[benzo[*d*]imidazole-2,1'-carbazol]-8'-yl)-[*H*]-benzo[*de*]isoquinoline-1,3(2*H*)-dione (6-NIMe). An oven-dried 100 mL round-bottom flask was loaded with **4-Me** (0.0990 g, 0.251 mmol), **5** (0.115 g, 0.281 mmol), *p*-toluenesulfonic acid monohydrate (6.3 mg, 33 μmol), and anhyd EtOH (40 mL). The mixture was heated at reflux for 20 h, cooled to r.t., and concentrated under reduced pressure. Flash column chromatography on SiO_2 (CH_2Cl_2:EtOAc = 100:1 to 1:1 → CH_2Cl_2:EtOAc:MeOH = 45:50:5, v/v) furnished **6-NIMe** as a yellow solid (78.6 mg, 99.9 μmol, yield = 40%). ^{1}H NMR (400 MHz, CD_2Cl_2, 298 K): δ 8.55 (d, J = 7.3 Hz, 2H), 8.27–8.19 (m, 4H), 7.73 (t, J = 7.8 Hz, 2H), 7.69 (d, J = 7.9 Hz, 1H), 7.64 (d, J = 8.9 Hz, 2H), 7.54 (s, 2H), 7.26 (dd, J = 8.9, 3.0 Hz, 2H), 7.21 (t, J = 7.7 Hz, 1H), 7.03 (d, J = 7.4 Hz, 1H), 6.94 (s, 2H), 4.18–4.13 (m, 4H), 3.73 (dd, J = 5.3, 3.8 Hz, 4H), 3.49 (s, 3H), 3.41 (s, 6H), 2.88 (t, J = 5.9 Hz, 2H), 2.30–2.24 (m, 2H), 2.08– 2.01 (m, 2H). ^{13}C NMR (100 MHz, CD_2Cl_2, 298 K): δ 165.29, 153.17, 151.18, 138.87, 136.20, 135.98, 134.99, 133.97, 132.36, 132.24, 129.52, 129.10, 127.52, 124.39, 123.07, 123.00, 120.34, 120.31, 120.25, 119.50, 115.63, 115.14, 114.94, 79.10, 71.44, 68.49, 59.43, 43.48, 32.36, 22.22, 21.58. FT-IR (ATR, cm^{-1}): 3364, 2925, 2852, 1707, 1670, 1587, 1504, 1458, 1366, 1345, 1267, 1234, 1125, 1057, 1028, 900, 845. HRMS (ESI) calcd for $C_{47}H_{43}N_6O_6$ [M + H]$^+$ 787.3239, found 787.3241.

4,7-Bis(5-(2-methoxyethoxy)pyridin-2-yl)-9'-methyl-8'-(pyren-2-yl)-1,2',3,3',4',9'-hexahydrospiro[benzo[*d*]imidazole-2,1'-carbazole] (6-P2Me). An oven-dried 100 mL round-bottom flask was loaded with **2-P2Me** (0.120 g, 0.300 mmol), **5** (0.124 g, 0.301 mmol), *p*-toluenesulfonic acid monohydrate (4.9 mg, 25 μmol), and MeCN (40 mL). The mixture was heated at reflux for 19 h, cooled to r.t., and concentrated under reduced pressure. Flash column chromatography on SiO_2 (CH_2Cl_2:EtOAc = 100:1 to 1:1 → CH_2Cl_2:EtOAc:MeOH = 45:50:5, v/v) furnished **6-P2Me** as a yellow solid (11.9 mg, 15.0 μmol, yield = 5%). ^{1}H NMR (400 MHz, CD_2Cl_2, 298 K): δ 8.26 (d, J = 2.9 Hz, 2H), 8.20 (s, 2H), 8.16 (d, J = 7.6 Hz, 2H), 8.06 (d, J = 9.0 Hz, 2H), 8.03–7.95 (m, 3H), 7.67–7.61 (m, 3H), 7.53 (s, 2H), 7.26 (dd, J = 8.9, 3.0 Hz, 2H), 7.21–7.16 (m, 2H), 6.91 (s, 2H), 4.19–4.13 (m, 4H), 3.76–3.71 (m, 4H), 3.42 (s, 6H), 3.12 (s, 3H), 2.92 (t, J = 5.9 Hz, 2H), 2.34–2.28 (m, 2H), 2.13–2.06 (m, 2H). ^{13}C NMR (100 MHz, CD_2Cl_2, 298 K): δ 153.20, 151.33, 139.44, 139.11, 136.34, 136.12, 136.11, 131.61, 130.99, 128.28, 127.88, 127.83, 127.44, 126.89, 126.63, 126.41, 125.61, 124.95, 124.01, 122.89, 120.32, 118.87, 118.83, 115.66, 115.10, 114.48, 79.22, 71.47, 68.52, 59.46, 43.62, 35.07, 22.26, 21.75. FT-IR (ATR, cm^{-1}): 3364, 2923, 2853, 1736, 1591, 1504, 1450, 1397, 1364, 1265, 1226, 1123, 1056, 1031, 922, 895, 881. HRMS (ESI) calcd for $C_{51}H_{46}N_5O_4$ [M + H]$^+$ 792.3544, found 792.3546.

9-Methyl-8-(pyren-2-yl)-2,3,4,9-tetrahydro-1*H*-carbazol-1-one (2-P2Me). A 50 mL round-bottom flask was loaded with **2-P2** (0.155 g, 0.401 mmol), MeI (0.1 mL, 1.6 mmol) and NaOH (0.113 g, 2.00 mmol) in DMF (15 mL) and H_2O (1 mL). The mixture was stirred at r.t. for 2h, filtered through Celite, and concentrated under reduced pressure. The residual material was taken up in CH_2Cl_2 (100 mL), washed with water (100 mL × 3), dried over anhyd $MgSO_4$, filtered, and concentrated under reduced pressure. Flash column chromatography on SiO_2 (hexane:EtOAc = 50:1 to 3:1, v/v) furnished **2-P2Me** as a white solid (0.150 g, 0.374 mmol, yield = 93%). ^{1}H NMR (500 MHz, CD_2Cl_2, 298 K): δ 8.28 (s, 2H), 8.27 (d, J = 7.7 Hz, 2H), 8.18 (d, J = 8.9 Hz, 2H), 8.13 (d, J = 8.9 Hz, 2H), 8.08 (t, J = 7.6 Hz, 1H), 7.79 (dd, J = 8.0, 0.7 Hz, 1H), 7.45 (dd, J = 7.0, 0.6 Hz, 1H), 7.29 (t, J = 7.5 Hz, 1H), 3.49 (s, 3H), 3.12 (t, J = 6.1 Hz, 2H), 2.69–2.64 (m, 2H), 2.31–2.24 (m, 2H). ^{13}C NMR (125 MHz, CD_2Cl_2, 298 K) δ 191.85, 137.84, 137.79, 131.73, 131.11, 130.70, 130.06, 129.70, 128.04, 127.98, 127.28, 126.24, 126.06, 125.25, 124.40, 123.70, 120.58, 119.71, 40.31, 35.32, 24.72, 21.87. FT-IR (ATR, cm^{-1}): 3039, 2940, 2860, 1651, 1598, 1537, 1454, 1424, 1409, 1376, 1313, 1252, 1222, 1187, 1141, 1074, 1060, 961, 895, 881, 842. HRMS (ESI) calcd for $C_{29}H_{21}NONa$ [M + Na]$^+$ 422.1515, found 422.1518.

2-(9-Methyl-1-oxo-2,3,4,9-tetrahydro-1*H*-carbazol-8-yl)-[*H*]-benzo[*de*]isoquinoline-1,3(2*H*)-dione (4-Me). A 50 mL round-bottom flask was loaded with **4** (0.151 g, 0.397mmol), MeI (0.1 mL, 1.6 mmol) and NaOH (0.0852 g, 1.52 mmol) in DMF (15 mL). The mixture was stirred at r.t. for 2h, filtered through Celite, and concentrated under reduced pressure. The residual material was taken up in CH_2Cl_2 (100 mL), washed with water (100 mL × 3), dried over anhyd $MgSO_4$, filtered, and concentrated under reduced pressure. Flash column chromatography on SiO_2 (CH_2Cl_2:EtOAc = 100:1 to 1:1, v/v) furnished **4-Me** as a brownish white solid (0.135 g, 0.343 mmol, yield = 86%). ^{1}H NMR (500 MHz, $CDCl_3$, 298 K): δ 8.71 (d, J = 7.3 Hz, 2H), 8.34 (d, J = 8.4 Hz, 2H), 7.85 (t, J = 8.0 Hz, 2H), 7.79 (dd, J = 7.6, 1.4 Hz, 1H), 7.31–7.23 (m, 2H), 3.96 (s, 3H), 3.07 (t, J = 6.1 Hz, 2H), 2.64 (t, J = 6.0 Hz, 2H), 2.27–2.20 (m, 2H). ^{13}C NMR (125 MHz, $CDCl_3$, 298 K): δ 192.46, 164.85, 135.81, 134.85, 132.27, 132.07, 131.03, 130.09, 128.86, 128.30, 127.92, 127.33, 122.65, 122.51, 120.68, 120.49, 40.50, 32.23, 24.65, 22.09, FT-IR (ATR, cm^{-1}): 3057, 2939, 1706, 1651, 1586, 1540, 1511, 1500, 1457, 1427, 1376, 1349, 1232, 1186, 1028, 975, 939, 895, 887, 846. HRMS (ESI) calcd for $C_{25}H_{18}N_2O_3Na$ [M + Na]$^+$ 417.1210, found 417.1212.

Supplementary Scheme 3. Synthetic route to **5**

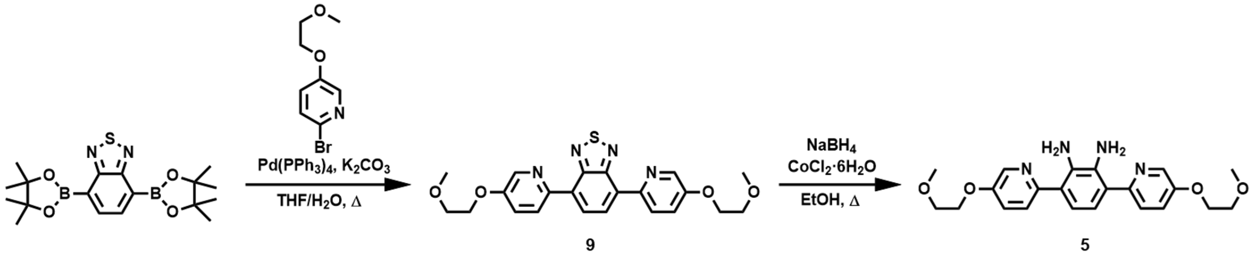

3,6-Bis(5-(2-methoxyethoxy)pyridin-2-yl)benzene-1,2-diamine (5). To a EtOH solution (250 mL) of **9** (2.63 g, 6.00 mmol), $NaBH_4$ (2.31 g, 60.1 mmol), and $CoCl_2{\cdot}6H_2O$ (0.286 g, 1.20 mmol) mixture was portionwise at r.t. for 10 min. The reaction mixture was stirred at 60 °C for 1h. After cooling down to r.t., H_2O (100 mL) was added to the reaction mixture, stirring at r.t. for 10 min, filtered through Celite, and concentrated under reduced pressure. The residual material was taken up in CH_2Cl_2 (250 mL), washed with water (150 mL × 3), dried over anhyd $MgSO_4$, filtered, and concentrated under reduced pressure. The residual material was recrystallized in EtOH (1 L) and the residual material was isolated by flash column chromatography on SiO_2 (CH_2Cl_2:EtOAc = 100:1 to 1:4 → CH_2Cl_2:EtOAc:MeOH = 45:50:5, v/v), furnishing **5** as a yellow solid (1.97 g, 4.81 mmol, yield = 80%). ^{1}H NMR (400 MHz, $CDCl_3$, 298 K): δ 8.38 (d, J = 2.8 Hz, 2H), 7.65 (d, J = 8.9 Hz, 2H), 7.34 (dd, J = 8.9, 3.0 Hz, 2H), 7.07 (s, 2H), 5.44 (s, 4H), 4.22 (dd, J = 5.3, 3.9 Hz, 4H), 3.79 (dd, J = 5.4, 3.9 Hz, 4H), 3.47 (s, 6H). ^{13}C NMR (100 MHz, $CDCl_3$, 298 K): δ 153.20, 152.39, 135.94, 135.48, 123.27, 123.06, 122.86, 118.78, 71.05, 67.97, 59.44. FT-IR (ATR, cm^{-1}): 3452, 3348, 2930, 2882, 1628, 1566, 1537, 1468, 1448, 1388, 1288, 1275, 1223, 1197, 1150, 1125, 1050, 905, 851, 834. HRMS (ESI) calcd for $C_{22}H_{27}N_4O_4$ $[M + H]^+$ 411.2027, found 411.2030.

4,7-Bis(5-(2-methoxyethoxy)pyridin-2-yl)benzothiadiazole (9). A mixture of 2,1,3-benzothiadiazole-4,7-bis(boronic acid pinacol ester) (6.33 g, 16.3 mmol), 2-bromo-5-(2-methoxyethoxy)pyridine(8.35 g, 36.0 mmol), $Pd(PPh_3)_4$ (0.93 g, 0.80 mmol), and K_2CO_3 (13.1 g, 95.0 mmol) in THF (250 mL) and H_2O (30 mL) was bubbled with Ar for 5 min. The mixture was heated at reflux for 16 h, cooled to r.t., filtrated through Celite, and concentrated under reduced pressure. The residual material was taken up in CH_2Cl_2 (200 mL), washed with water (150 mL × 3), dried over anhyd $MgSO_4$, filtered, and concentrated under reduced pressure. Flash column chromatography on SiO_2 (CH_2Cl_2:EtOAc = 100:1 to 1:4, v/v) furnished **9** as a yellow solid (5.50 g, 12.6 mmol, yield = 77%). ^{1}H NMR (500 MHz, $CDCl_3$, 298 K): δ 8.67 (d, J = 8.8 Hz, 2H), 8.51 (d, J = 2.9 Hz, 2H), 8.49 (s, 2H), 7.39 (dd, J = 8.8, 2.9 Hz, 2H), 4.24 (t, J = 4.4 Hz, 4H), 3.80 (t, J = 4.6 Hz, 4H), 3.47 (s, 6H). ^{13}C NMR (125 MHz, $CDCl_3$, 298 K): δ 154.59, 153.69, 146.84, 138.20, 131.16, 128.78, 125.31, 121.19, 70.85, 67.86, 59.25. FT-IR (ATR, cm^{-1}): 2925, 2877, 2814, 1734, 1586, 1463, 1450, 1395, 1263, 1210, 1125, 1057, 1040, 840, 828. HRMS (ESI) calcd for $C_{22}H_{23}N_4O_4S$ $[M + H]^+$ 439.1435, found 439.1437.

Supplementary Scheme 4. Synthetic route to **7**.

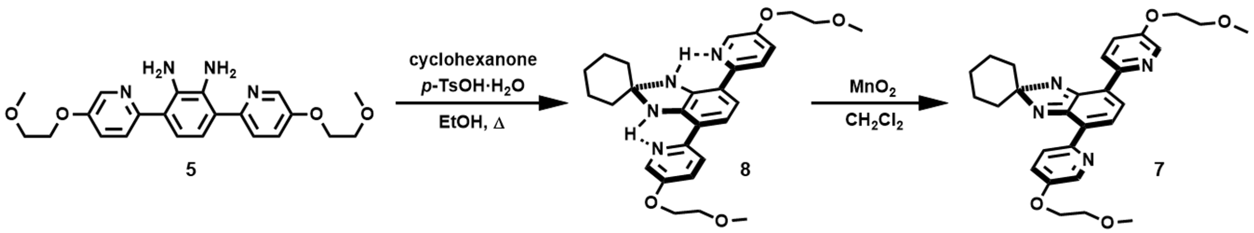

4,7-Bis(5-(2-methoxyethoxy)pyridin-2-yl)spiro[benzo[*d*]imidazole-2,1'-cyclohexane] (7). To a CH_2Cl_2 solution (6 mL) of **8** (0.0983 g, 0.200 mmol), MnO_2 (0.172 g, 1.98 mmol) was added. The mixture was stirred at r.t. for 10 min, filtered through Celite, and concentrated under reduced pressure. The residual material was taken up in CH_2Cl_2 (100 mL), washed with water (100 mL × 3), dried over anhyd $MgSO_4$, filtered, and concentrated under reduced pressure. Flash column chromatography on SiO_2 (CH_2Cl_2:EtOAc = 5:1 to 1:2, v/v) furnished **7** as a red solid (0.0830 g, 0.170 mmol, yield = 85%). ^{1}H NMR (400 MHz, $CDCl_3$, 298 K): δ 8.76 (d, J = 8.8 Hz, 2H), 8.38 (d, J = 2.3 Hz, 2H), 8.11 (s, 2H), 7.27 (dd, J = 8.8, 2.5 Hz, 2H), 4.17 (t, J = 4.0 Hz, 4H), 3.75 (t, J = 4.2 Hz, 4H), 3.43 (s, 6H), 2.00 (s, 4H), 1.84-1.63 (br, 6H). ^{13}C NMR (100 MHz, $CDCl_3$, 298 K): δ 158.90, 154.69, 145.78, 138.21, 133.12, 132.16, 125.48, 120.70, 107.51, 70.87, 67.77, 59.32, 33.30, 25.94, 25.17. FT-IR (ATR, cm^{-1}): 3060, 2928, 2854, 2819, 1735, 1586, 1553, 1476, 1394, 1261, 1215, 1125, 1058, 1034, 960, 924, 847, 831. HRMS (ESI) calcd for $C_{28}H_{33}N_4O_4$ $[M + H]^+$ 489.2496, found 489.2498.

4,7-Bis(5-(2-methoxyethoxy)pyridin-2-yl)-1,3-dihydrospiro[benzo[*d*]imidazole-2,1'-cyclohexane] (8). An oven-dried 100 mL round-bottom flask was loaded with **5** (0.132 g, 0.322 mmol), cyclohexanone (0.10 mL, 0.96 mmol), *p*-toluenesulfonic acid monohydrate (6.3 mg, 33 μmol), and anhyd EtOH (30 mL). The mixture was heated at reflux for 14 h, cooled to r.t., and concentrated under reduced pressure. Flash column chromatography on SiO_2 (CH_2Cl_2:EtOAc = 10:1 to 1:1, v/v) furnished **8** as a yellow solid (0.152 g, 0.310 mmol, yield = 96%). ^{1}H NMR (400 MHz, $CDCl_3$, 298 K): δ 8.35 (d, J = 2.9 Hz, 2H), 7.67 (d, J = 8.9 Hz, 2H), 7.32 (s, 2H), 7.27 (dd, J = 8.9, 3.0 Hz, 2H), 6.96 (s, 2H) 4.17 (dd, J = 5.4, 3.9 Hz, 4H), 3.75 (dd, J = 5.4, 3.9 Hz, 4H), 3.45 (s, 6H), 1.91–1.84 (m, 4H), 1.69 (m, 4H), 1.51 (d, J = 4.2 Hz, 2H). ^{13}C NMR (100 MHz, $CDCl_3$, 298 K): δ 152.48, 151.41, 139.97, 135.83, 122.78, 120.11, 115.36, 114.96, 80.88, 71.01, 67.93, 59.31, 39.86, 25.27, 23.73. FT-IR (ATR, cm^{-1}): 3370, 2926, 2853, 1620, 1589, 1554, 1500, 1484, 1454, 1382, 1366, 1282, 1264, 1225, 1126, 1053, 982, 846, 830. Anal. Calcd for $C_{28}H_{34}N_4O_4$: C, 68.55; H, 6.99; N, 11.42. Found: C, 68.74; H, 7.00; N, 11.10.

X-ray Crystallographic Studies on C-NI. Single crystals **C-NI** were obtained by vapor diffusion of Et_2O into a $CHCl_3$ solution of this material at r.t. An orange crystal (approximate dimensions $0.39 \times 0.041 \times 0.041$ mm^3) was placed onto a nylon loop with Paratone-N oil, and mounted on a Rigaku XtaLAB PRO single-crystal diffractometer. The data collection was carried out using Cu Kα radiation, and the crystal was kept at 93 K. A total of 30756 reflections were measured (7.312° $\leq 2\theta \leq$ 158.994°). The structure was solved with the SHELXT[9] structure solution program using intrinsic phasing, and refined with the SHELXL[10] refinement package of OLEX2[11]. A total of 8616 unique reflections were used in all calculations. The final R1 was 0.0606 ($I \geq 2\sigma(I)$), and wR2 was 0.1579 (all data). CCDC 2015415 contains the supplementary crystallographic data for this structure.

X-ray Crystallographic Studies on C-P1. Single crystals **C-P1** were obtained by vapor diffusion of pentane into a toluene solution of this material at r.t. An orange crystal (approximate dimensions $0.224 \times 0.145 \times 0.145$ mm^3) was placed onto a nylon loop with Paratone-N oil, and mounted on a Rigaku XtaLAB PRO single-crystal diffractometer. The data collection was carried out using Cu Kα radiation, and the crystal was kept at 93 K. A total of 32858 reflections were measured (7.498° $\leq 2\theta \leq$ 158.674°). The structure was solved with the SHELXT[9] structure solution program using intrinsic phasing, and refined with the SHELXL[10] refinement package of OLEX2[11]. A total of 8174 unique reflections were used in all calculations. The final $R1$ was 0.0561 ($I \geq 2\sigma(I)$), and $wR2$ was 0.1449 (all data). CCDC 2015416 contains the supplementary crystallographic data for this structure.

X-ray Crystallographic Studies on C-P2. Single crystals **C-P2** were obtained by vapor diffusion of pentane into a toluene solution of this material at r.t. An orange crystal (approximate dimensions $0.031 \times 0.028 \times 0.009$ mm^3) was coated with Parabar 10312 (Hampton Research Inc.) to mount on the micro-loop under cold nitrogen stream at $T = 200$ K. The diffraction data measured using synchrotron radiation ($\lambda = 0.70000$ Å) employing a PLSII-2D SMC on a Rayonix MX225HS CCD area detector with high precision one-axis goniostat at Pohang Accelerator Laboratory, Korea. The PAL BL2D-SMDC program[12] was used for data collection, and HKL3000sm (Ver.717)[13] was used for cell refinement, reduction, and absorption correction.A total of 37974 reflections were measured (2.408° $\leq 2\theta \leq$ 66.666°). The structure was solved with the SHELXT[9] structure solution program using intrinsic phasing, and refined with the SHELXL[10] refinement package of OLEX2[11]. A total of 12081 unique reflections were used in all calculations. The final $R1$ was 0.0604 ($I \geq 2\sigma(I)$), and $wR2$ was 0.1602 (all data). CCDC 2015418 contains the supplementary crystallographic data for this structure.

X-ray Crystallographic Studies on C-P4. Single crystals **C-P4** were obtained by vapor diffusion of pentane into a toluene solution of this material at r.t. An orange crystal (approximate dimensions $0.133 \times 0.067 \times 0.067$ mm^3) was placed onto a nylon loop with Paratone-N oil, and mounted on a Rigaku XtaLAB PRO single-crystal diffractometer. The data collection was carried out using Cu Kα radiation, and the crystal was kept at 93 K. A total of 32541 reflections were measured (7.284° $\leq 2\theta \leq$ 158.674°). The structure was solved with the SHELXT[9] structure solution program using intrinsic phasing, and refined with the SHELXL[10] refinement package of OLEX2[11]. A total of 8814 unique reflections were used in all calculations. The final $R1$ was 0.0727 ($I \geq 2\sigma(I)$), and $wR2$ was 0.2056 (all data). CCDC 2015419 contains the supplementary crystallographic data for this structure.

X-ray Crystallographic Studies on C-NIMe. Single crystals **C-NIMe** were obtained by vapor diffusion of pentane into a toluene solution of this material at r.t. An orange crystal (approximate

dimensions $0.263 \times 0.213 \times 0.056$ mm^3) was placed onto a nylon loop with Paratone-N oil, and mounted on a Rigaku XtaLAB PRO single-crystal diffractometer. The data collection was carried out using Cu Kα radiation, and the crystal was kept at 93 K. A total of 22648 reflections were measured ($8.65° \leq 2\theta \leq 158.8°$). The structure was solved with the SHELXT[9] structure solution program using intrinsic phasing, and refined with the SHELXL[10] refinement package of OLEX2[11]. A total of 7740 unique reflections were used in all calculations. The final $R1$ was 0.0658 ($I \geq 2\sigma(I)$), and $wR2$ was 0.2079 (all data). CCDC 2015417 contains the supplementary crystallographic data for this structure.

X-ray Crystallographic Studies on C-P2⊃PHD. Single crystals **C-P2⊃PHD** were obtained by vapor diffusion of pentane into a toluene solution of this material at r.t. An orange crystal (approximate dimensions $0.179 \times 0.14 \times 0.07$ mm^3) was placed onto a nylon loop with Paratone-N oil, and mounted on a Rigaku XtaLAB PRO single-crystal diffractometer. The data collection was carried out using Cu Kα radiation, and the crystal was kept at 93 K. A total of 24926 reflections were measured ($6.922° \leq 2\theta \leq 159.358°$). The structure was solved with the SHELXT[9] structure solution program using intrinsic phasing, and refined with the SHELXL[10] refinement package of OLEX2[11]. A total of 9781 unique reflections were used in all calculations. The final $R1$ was 0.1413 ($I \geq 2\sigma(I)$), and $wR2$ was 0.3549 (all data). CCDC 2015414 contains the supplementary crystallographic data for this structure.

Supplementary Table 1. Summary of X-Ray Crystallographic Data.

	**C-NI**	**C-P1**	**C-P2**
Chemical formula	$C_{46}H_{38}N_6O_6$	$C_{50}H_{41}N_5O_4$	$C_{50}H_{41}N_5O_4$
Formula weight	770.82	775.88	775.88
Crystal system	monoclinic	monoclinic	monoclinic
Space group	$P2_1/n$	$P2_1/n$	$P2_1/c$
Color of crystal	Orange	Orange	Orange
a (Å)	17.2335(3)	16.5914(1)	17.245(3)
b (Å)	10.3320(2)	13.3263(1)	15.023(3)
c (Å)	23.6721(5)	17.8764(1)	15.580(3)
α (°)	-	-	-
β (°)	104.741(2)	93.443(1)	105.00(3)
γ (°)	-	-	-
Volume (Å^3)	4076.24(14)	3945.37(4)	3898.8(14)
Z	4	4	4
R_{int}	0.0535	0.0283	0.1488
Final R indices	$R1 = 0.0606$,	$R1 = 0.0561$,	$R1 = 0.0604$,
$[I \geq 2\sigma(I)]$	$wR2 = 0.1480$	$wR2 = 0.1416$	$wR2 = 0.1218$
Final R indices	$R1 = 0.0766$,	$R1 = 0.0603$,	$R1 = 0.2280$,
[all data]	$wR2 = 0.1579$	$wR2 = 0.1449$	$wR2 = 0.1602$
GOF	1.022	1.021	0.734
	**C-P4**	**C-NIMe**	**C-P2⊃PHD**
Chemical formula	$C_{50}H_{41}N_5O_4$	$C_{47}H_{40}N_6O_6$	$C_{62}H_{47}N_7O_6$
Formula weight	775.88	784.85	986.06
Crystal system	monoclinic	triclinic	triclinic
Space group	$P2_1/c$	$P\bar{1}$	$P\bar{1}$
Color of crystal	Orange	Orange	Orange
a (Å)	17.4754(4)	9.505(1)	13.1176(2)
b (Å)	17.9987(3)	9.5066(2)	13.6193(2)
c (Å)	14.1550(3)	20.4571(4)	13.8362(2)
α (°)	-	92.118(2)	99.7900(10)
β (°)	109.854(2)	90.008(1)	100.8530(10)
γ (°)	-	93.502(1)	95.5520(10)
Volume (Å^3)	4187.60(16)	1843.79(6)	2371.04(6)
Z	4	2	2
R_{int}	0.0768	0.0390	0.1326
Final R indices	$R1 = 0.0727$,	$R1 = 0.0658$,	$R1 = 0.1413$,
$[I \geq 2\sigma(I)]$	$wR2 = 0.1905$	$wR2 = 0.1912$	$wR2 = 0.3197$
Final R indices	$R1 = 0.0916$,	$R1 = 0.0830$,	$R1 = 0.1730$,
[all data]	$wR2 = 0.2056$	$wR2 = 0.2079$	$wR2 = 0.3549$
GOF	1.073	1.081	1.049

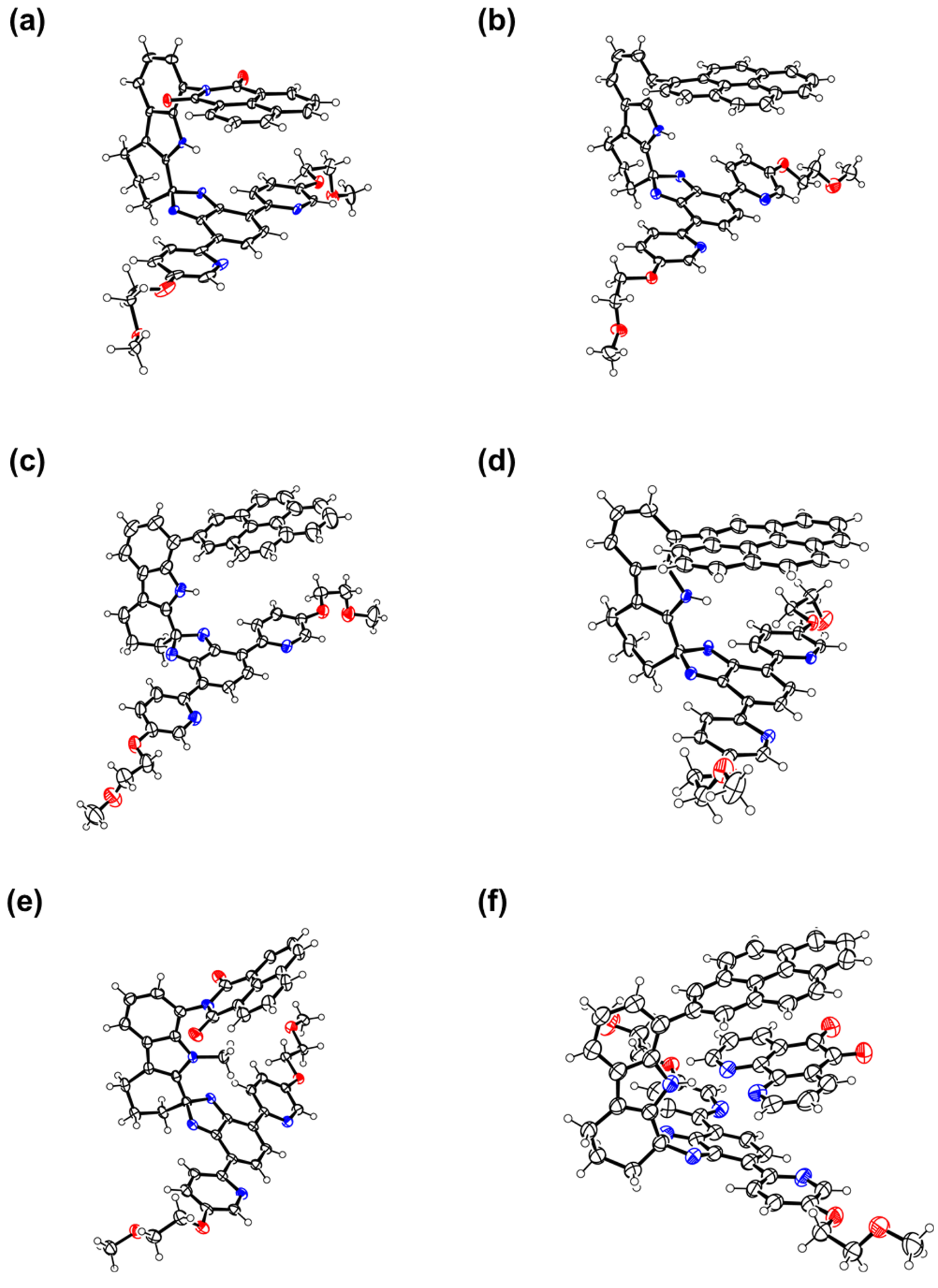

Supplementary Fig. 1. ORTEP diagrams of (a) **C-NI**, (b) **C-P1**, (c) **C-P2**, (d) **C-P4**, (e) **C-NIMe**, and (f) **C-P2⊃PHD** with thermal ellipsoids at the 50% probability level.

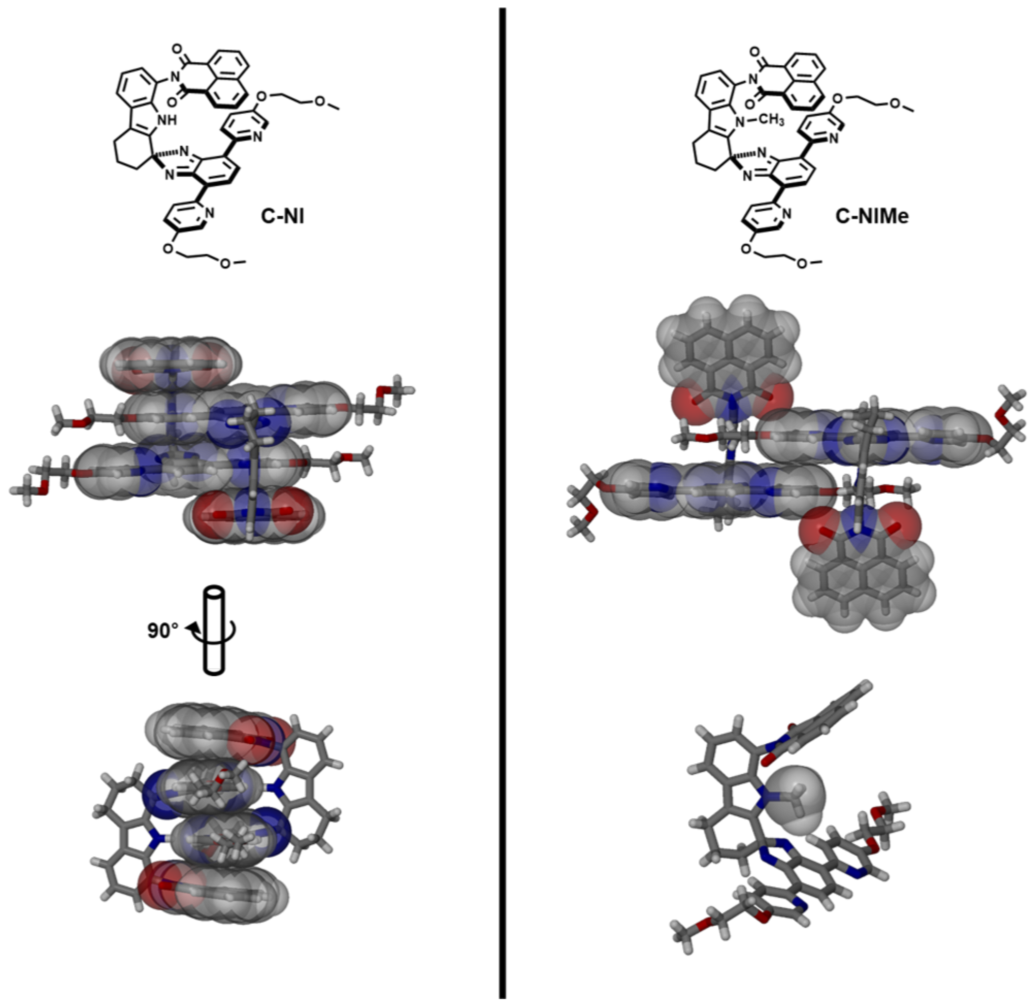

Supplementary Fig. 2. Chemical structures, and capped-stick models generated by crystallographically determined atomic coordinates of **C-NI** (left) and **C-NIMe** (right). van der Waals surfaces are overlaid to better represent laterally positioned aromatic groups, which can (for **C-NI**) and cannot (for **C-NIMe**) engage in intermolecular π–π stacking.

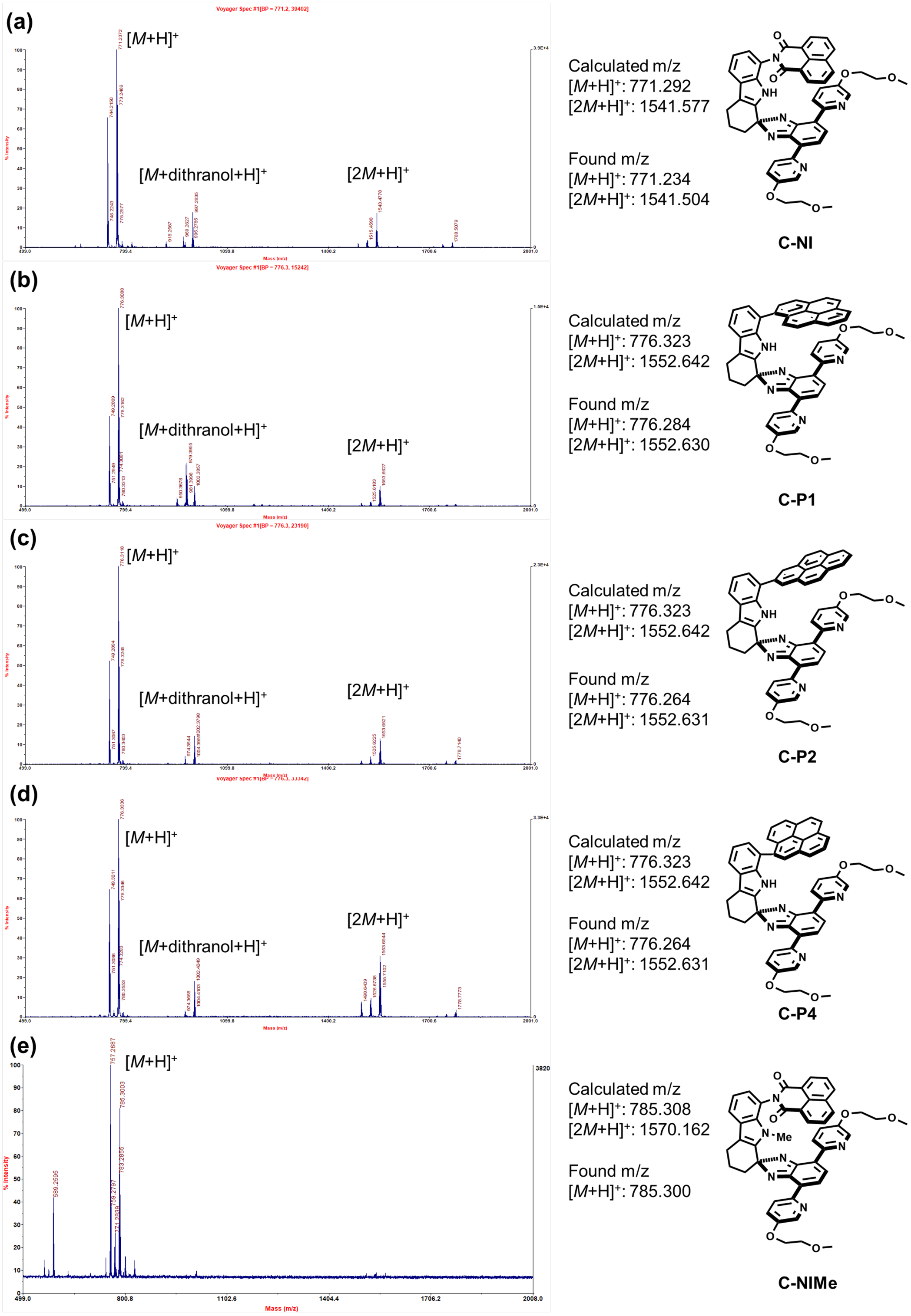

Supplementary Fig. 3. MALDI-TOF mass spectra of **C-NI**, **C-P1**, **C-P2**, **C-P4**, and **C-NIMe** with dithranol as a matrix.

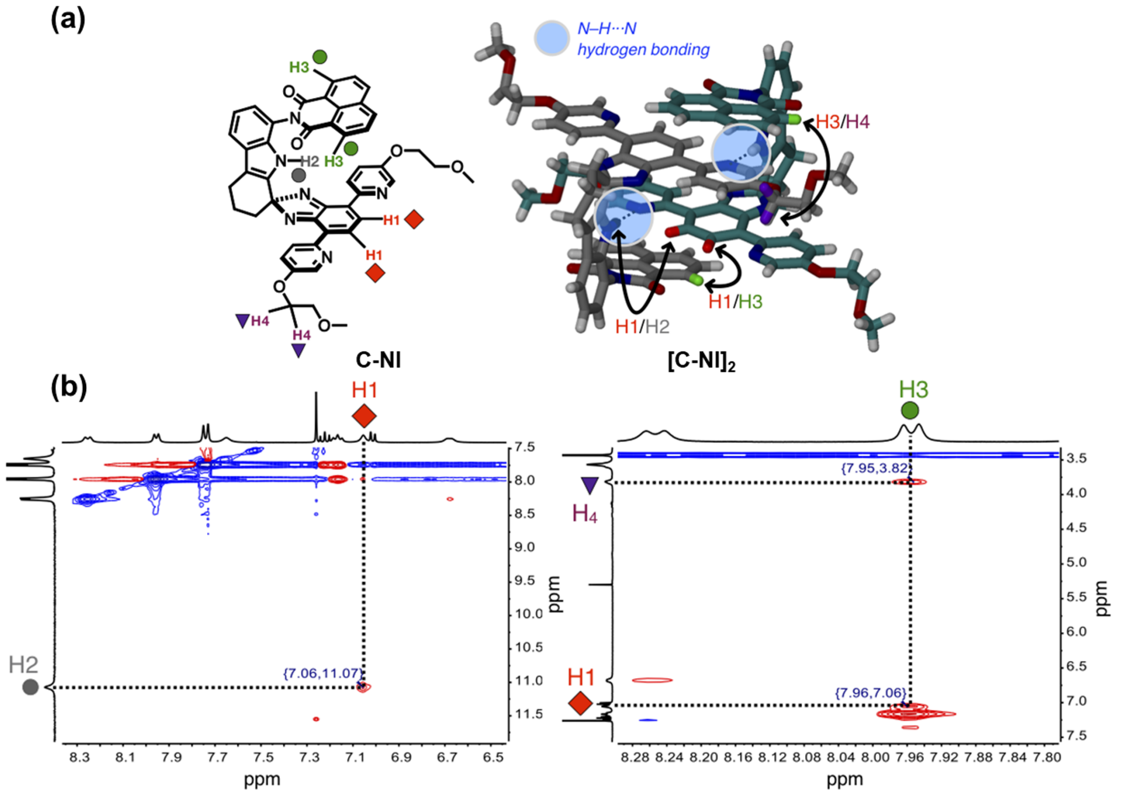

Supplementary Fig. 4. (a) Chemical structure of **C-NI** and X-ray structure of [**C-NI**]$_2$ with NOE contacts denoted with double-headed arrows. The proton resonances of isobenzimidazole (H1), indole (H2), naphthaleneimide (H3), and methoxyethyl ether chain (H4) are labeled with symbols and color-coded. Highlighted in blue shaded circles are N_{indole}–H···$N_{pyridyl}$ hydrogen bonds inside the cavity. (b) Partial 2D-ROESY contour plot of **C-NI** (13.2 mM) in $CDCl_3$ at T = 298 K; the corresponding 1D spectrum is shown along the ordinate. The proton labeling scheme is shown in (a).

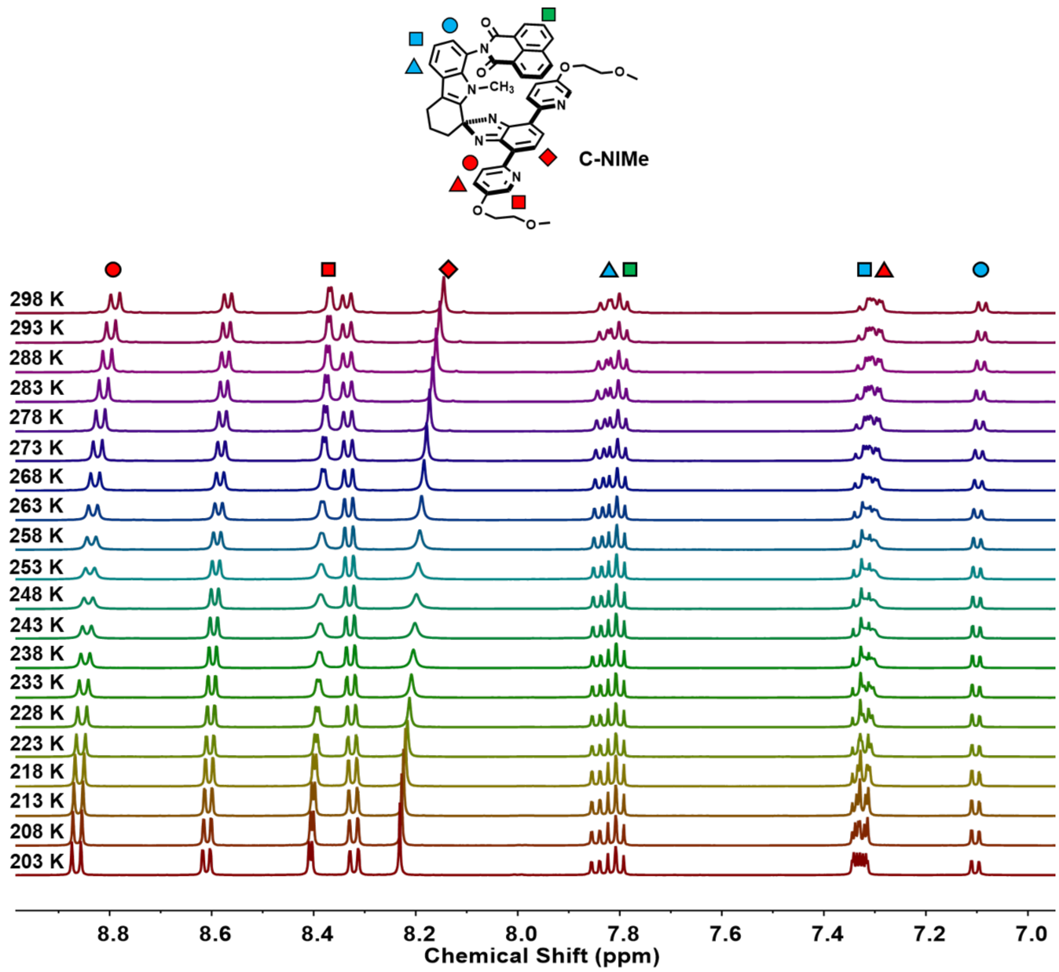

Supplementary Fig. 5. Variable-temperature (VT) ^{1}H NMR spectra of **C-NIMe** (5.1 mM) in CD_2Cl_2 at T = 203–298 K.

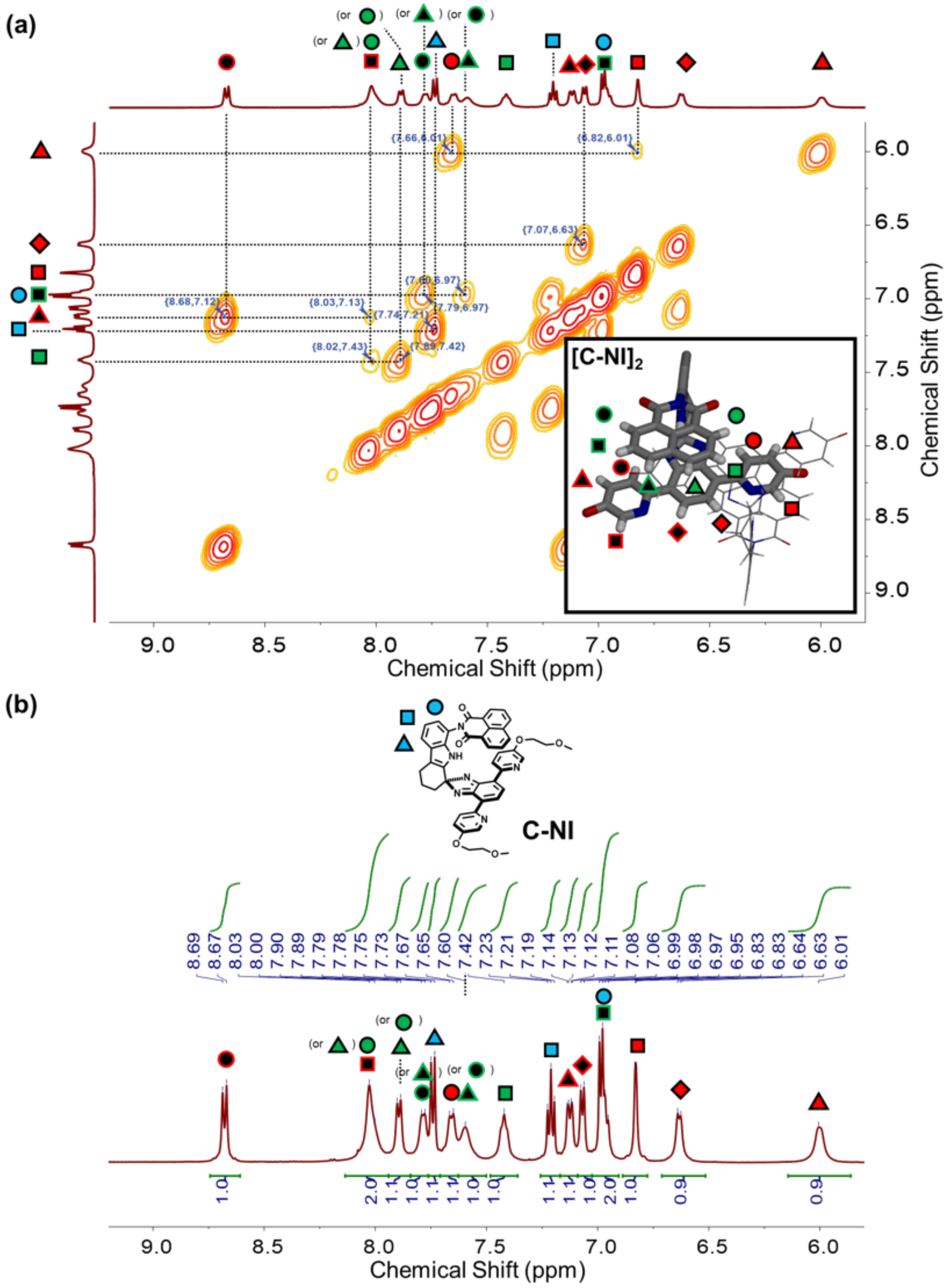

Supplementary Fig. 6. (a) Partial 2D-COSY contour plot of **C-NI** (9.8 mM) in CD_2Cl_2 at $T = 203$ K. Corresponding 1D spectrum is shown along the ordinate. Inset: capped-stick representation of dimeric [**C-NI**]$_2$ with the proton labeling scheme. (b) Partial ^{1}H NMR spectrum showing aromatic resonances and integration values.

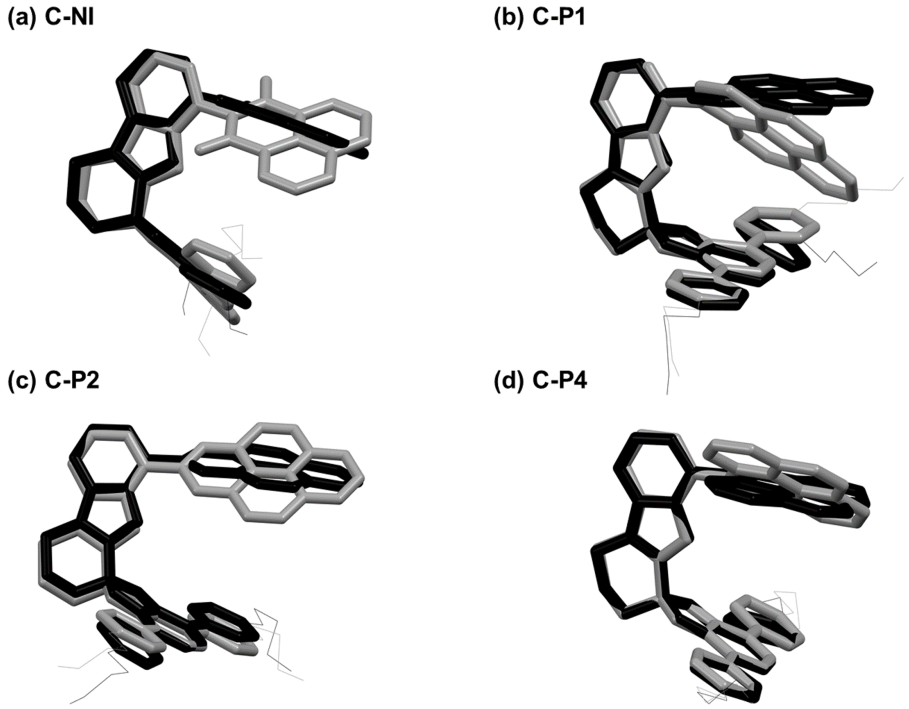

Supplementary Fig. 7. Capped-stick representations of the X-ray structures (black) of (a) **C-NI**, (b) **C-P1**, (c) **C-P2**, and (d) **C-P4** overlaid with DFT energy-minimized structures (gray). The ether chains are simplified as wireframes.

Supplementary Note 1. Determining the Dimerization (= Self-Association) Constants K_{dim}

Self-association constants were determined by fitting the changes in the chemical shifts in the ^{1}H NMR spectra as a function of sample concentration, which reflects the following equilibrium:

$$\mathsf{M} + \mathsf{M} \underset{}{\overset{K_{\mathsf{dim}}}{\rightleftarrows}} \mathsf{M}_2$$

$$K_{\mathsf{dim}} = \frac{[\mathsf{M}_2]}{[\mathsf{M}]^2}$$

$$\delta_{obs} = \left(\frac{\sqrt{1 + 8K_{\mathsf{dim}}[\mathsf{M}]_0} - 1}{4K_{\mathsf{dim}}}\right)\frac{\delta_{\mathsf{M}}}{[\mathsf{M}]_0} + 2K_{\mathsf{dim}}\left(\frac{\sqrt{1 + 8K_{\mathsf{dim}}[\mathsf{M}]_0} - 1}{4K_{\mathsf{dim}}}\right)^2\frac{\delta_{\mathsf{M}_2}}{[\mathsf{M}]_0} \tag{1}$$

Here, the terms δ_{obs}, δ_{M}, and δ_{M_2} denote the observed chemical shift (resulting from the fast exchange between M and M_2), chemical shift of the monomeric (= dissociated) M, and chemical shift of the dimeric (= self-associated) M_2, respectively.

All ^{1}H NMR (400 MHz) spectra were obtained in $CDCl_3$ to monitor concentration-dependent changes in δ_{obs} value of the $H_{\text{4-pyridine}}$ proton (denoted as red circle in the chemical structure shown next to the spectra) at T = 298 K. Numerical fittings were carried out by using non-linear least-squares regression analysis[14] with eq (1) to obtain the K_{dim} values of 8.1×10^2 M^{-1} for **C-P1**, 1.9×10^2 M^{-1} for **C-P2**, and 1.3×10^5 M^{-1} for **C-NI**, respectively. For **C-NI**, the $H_{\text{4-pyridine}}$ resonance of **C-NIMe** at δ = 8.793 ppm was approximated as that of the free (= non-dimerized) monomer. Non-linear least-squares regression analysis software was Origin2017; OriginLap Corp.: Northampton, MA.

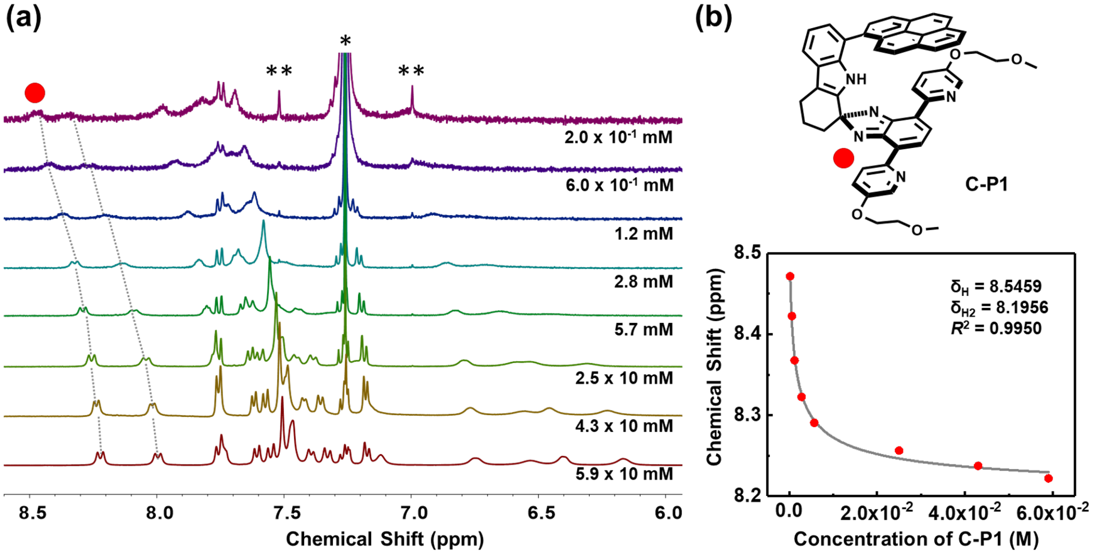

Supplementary Fig. 8. (a) Partial ^{1}H NMR spectra of **C-P1** at different concentrations in $CDCl_3$ measured at $T = 298$ K. The symbols * and ** denote residual solvent and satellite peaks, respectively. (b) Changes in the chemical shifts of the $H_{4\text{-pyridine}}$ proton resonance (●) as a function of sample concentration [**C-P1**]. The theoretical curve with $K_{\text{dim}} = 8.1 \times 10^2$ M^{-1} is superimposed on the data points.

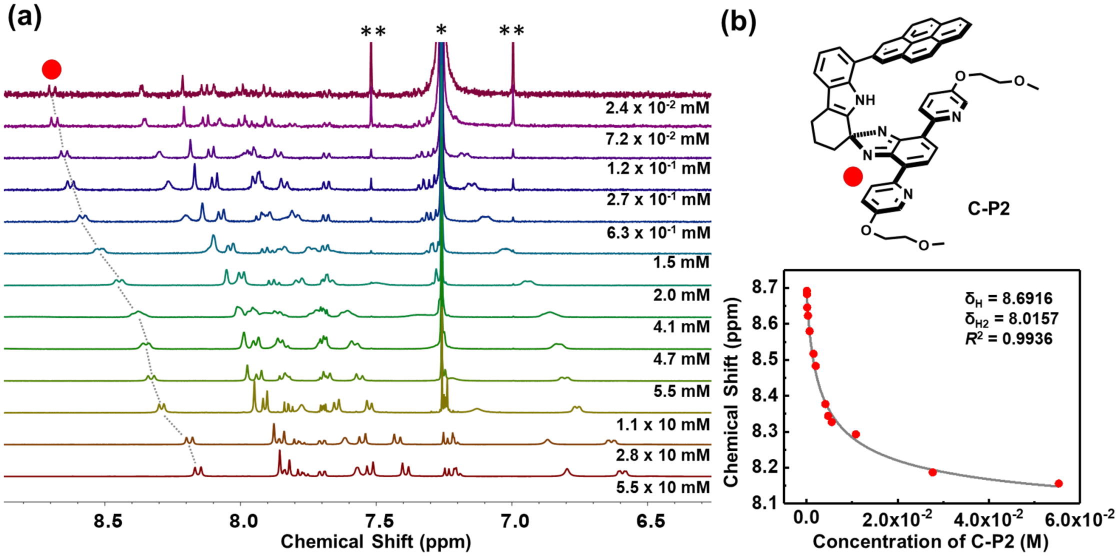

Supplementary Fig. 9. (a) Partial ^{1}H NMR spectra of **C-P2** at different concentrations in $CDCl_3$ measured at $T = 298$ K. The symbols * and ** denote residual solvent and satellite peaks, respectively. (b) Changes in the chemical shifts of the $H_{4\text{-pyridine}}$ proton resonance (●) as a function of sample concentration [**C-P2**]. The theoretical curve with $K_{\text{dim}} = 1.9 \times 10^2$ M^{-1} is superimposed on the data points.

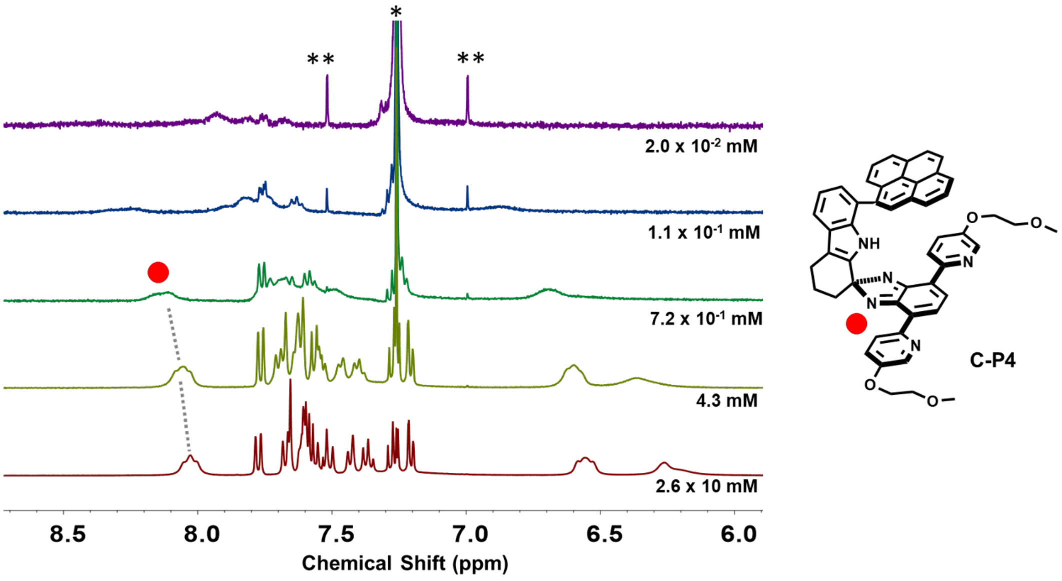

Supplementary Fig. 10. Partial ^{1}H NMR spectra of **C-P4** at different concentrations in $CDCl_3$ measured at T = 298 K. The symbols * and ** denote residual solvent and satellite peaks, respectively.

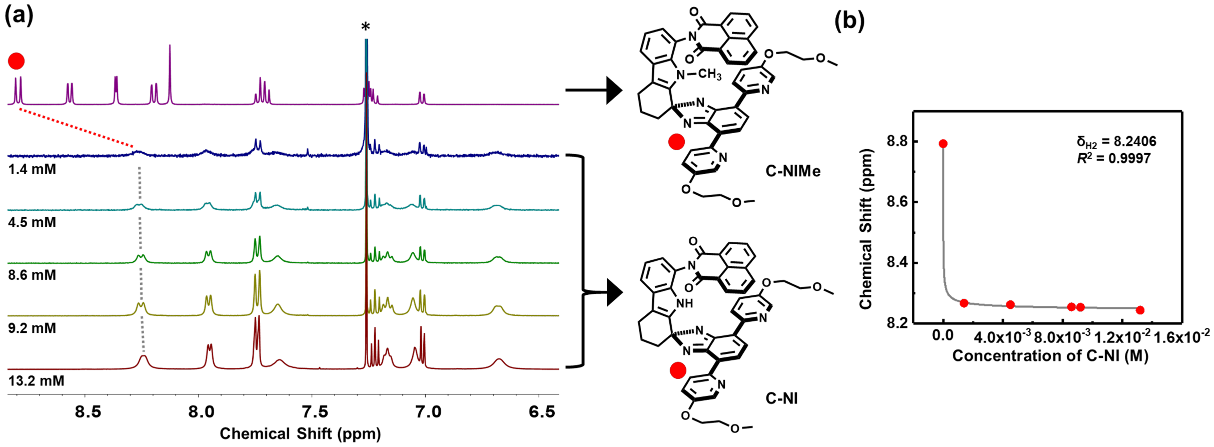

Supplementary Fig. 11. (a) Partial ^{1}H NMR spectra of **C-NI** at different concentrations in $CDCl_3$ measured at T = 298 K. The symbol * denotes residual solvent peak. (b) Changes in the chemical shifts of the $H_{4\text{-pyridine}}$ proton resonance (●) as a function of sample concentration [**C-NI**]. The theoretical curve with $K_{\text{dim}} = 1.3 \times 10^5$ M^{-1} is superimposed on the data points. The $H_{4\text{-pyridine}}$ resonance of **C-NIMe** at $\delta = 8.793$ ppm was approximated as that of the free (i.e. unbound) monomer **C-NI**.

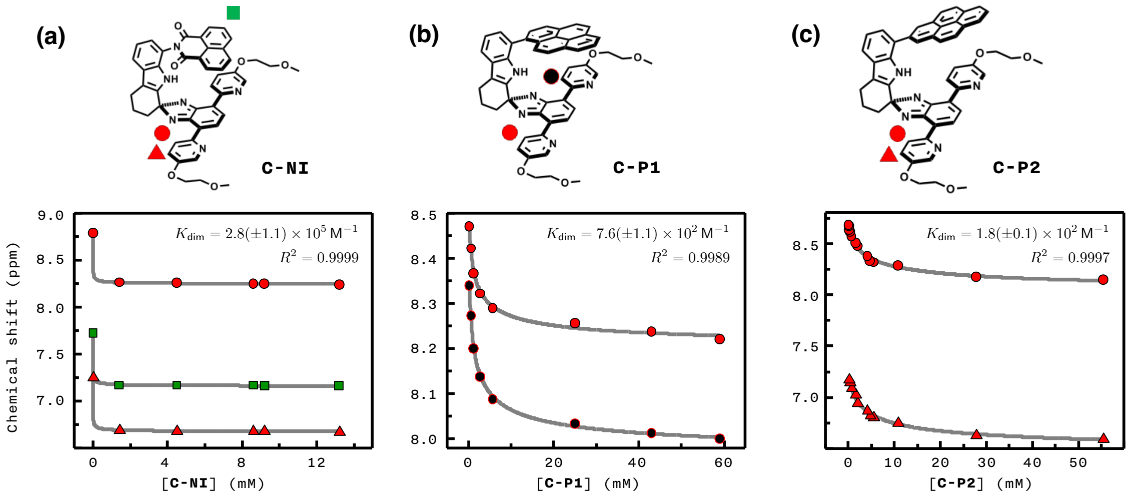

(a)

method	K (M^{-1})
global fitting	2.8 (±1.1) × 10^5
single-point fitting	1.3 (±0.8) × 10^5
Bindfit fitting	4.8 (±0.6) × 10^5

(b)

method	K (M^{-1})
global fitting	7.6 (±1.1) × 10^2
single-point fitting	8.1 (±2.3) × 10^2
Bindfit fitting	8.1 (±0.3) × 10^2

(c)

method	K (M^{-1})
global fitting	1.8 (±0.1) × 10^2
single-point fitting	1.9 (±0.3) × 10^2
Bindfit fitting	1.6 (±0.6) × 10^2

Supplementary Fig. 12. Changes in the chemical shifts of the π-clip molecules (a) **C-NI**, (b) **C-P1**, and (c) **C-P2** as a function of the concentration. In each plot, the theoretical curves with the dimerization constant K_{dim} are superimposed on the data points. Proton resonances are labeled with the symbols denoted in the chemical structures. Results from global fitting, single-point fitting, and `Bindfit` fitting are compared in the table below.

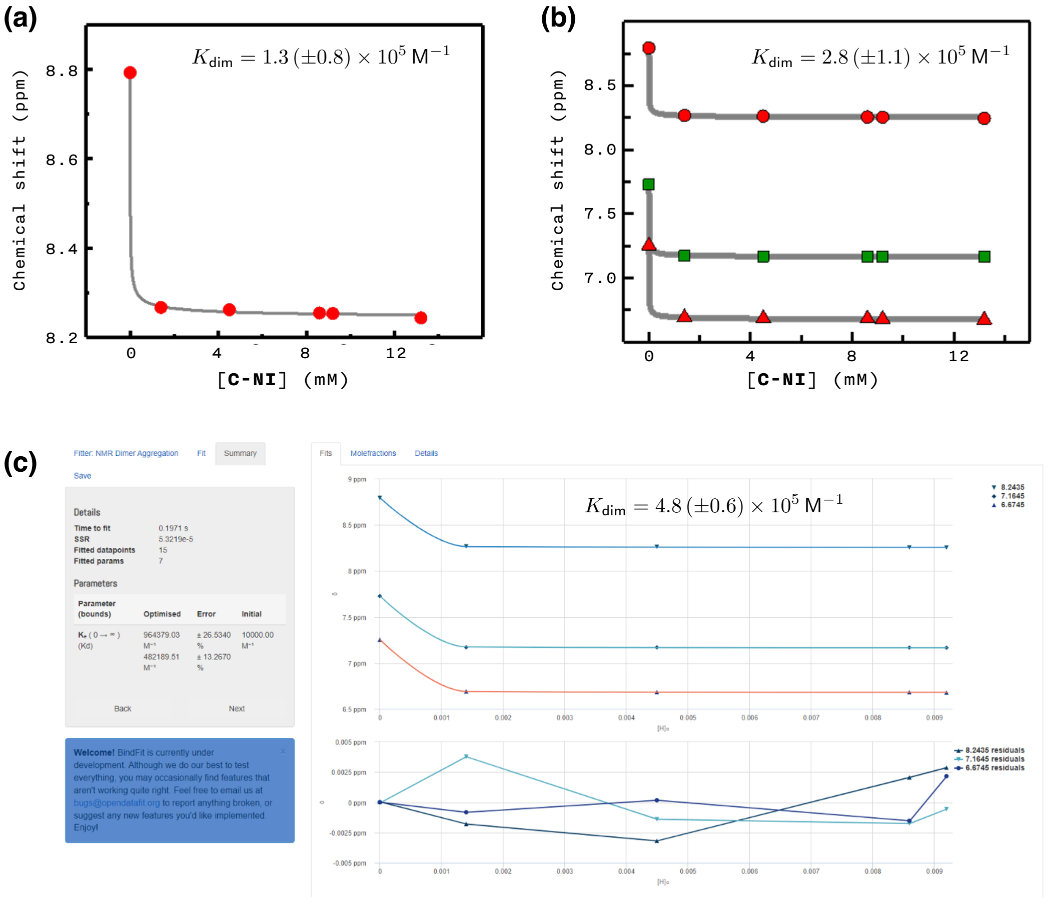

Supplementary Fig. 13. Changes in the chemical shifts of **C-NI** as a function of the concentration, modeled with (a) single-point fitting, (b) global fitting (by the graphing and data analysis software `Origin`), and (c) `Bindfit` (a web-based program). In each plot, the theoretical curve(s) with the dimerization constant K_{dim} are superimposed on the data points. Proton resonances are labeled with the symbols denoted in the chemical structure shown in Supplementary Fig. 12(a).

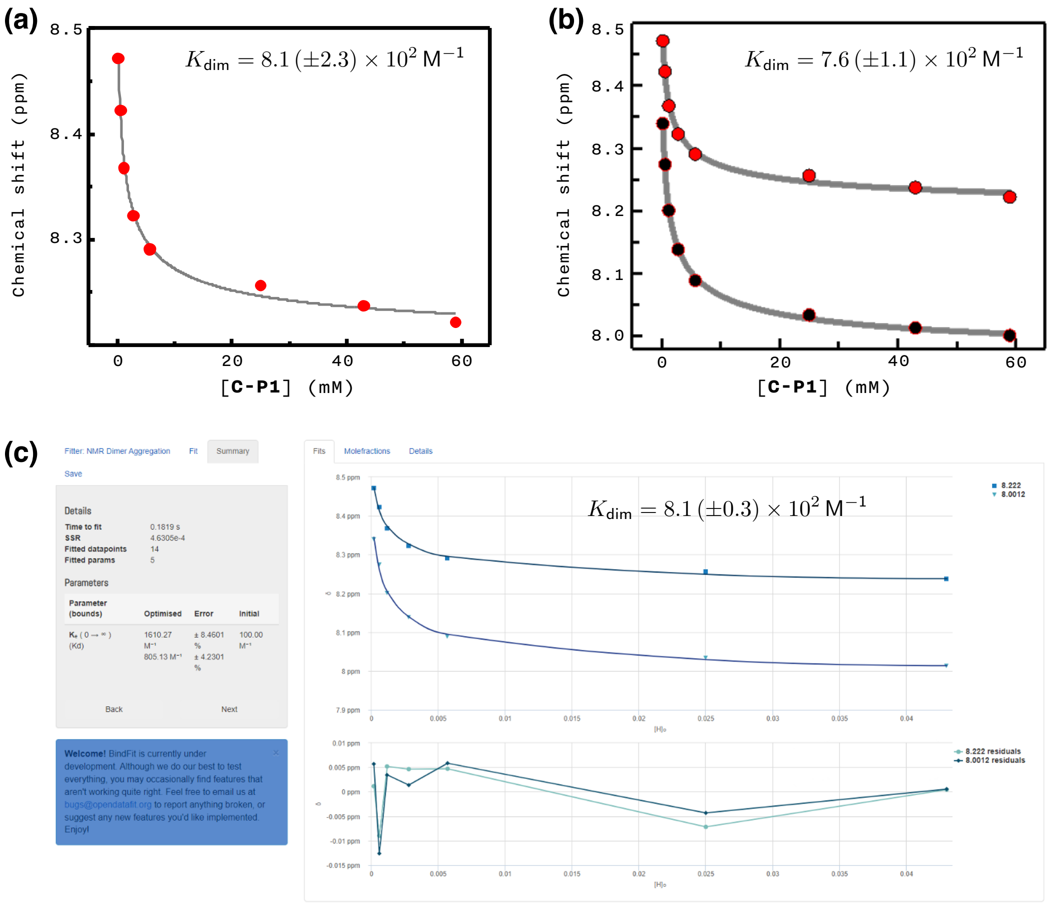

Supplementary Fig. 14. Changes in the chemical shifts of **C-P1** as a function of the concentration, modeled with (a) single-point fitting, (b) global fitting (by the graphing and data analysis software `Origin`), and (c) `Bindfit` (a web-based program). In each plot, the theoretical curve(s) with the dimerization constant K_{dim} are superimposed on the data points. Proton resonances are labeled with the symbols denoted in the chemical structure shown in Supplementary Fig. 12(b).

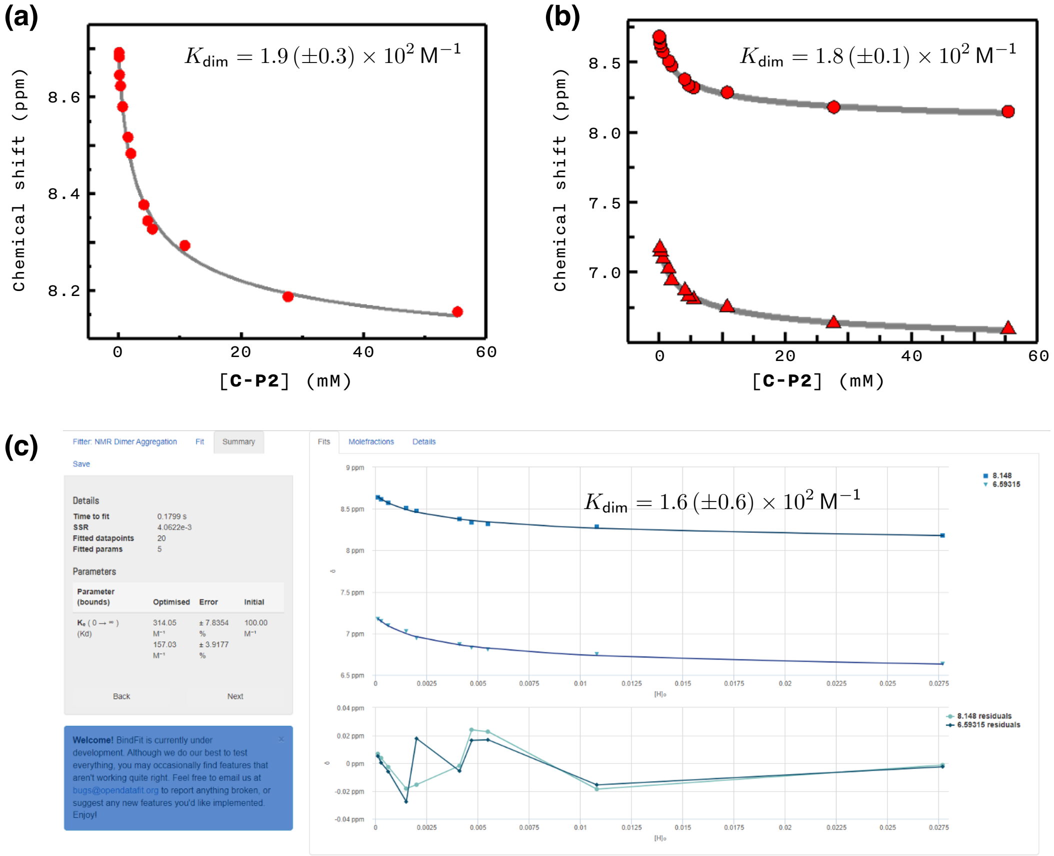

Supplementary Fig. 15. Changes in the chemical shifts of **C-P2** as a function of the concentration, modeled with (a) single-point fitting, (b) global fitting (by the graphing and data analysis software `Origin`), and (c) `Bindfit` (a web-based program). In each plot, the theoretical curve(s) with the dimerization constant K_{dim} are superimposed on the data points. Proton resonances are labeled with the symbols denoted in the chemical structure shown in Supplementary Fig. 12(c).

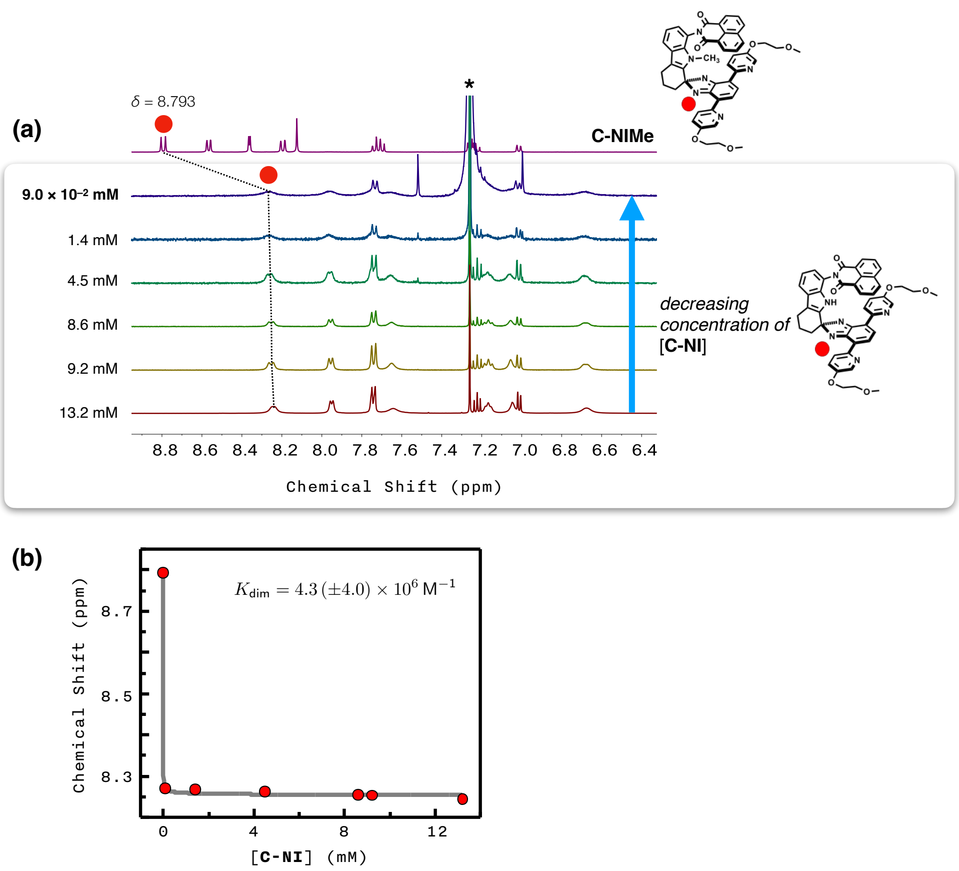

Supplementary Fig. 16. (a) Partial ^{1}H NMR spectra of **C-NI** in $CDCl_3$ (T = 298 K) obtained by varying the sample concentration, and compared with that of the **C-NIMe** at the top. Proton resonances are labeled with the symbols denoted in the chemical structures. The symbol * indicates residual solvent peak. (b) Changes in the chemical shift of the pyridine C–H proton resonance (red circles) as a function of sample concentration [**C-NI**]; the value at infinite dilution (i.e. monomer-only condition) was approximated with the corresponding signal of **C-NIMe** at δ = 8.793 ppm (see the spectrum in (a) above). The overlaid gray line is a theoretical fit with K_{dim} = 4.3 × 10^6 M^{-1}.

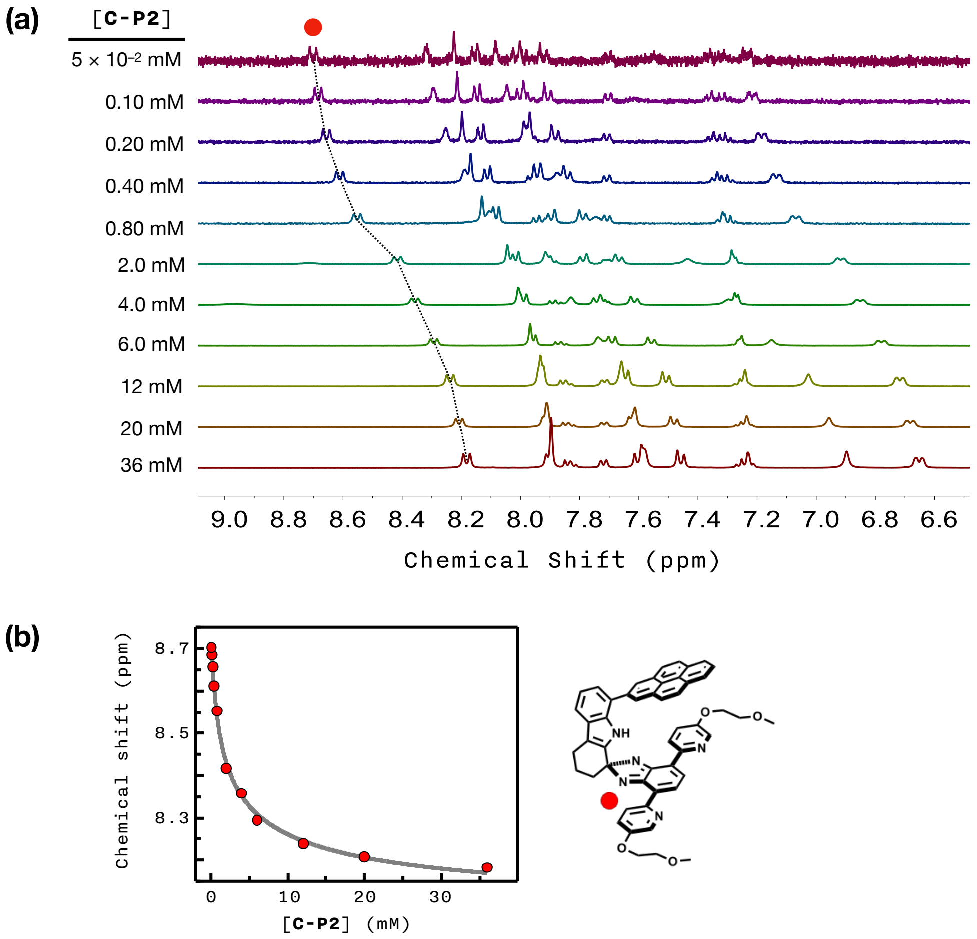

Supplementary Fig. 17. (a) Partial ^{1}H NMR spectra of **C-P2** in CD_2Cl_2 (T = 298 K) obtained by varying the sample concentration, with the pyridine C–H proton resonances labeled with a red circle in the chemical structure. (b) Concentration-dependent changes in the chemical shift of the pyridine C–H proton plotted using the data shown in (a). The overlaid gray line is a theoretical fit with $K_{\mathrm{dim}} = 3.4 \times 10^{2}$ M^{-1}.

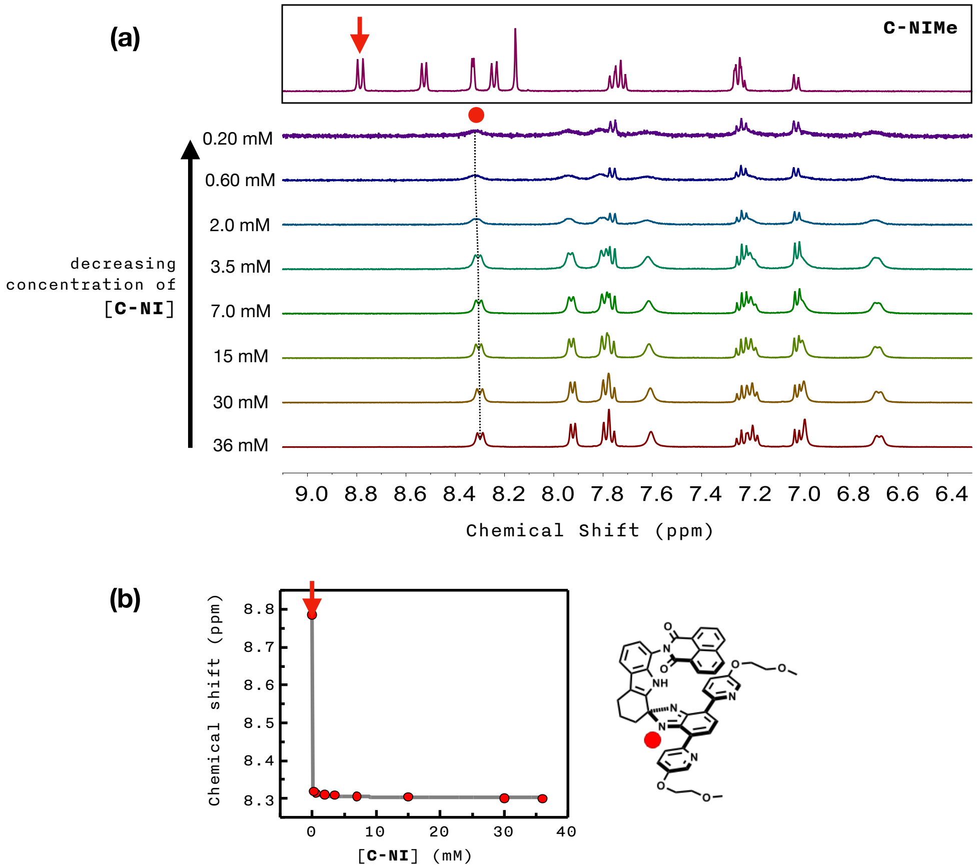

Supplementary Fig. 18. (a) Partial ^{1}H NMR spectra of **C-NI** in CD_2Cl_2 (T = 298 K) obtained by varying the sample concentration, and compared with that of the **C-NIMe** at the top. The pyridine C–H proton resonances of **C-NI** are labeled with a red circle in the chemical structure; the corresponding signal of **C-NIMe** with a red arrow. (b) Concentration-dependent changes in the chemical shift of the pyridine C–H proton plotted using the data shown in (a). Note that the value at infinite dilution (i.e. monomer-only condition) was approximated with the corresponding signal of **C-NIMe** at δ = 8.786 ppm (red arrow in (a)). The overlaid gray line is a theoretical fit with $K_{\mathrm{dim}} = 3.3 \times 10^5$ M^{-1}.

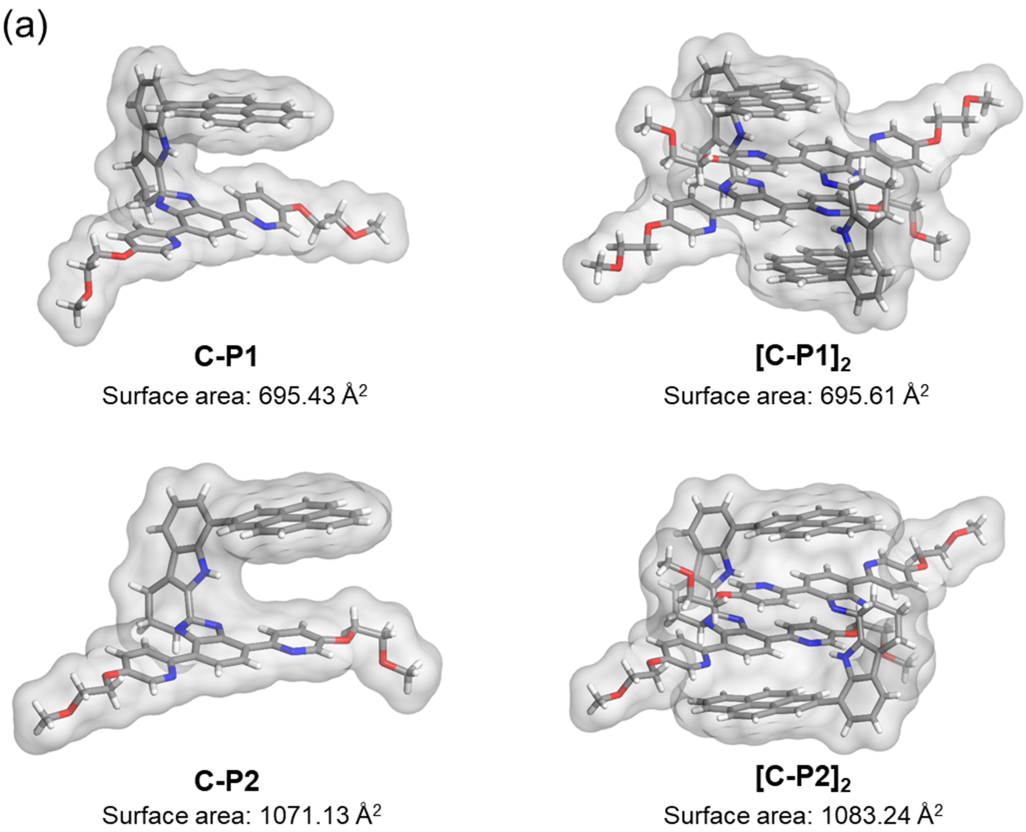

(b)

Probe Radius (Å)		**C** (Å^2)	[**C**]$_2$ (Å^2)	2**C** - [**C**]$_2$ (Å^2)
2.5	**C-P1**	700.01	1038.00	362.02
	**C-P2**	703.48	1051.10	355.86
1.4	**C-P1**	695.43	1071.13	319.73
	**C-P2**	695.61	1083.24	307.98
0.8	**C-P1**	703.72	1115.49	291.95
	**C-P2**	703.49	1145.76	261.22

Supplementary Fig. 19. (a) Connolly surfaces of the DFT models of monomeric **C-P1** and **C-P2** vs crystallographically determined structures of dimeric [**C-P1**]$_2$ and [**C-P2**]$_2$ analyzed with a probe radius of 1.4 Å. (b) Comparison of the Connolly surfaces between the monomer and dimer analyzed with three different probe radii.

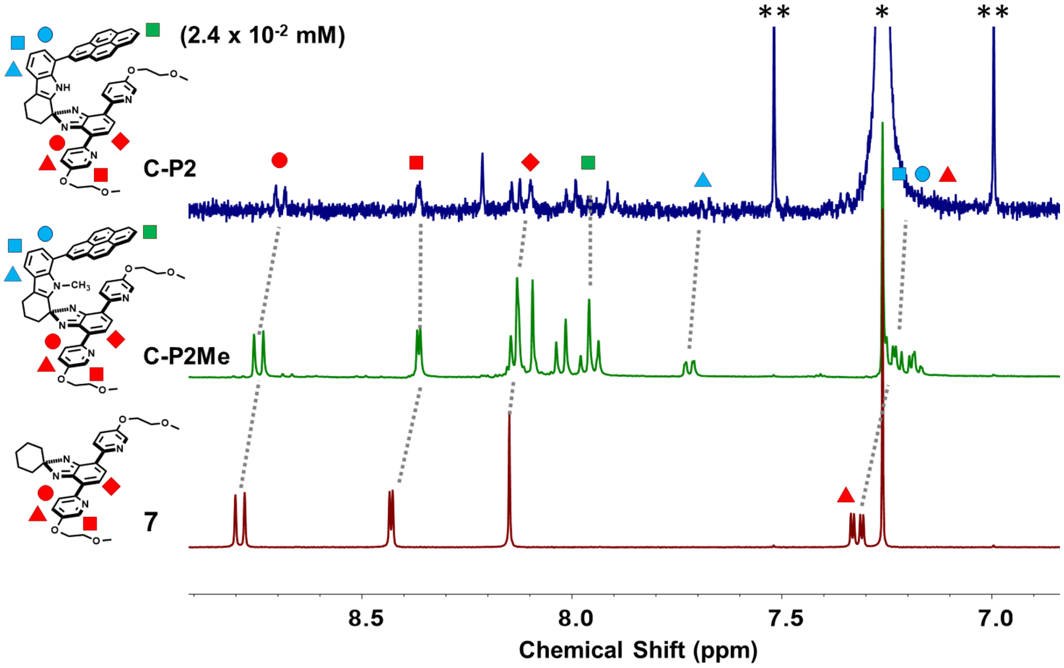

Supplementary Fig. 20. Aromatic region in the ^{1}H NMR spectra of **C-P2** (2.4 × 10^{-2} mM), **C-P2Me**, and **7** in $CDCl_3$ at T = 298 K.

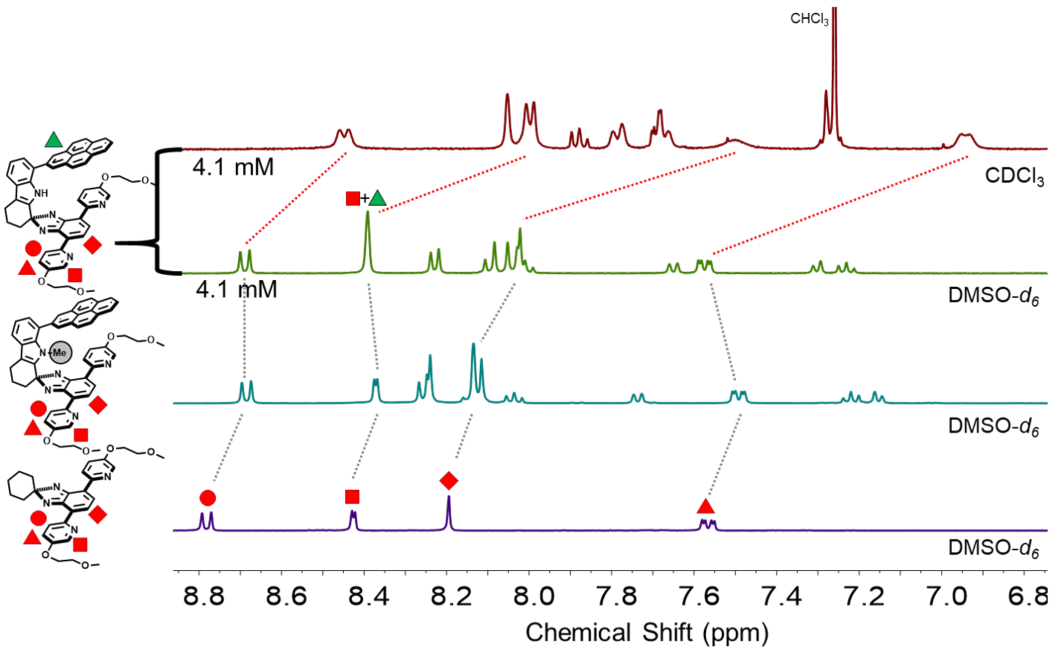

Supplementary Fig. 21. Aromatic region in the ^{1}H NMR spectra of **C-P2** (4.1 mM) in $CDCl_3$, and **C-P2** (4.1 mM), **C-P2Me**, and **7** in DMSO-d_6 at T = 298 K.

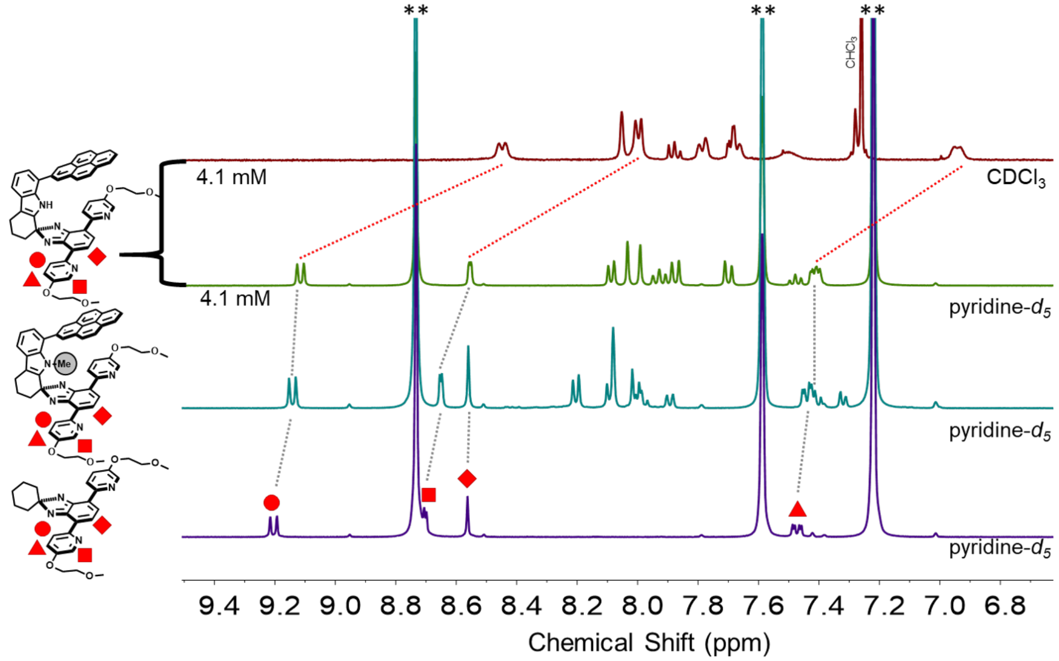

Supplementary Fig. 22. Aromatic region in the ^{1}H NMR spectra of **C-P2** (4.1 mM) in $CDCl_3$, and **C-P2** (4.1 mM), **C-P2Me**, and **7** in pyridine-d_5 at T = 298 K. Asterisks (*) denote residual solvent peaks.

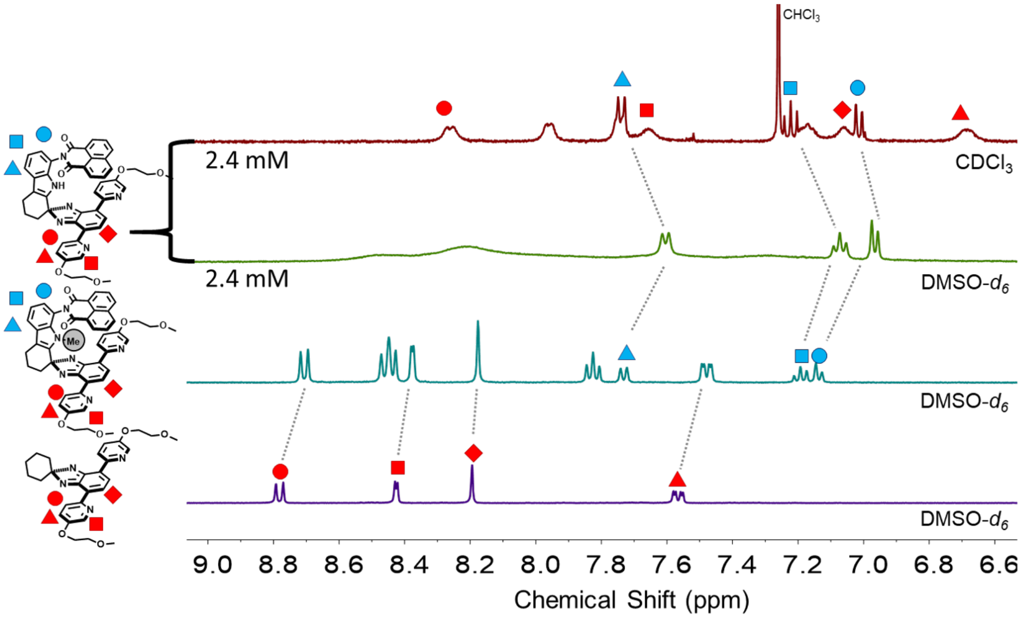

Supplementary Fig. 23. Aromatic region in the ^{1}H NMR spectra of **C-NI** (2.4 mM) in $CDCl_3$, and **C-NI** (2.4 mM), **C-NIMe**, and **7** in DMSO-d_6 at T = 298 K.

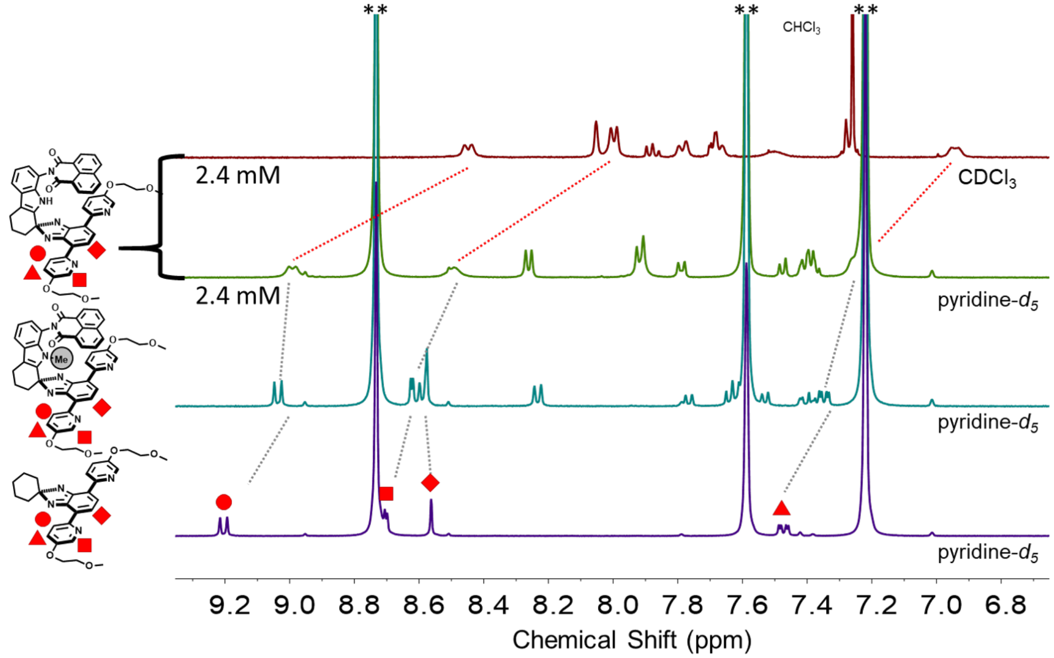

Supplementary Fig. 24. Aromatic region in the ^{1}H NMR spectra of **C-NI** (2.4 mM) in $CDCl_3$, and **C-NI** (2.4 mM), **C-NIMe**, and **7** in pyridine-d_5 at T = 298 K. Asterisks (*) denote residual solvent peaks.

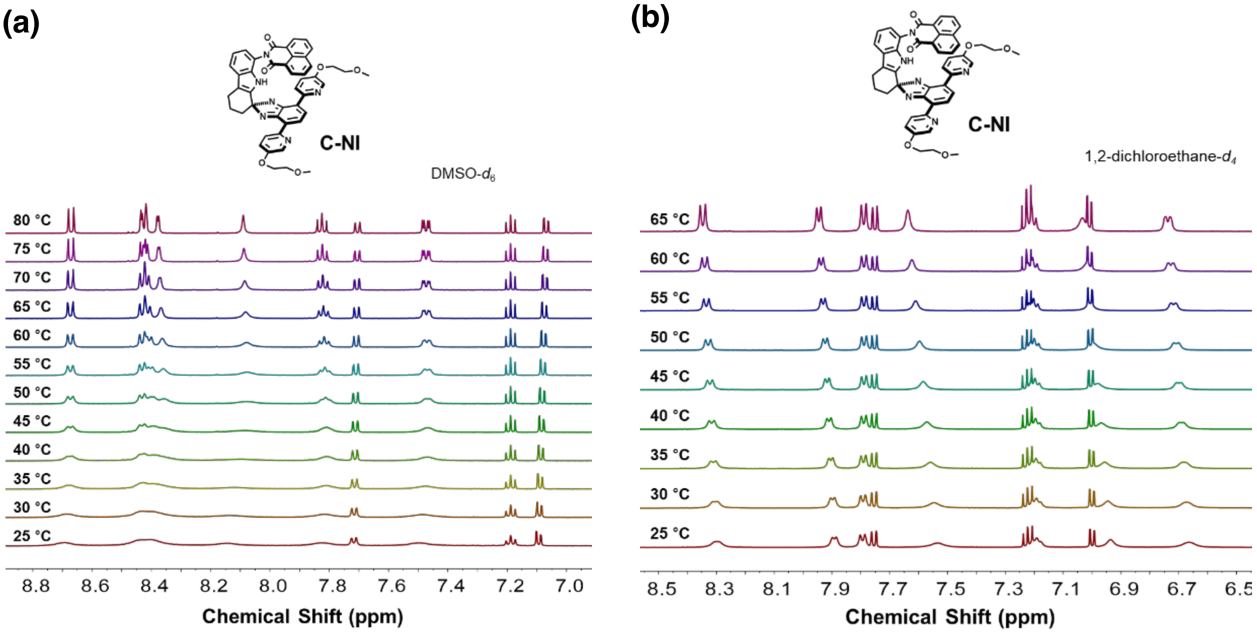

Supplementary Fig. 25. Variable-temperature (VT) ^{1}H NMR spectra of **C-NI** (3.5 mM) in (a) DMSO-d_6 and (b) 1,2-dichloroethane-d_4.

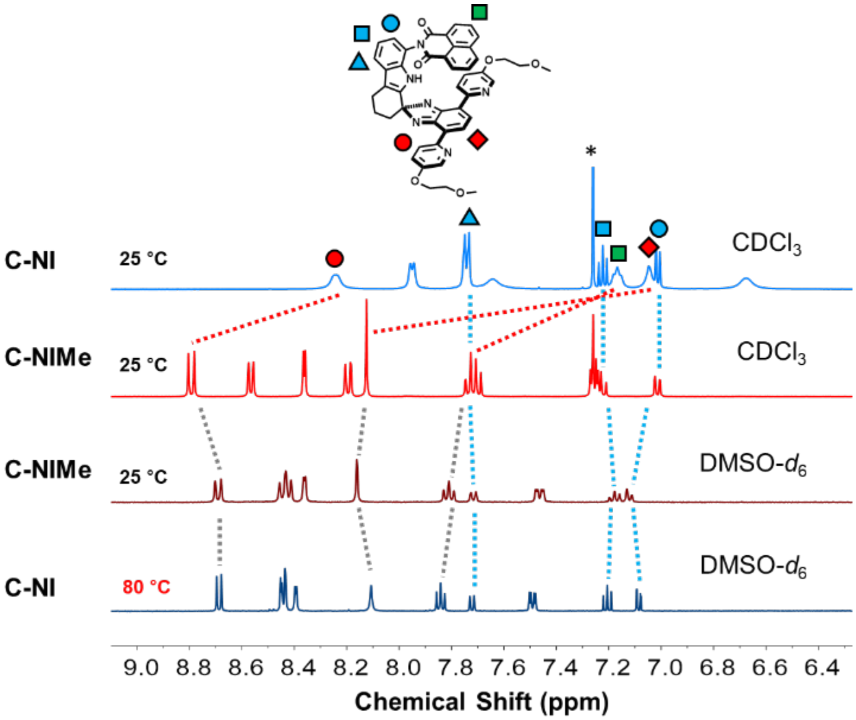

Supplementary Fig. 26. Partial ^{1}H NMR spectra of **C-NI** and **C-NIMe** in the aromatic region. The symbols * denote residual solvent peaks.

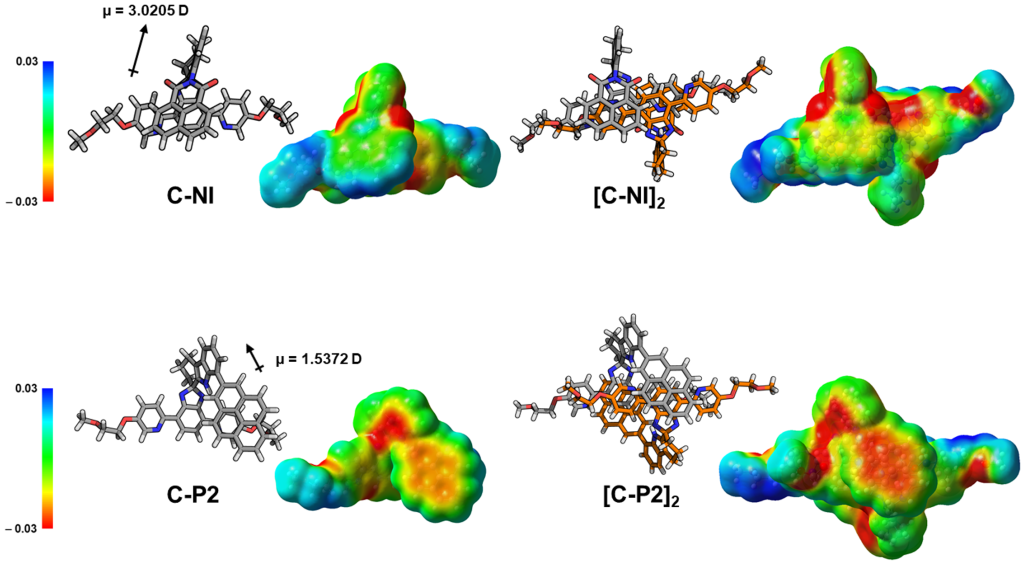

Supplementary Fig. 27. Molecular electronic potential (MEP) maps and dipole moments of **C-NI**, [**C-NI**]$_2$, **C-P2**, and [**C-P2**]$_2$ calculated at the B3LYP-D3/6-31G(d,p) level of theory. For [**C-NI**]$_2$ and [**C-P2**]$_2$, crystallographically determined atomic coordinates were used as an input.

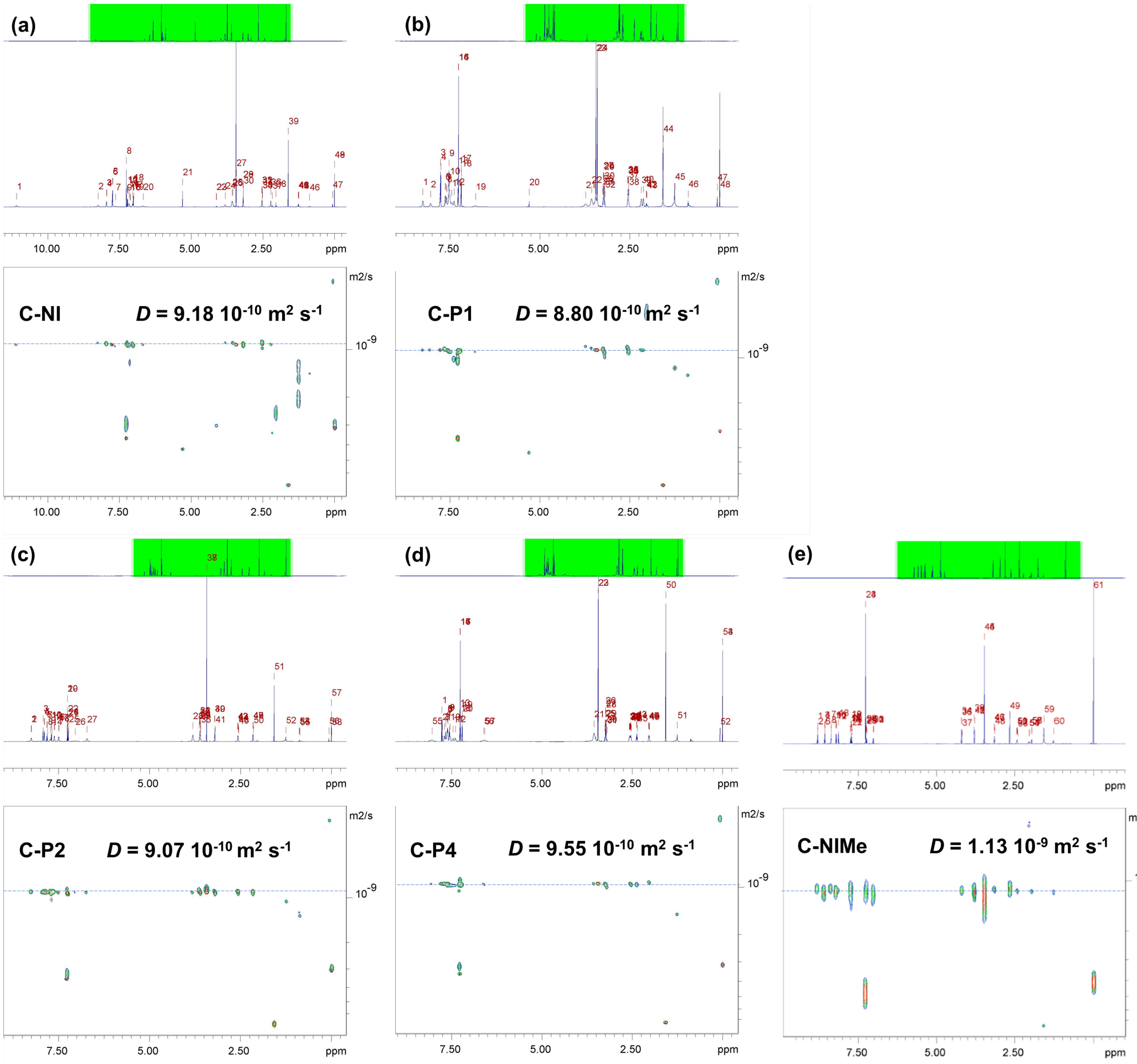

Supplementary Fig. 28. ^{1}H DOSY spectra (850 MHz) of **C-NI**, **C-P1**, **C-P2**, **C-P4**, and **C-NIMe** in $CDCl_3$ (sample concentrations = 10 mM) at T = 298 K.

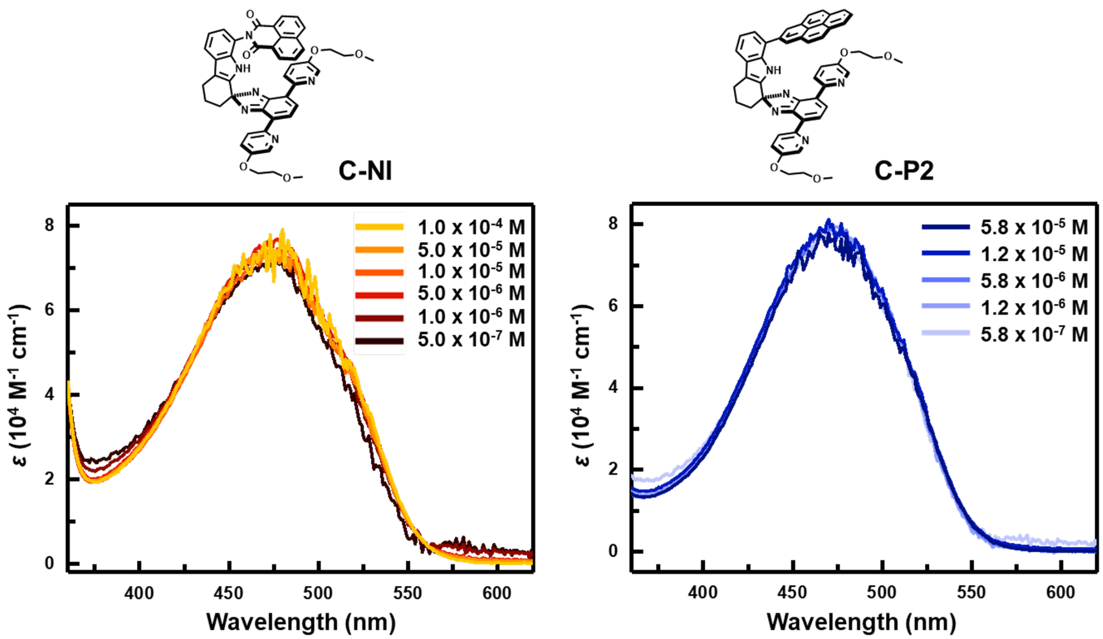

Supplementary Fig. 29. Concentration dependent UV–vis spectra of **C-NI** and **C-P2** in $CHCl_3$ at $T = 298$ K.

Supplementary Note 2. Determining the Host–Guest Complexation Constant (K_{HG})
^{1}H NMR titration studies were carried out in $CDCl_3$ at T = 298 K (Supplementary Figs. 20 and 21). The concentration of the guest PHD was kept at 0.45 mM. Aliquots of the host molecule were delivered from the stock solution (50 mM for (**C-P2**) and 15 mM for (**C-NI**)). The NMR tube was sonicated to ensure homogenous mixing. Spectra were taken after the temperature was stabilized at T = 298 K.

The host **C-P2** (= H) can either (i) self-associate to form $\mathsf{H_2}$, or (ii) encapsulate the gust (= G) to afford HG.

$$\mathsf{H} + \mathsf{H} \xrightleftharpoons{K_{\mathsf{dim}}} \mathsf{H_2}$$

$$\mathsf{H} + \mathsf{G} \xrightleftharpoons{K_{\mathsf{HG}}} \mathsf{HG}$$

$$K_{\mathsf{dim}} = \frac{[\mathsf{H_2}]}{[\mathsf{H}]^2} \tag{2}$$

$$K_{\mathsf{HG}} = \frac{[\mathsf{HG}]}{[\mathsf{H}][\mathsf{G}]} \tag{3}$$

$$[\mathsf{H}]_0 = [\mathsf{H}] + [\mathsf{HG}] + 2[\mathsf{H_2}] \tag{4}$$

The following equation is obtained from eqs (2) and (4):

$$[\mathsf{H}] = \frac{\sqrt{1 - 8K_{\mathsf{dim}}([\mathsf{HG}] - [\mathsf{H}]_0)} - 1}{4K_{\mathsf{dim}}} \tag{5}$$

By combining eqs (3) and (5), we obtain

$$K_{\mathsf{HG}} = \frac{4K_{\mathsf{dim}}[\mathsf{HG}]}{(\sqrt{1 - 8K_{\mathsf{dim}}([\mathsf{HG}] - [\mathsf{H}]_0)} - 1)[\mathsf{G}]} \tag{6}$$

For the host G,

$$[\mathsf{G}]_0 = [\mathsf{G}] + [\mathsf{HG}] \tag{7}$$

$$[\mathsf{G}] = \left(\frac{\delta^{\mathsf{G}}_{obs} - \delta^{\mathsf{G}}_{\mathsf{HG}}}{\delta^{\mathsf{G}} - \delta^{\mathsf{G}}_{\mathsf{HG}}}\right)[\mathsf{G}]_0 \tag{8}$$

$$[\mathsf{HG}] = \left(1 - \frac{\delta^{\mathsf{G}}_{obs} - \delta^{\mathsf{G}}_{\mathsf{HG}}}{\delta^{\mathsf{G}} - \delta^{\mathsf{G}}_{\mathsf{HG}}}\right)[\mathsf{G}]_0 \tag{9}$$

, where $\delta^{\mathsf{G}}_{obs}$, $\delta^{\mathsf{G}}_{\mathsf{HG}}$, and δ^{G} denote the observed chemical shift, chemical shift of the host–guest complex, and chemical shift of the unbound guest, respectively.

From eqs (6), (8), and (9), the following relationship is established:

$$[\mathsf{H}]_0 = \frac{\left\{1 + \left(4K_{\mathsf{dim}}\dfrac{1-\frac{\delta^{\mathsf{G}}_{obs}-\delta^{\mathsf{G}}_{\mathsf{HG}}}{\delta^{\mathsf{G}}-\delta^{\mathsf{G}}_{\mathsf{HG}}}}{K_{\mathsf{HG}}\frac{\delta^{\mathsf{G}}_{obs}-\delta^{\mathsf{G}}_{\mathsf{HG}}}{\delta^{\mathsf{G}}-\delta^{\mathsf{G}}_{\mathsf{HG}}}}\right)\right\}^2 + 8K_{\mathsf{dim}}\left(1-\frac{\delta^{\mathsf{G}}_{obs}-\delta^{\mathsf{G}}_{\mathsf{HG}}}{\delta^{\mathsf{G}}-\delta^{\mathsf{G}}_{\mathsf{HG}}}\right)[\mathsf{G}]_0 - 1}{8K_{\mathsf{dim}}} \quad (10)$$

Using eq (10), numerical global fittings were carried out by non-linear least-squares regression analysis to obtain K_{HG}.

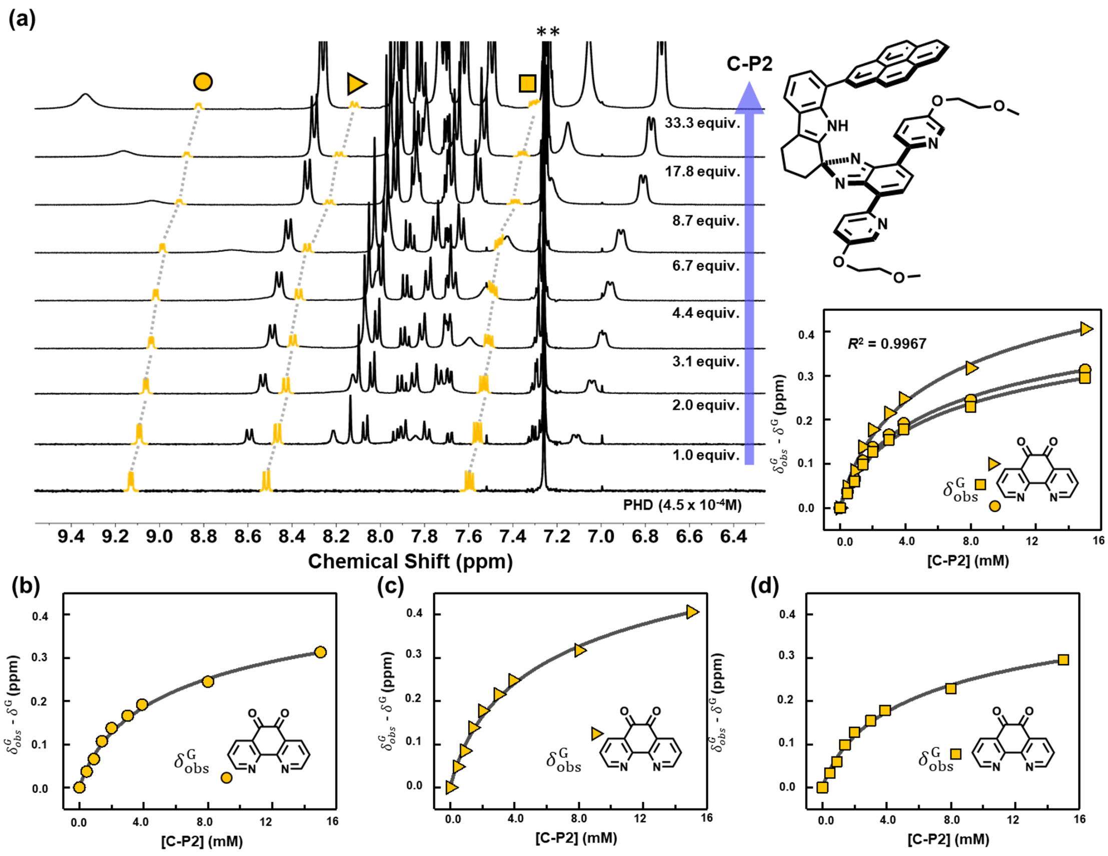

Supplementary Fig. 30. (a) Partial ^{1}H NMR spectra of PHD in $CDCl_3$ obtained in the presence of **C-P2** at T = 298 K. Chemical resonances of PHD are shaded with yellow. The symbols ** denotes residual solvent. Changes in the chemical shifts of the H_{PHD} proton resonances as a function of the concentration [**C-P2**]. The theoretical curve with K_{HG} = 2.0 × 10^2 M^{-1} is superimposed on the data points. In (b)–(d), changes in the chemical shifts of the PHD proton resonance (b) ● , (c) ▼ , and (d) ■, as a function of [**C-P2**], are provided individually for clarity.

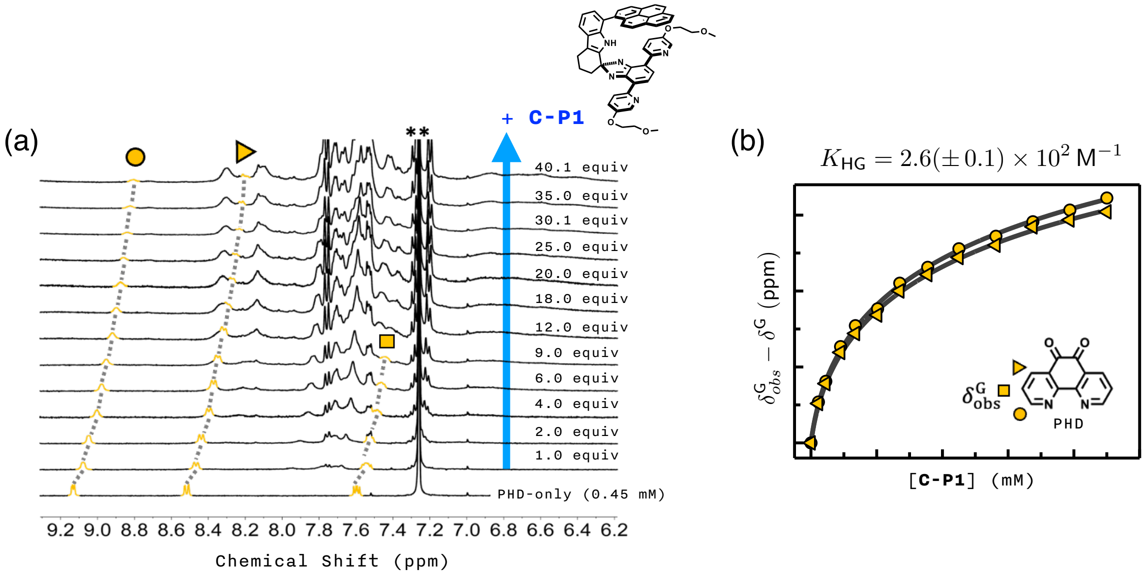

Supplementary Fig. 31. (a) Partial ^{1}H NMR spectra of PHD in $CDCl_3$ obtained in the presence of **C-P1** at T = 298 K. Chemical resonances of PHD are shaded with yellow. The symbols ** denotes residual solvent peaks. (b) Changes in the chemical shifts of the proton resonances as a function of the concentration [**C-P1**]. The theoretical curve with $K_{HG} = 2.6 \times 10^2$ M^{-1} is overlaid with the data points.

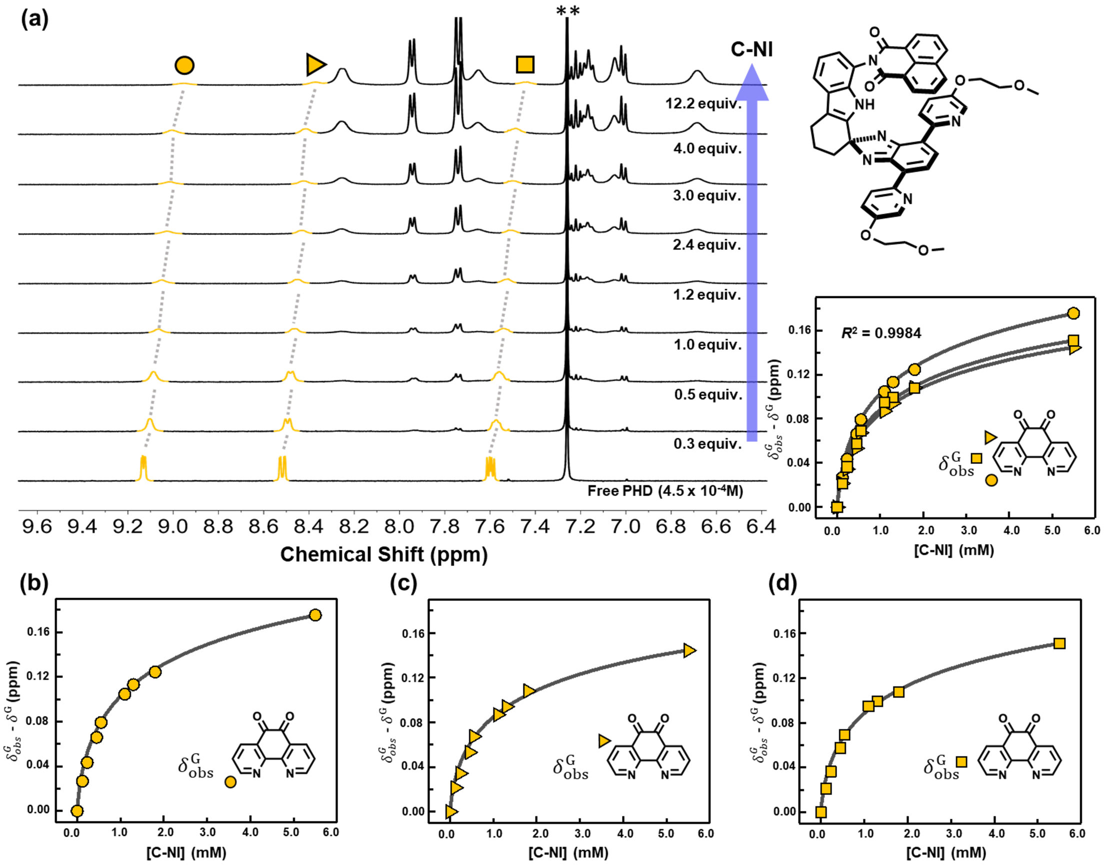

Supplementary Fig. 32. (a) Partial ^{1}H NMR spectra of PHD in $CDCl_3$ obtained in the presence of **C-NI** at $T = 298$ K. Chemical resonances of PHD are shaded with yellow. The symbols ** denotes residual solvent. Changes in the chemical shifts of the H_{PHD} proton resonances as a function of the concentration [**C-NI**]. The theoretical curve with $K_{HG} = 8.3 \times 10^3$ M^{-1} is superimposed on the data points. In (b)–(d), changes in the chemical shifts of the PHD proton resonance (b) ● , (c) ▼ , and (d) ■, as a function of [**C-NI**], are provided individually for clarity.

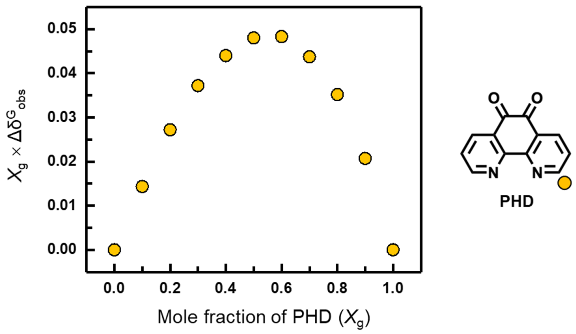

Supplementary Fig. 33. A Job plot to determine the stoichiometry of the complexation between **C-P2** and PHD using shifts in the proton resonance of PHD (●) ([**C-P2**] + [PHD] = 3.1 mM in $CDCl_3$ at T = 298 K).

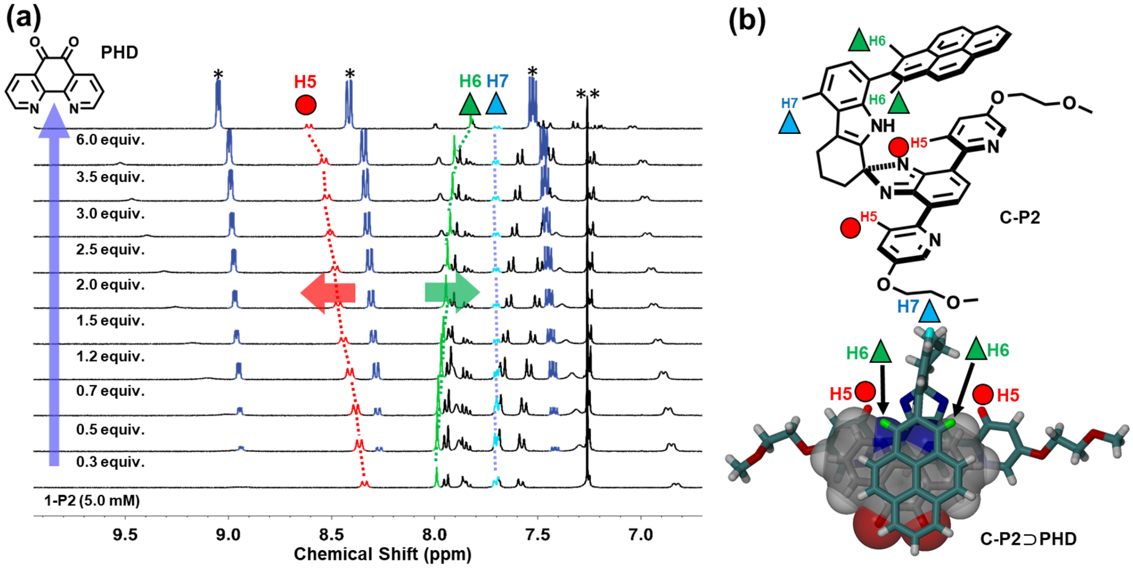

Supplementary Fig. 34. (a) Partial ^{1}H NMR spectra of **C-P2** (5.0 mM) obtained in the presence of increasing equiv of PHD (denoted with *) in $CDCl_3$ at T = 298 K. The symbol ** denotes residual solvent peaks. The pyridine (H5), pyrene (H6), and indole (H7) proton resonances are labeled according to the chemical structure and X-ray structure shown in (b).

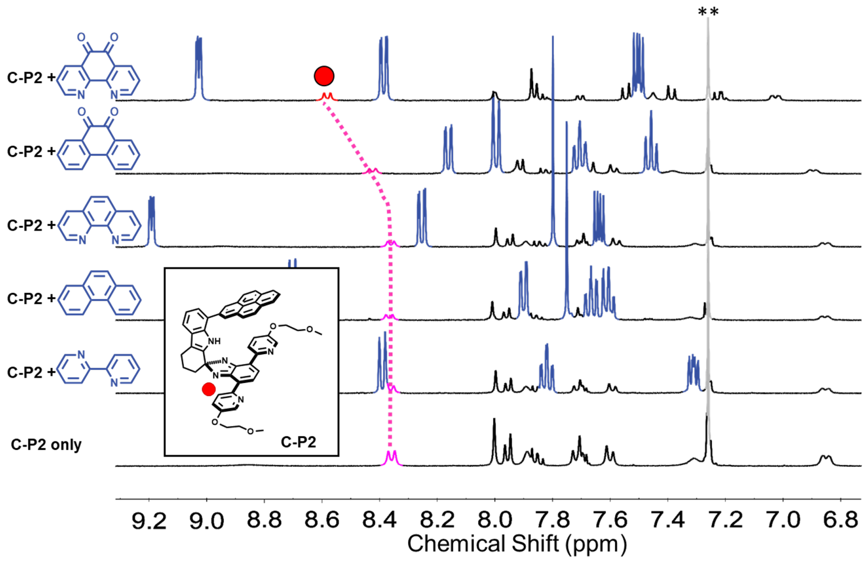

Supplementary Fig. 35. (a) Aromatic region in the ^{1}H NMR spectra of **C-P2** obtained in the presence of various poly(hetero)aromatic guests (G) in $CDCl_3$ at T = 298 K ([**C-P2**] = 4.0 mM; [G] = 16.0 mM). The pyridyl proton (denoted with red circle in the chemical structure of **C-P2** at the bottom) resonances are colored in purple to red. The proton resonances of the guests are in blue. The symbol ** denotes residual solvent.

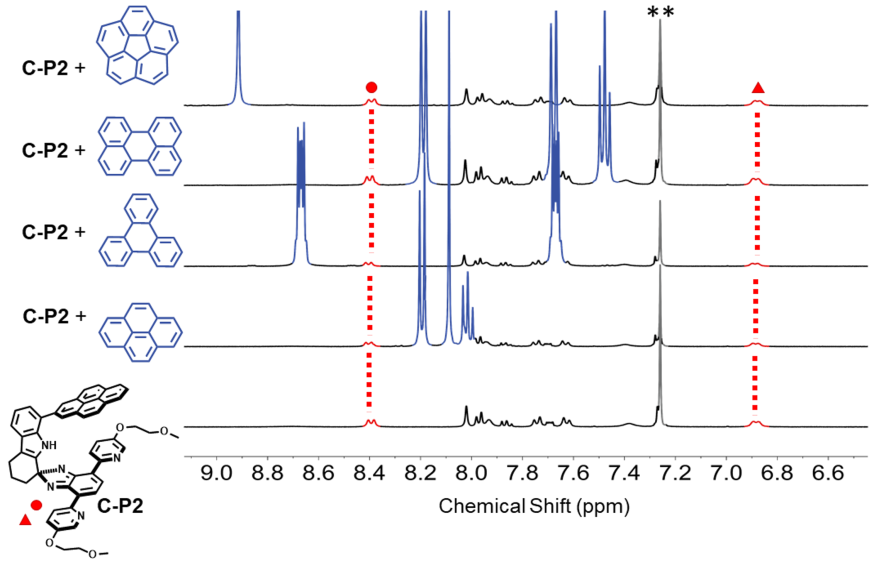

Supplementary Fig. 36. (a) Aromatic region in the ^{1}H NMR spectra of **C-P2** obtained in the presence of various polyaromatic hydrocarbon guests (G) in $CDCl_3$ at T = 298 K ([**C-P2**] = 4.0 mM; [G] = 16.0 mM, except for corannulene (4.0 mM) and perylene (3.0 mM) due to low solubility). The pyridyl protons (denoted with red circle and red trianlge in the chemical structure of **C-P2** at the bottom left) are in red. The proton resonances of the guests are in blue. The symbol ** denotes residual solvent.

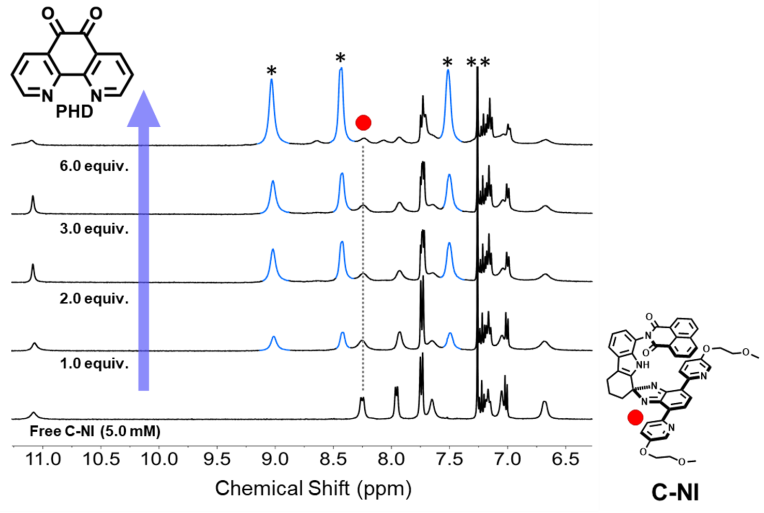

Supplementary Fig. 37. (a) Partial ^{1}H NMR spectra of **C-NI** (5.0 mM) in $CDCl_3$ obtained in the presence of PHD at T = 298 K. The symbols * and ** denote PHD and residual solvent peaks, respectively.

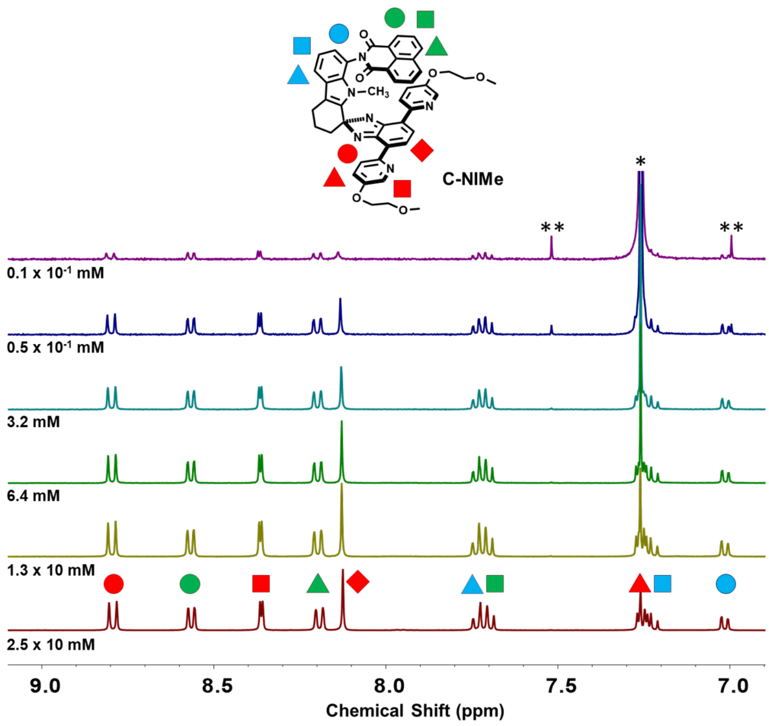

Supplementary Fig. 38. Partial ^{1}H NMR spectra of **C-NIMe** at different concentrations in $CDCl_3$ measured at T = 298 K. The symbols * and ** denote residual solvent and satellite peaks, respectively.

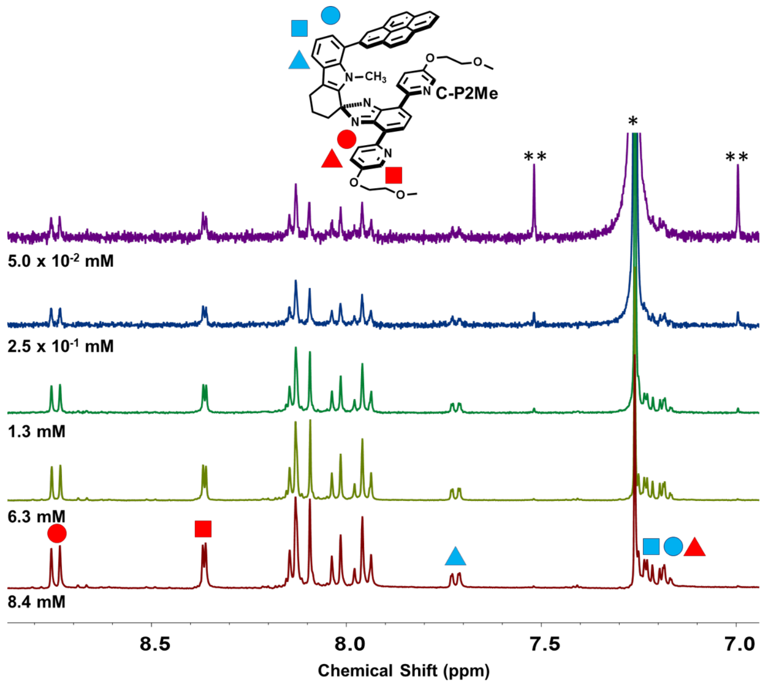

Supplementary Fig. 39. Partial ^{1}H NMR spectra of **C-P2Me** at different concentrations in $CDCl_3$ measured at T = 298 K. The symbols * and ** denote residual solvent and satellite peaks, respectively.

Supplementary Note 3. Numerical Simulations of the Speciation Plot

The speciation plots in Fig. 10 were numerically simulated by using the experimentally determined dimerization constant ($K_{\mathsf{dim}} = 1.3 \times 10^5$ M^{-1}), host–guest complexation constant ($K_{\mathsf{HG}} = 8.3 \times 10^3$ M^{-1}), and a fixed total concentration of [**C-NI**]$_0$ = 5.0 mM. The y-axis reports changes in the mole fractions of the free (= unbound) host **C-NI**, dimerized host [**C-NI**]$_2$, and host-guest complex **C-NI**⊃PHD as a function of the total guest concentration [PHD]$_0$ corresponding to the x-axis.

From eqs (2), (3), (4), and (7) in **Supplementary Note 2**, the following relationship is established:

$$[\mathsf{H}] + 2K_{\mathsf{dim}}[\mathsf{H}]^2 + \left(\frac{[\mathsf{H}][\mathsf{G}]_0}{[\mathsf{H}] + \dfrac{1}{K_{\mathsf{HG}}}} \right) = [\mathsf{H}]_0$$

Using the K_{dim}, K_{HG}, and $[\mathsf{H}]_0$ (= [**C-NI**]$_0$) values as constants (see above), the concentration of the unbound host H (= [**C-NI**]) is calculated by varying the $[\mathsf{G}]_0$ = ([PHD]$_0$) value. The speciation plot (blue line in Fig. 10) is drawn for the change in the mole fraction [**C-NI**]/[**C-NI**]$_0$ (= y-axis) as a function of [PHD]$_0$ (= x-axis; as equiv amounts to [**C-NI**]$_0$).

From eqs (2), (3), (4), and (7) in **Supplementary Note 2**, the following relationship is established:

$$\sqrt{\frac{[\mathsf{H}_2]}{K_{\mathsf{dim}}}} + 2[\mathsf{H}_2] + [\mathsf{G}]_0 - \left(\frac{[\mathsf{G}]_0}{K_{\mathsf{HG}} \cdot \sqrt{\dfrac{[\mathsf{H}_2]}{K_{\mathsf{dim}}}} + 1} \right) = [\mathsf{H}]_0$$

Using the K_{dim}, K_{HG}, and $[\mathsf{H}]_0$ (= [**C-NI**]$_0$) values as constants (see above), the concentration of the dimeric H_2 (= [**C-NI**]$_2$) is calculated by varying the $[\mathsf{G}]_0$ = ([PHD]$_0$) value. The speciation plot (red line in Fig. 10) is drawn for the change in the mole fraction [**C-NI**]$_2$/[**C-NI**]$_0$ (= y-axis) as a function of [PHD]$_0$ (= x-axis; as equiv amounts to [**C-NI**]$_0$).

From eqs (3), (5), and (7) in **Supplementary Note 2**, the following relationship is established:

$$K_{\mathsf{HG}} \left(\sqrt{1 - 8K_{\mathsf{dim}}([\mathsf{HG}] - [\mathsf{H}]_0)} - 1 \right) ([\mathsf{G}]_0 - [\mathsf{HG}]) = 4K_{\mathsf{dim}}[\mathsf{HG}]$$

Using the K_{dim}, K_{HG}, and $[\mathsf{H}]_0$ (= [**C-NI**]$_0$) values as constants (see above), the concentration of the host–guest complex HG (= [**C-NI**⊃PHD]) is calculated by varying the $[\mathsf{G}]_0$ = ([PHD]$_0$) value. The speciation plot (green line in Fig. 10) is drawn for the change in the mole fraction [**C-NI**⊃PHD]/[**C-NI**]$_0$ (= y-axis) as a function of [PHD]$_0$ (= x-axis; as equiv amounts to [**C-NI**]$_0$).

Supplementary References

1. Heiskanen, J. P. et al. Synthesis of Benzothiadiazole Derivatives by Applying C–C Cross-Couplings. *J. Org. Chem.* **81**, 1535–1546 (2016).

2. Wang, H., Zhou, G., Gai, H. & Chen, X. A Fluorescein-Based Probe with High Selectivity to Cysteine over Homocysteine and Glutathione. *Chem. Commun.* **48**, 8341–8343 (2012).

3. Dziedzic, P. et al. Design, Synthesis, and Protein Crystallography of Biaryltriazoles as Potent Tautomerase Inhibitors of Macrophage Migration Inhibitory Factor. *J. Am. Chem. Soc.* **137**, 2996–3003 (2015).

4. Su, L. et al. Design, Synthesis and Evaluation of Hybrid of Tetrahydrocarbazole with 2,4-Diaminopyrimidine Scaffold as Antibacterial Agents. *Eur. J. Med. Chem.* **162**, 203–211 (2019).

5. Shi, H., Guo, T., Zhang-Negrerie, D., Du, Y. & Zhao, K. Synthesis of Substituted Tetrahydron-1*H*-carbazol-1-one and Analogs via $PhI(OCOCF_3)_2$-Mediated Oxidative C–C Bond Formation. *Tetrahedron* **70**, 2753–2760 (2014).

6. Oniwa, K. et al. 2-Positional Pyrene End-Capped Oligothiophenes for High Performance Organic Field Effect Transistors. *Chem. Commun.* **52**, 4800–4803 (2016).

7. Lorbach, D., Wagner, M., Baumgarten, M. & Müllen, K. The Right Way to Self-Buse Bi- and Terpyrenyls to Afford Graphenic Cutouts. *Chem. Commun.* **49**, 10578–10580 (2013).

8. Thakur, K. G., Srinivas, K. S., Chiranjeevi, K. & Sekar, G. D-Glucosamine as an Efficient Ligand for the Copper-Catalyzed Selective Synthesis of Anilines from Aryl Halides and NaN_3. *Green Chem.* **13**, 2326–2329 (2011).

9. Sheldrick, G. M. *SHELXT* – Integrated Space-Group and Crystal-Structure Determination. *Acta Cryst.* **A71**, 3–8 (2015).

10. Sheldrick, G. M. Crystal Structure Refinement with SHELXL. *Acta Cryst.* **C71**, 3–8 (2015).

11. Dolomanov, O. V., Bourhis, L. J., Gildea, R. J., Howard, J. A. K. & Puschmann, H. *OLEX2*: A Complete Structure Solution, Refinment and Analysis Program.*J. Appl. Cryst.* **42**, 339–341 (2009).

12. Shin, J. W., Eom, K. & Moon, D. BL2D-SMC, The Supramolecular Crystallography Beamline at the Pohang Light Source II, Korea. *J. Synchrotron Rad.* **23**, 369–373 (2016).

13. Otwinowski, Z. & Minor, W. Processing of X-ray Diffraction Data Collected in Oscillation Mode. *Methods Enzymol.* **276**, 307–326 (1997).
