# Supplementary material for: Molecular Tetris by sequence-specific stacking of hydrogen bonding molecular clips
Source: Commun Chem. 2022 Dec 28;5:180. doi: 10.1038/s42004-022-00802-4 (PMC9814962; doi:10.1038/s42004-022-00802-4)
Supplement: Supplementary file 3 — Description of Additional Supplementary Files [file 42004_2022_802_MOESM3_ESM.pdf]

Description of Additional Supplementary Files

File name: Supplementary Data 1

Description: NMR spectra

File name: Supplementary Data 2

Description: DFT Cartesian coordinates

File name: Supplementary Movie

Description: A schematic animation of self-assembly and fluxional motion
