# Supplementary material for: Molecular Tetris by sequence-specific stacking of hydrogen bonding molecular clips
Source: Commun Chem. 2022 Dec 28;5:180. doi: 10.1038/s42004-022-00802-4 (PMC9814962; doi:10.1038/s42004-022-00802-4)
Supplement: Supplementary file 4 — Supplementary Data 1 [file 42004_2022_802_MOESM4_ESM.pdf]

Supplementary Data 1

Molecular Tetris by sequence-specific stacking of hydrogen bonding molecular clips

Hyun Lee and Dongwhan Lee*

Department of Chemistry, Seoul National University, 1 Gwanak-ro, Gwanak-gu, Seoul 08826, Korea

NMR spectra

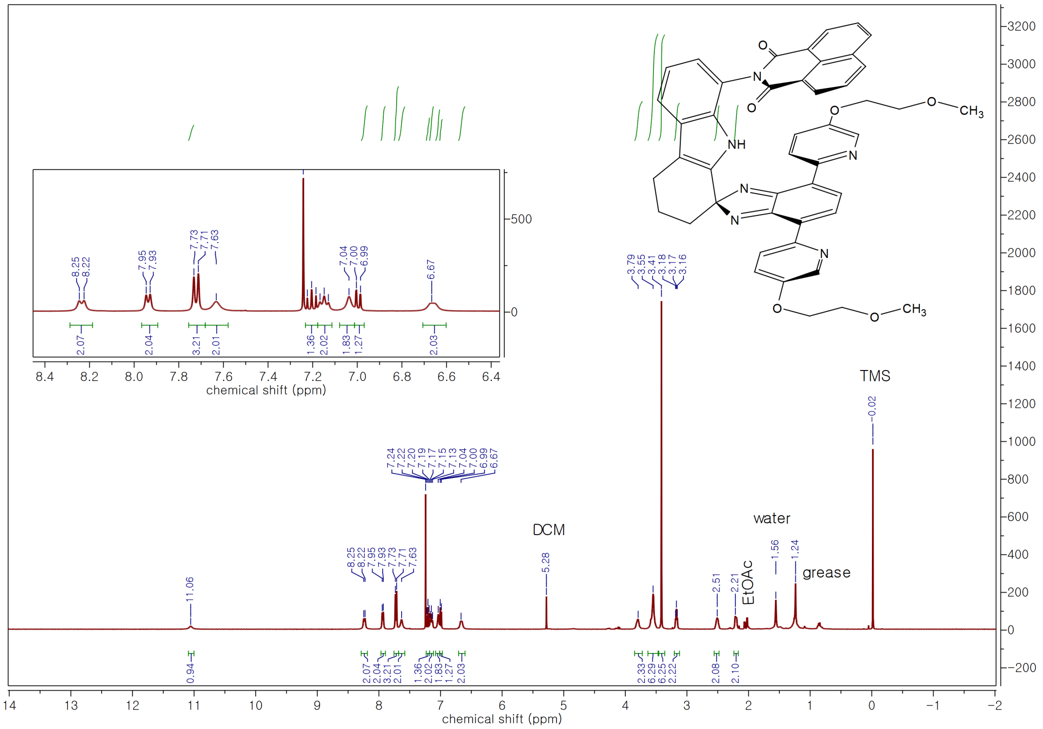

^{1}H NMR (400 MHz) spectrum of **C-NI** in $CDCl_3$ (T = 298 K).

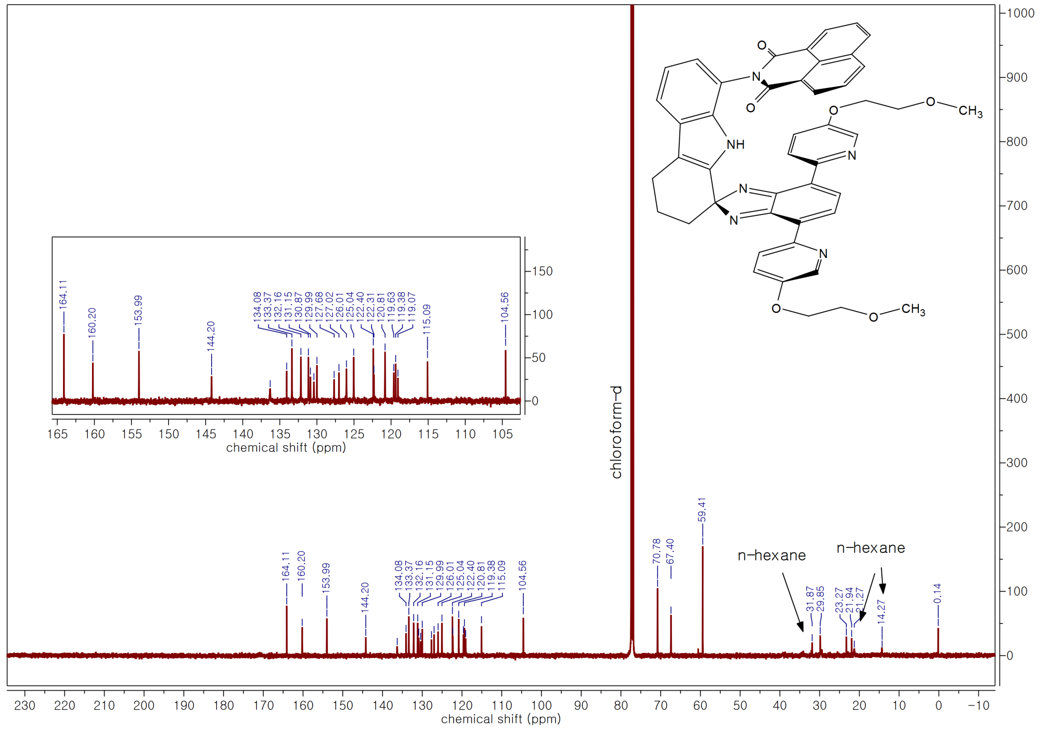

^{13}C NMR (125 MHz) spectrum of **C-NI** in $CDCl_3$ (T = 298 K).

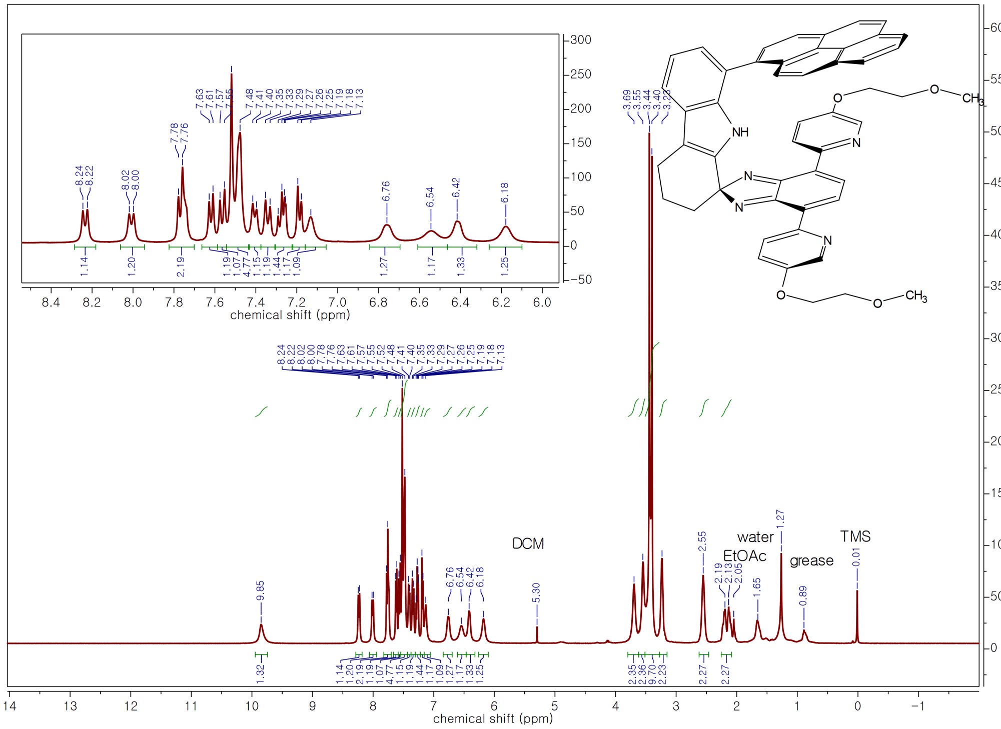

^{1}H NMR (400 MHz) spectrum of **C-P1** in $CDCl_3$ (T = 298 K).

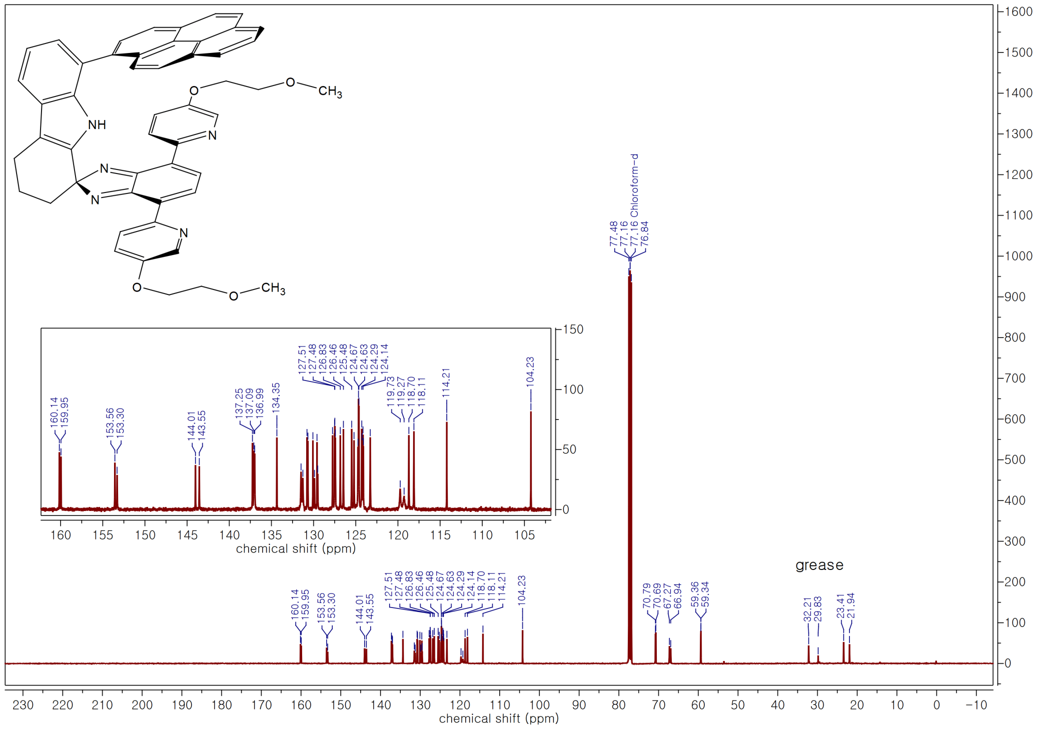

^{13}C NMR (100 MHz) spectrum of **C-P1** in $CDCl_3$ (T = 298 K).

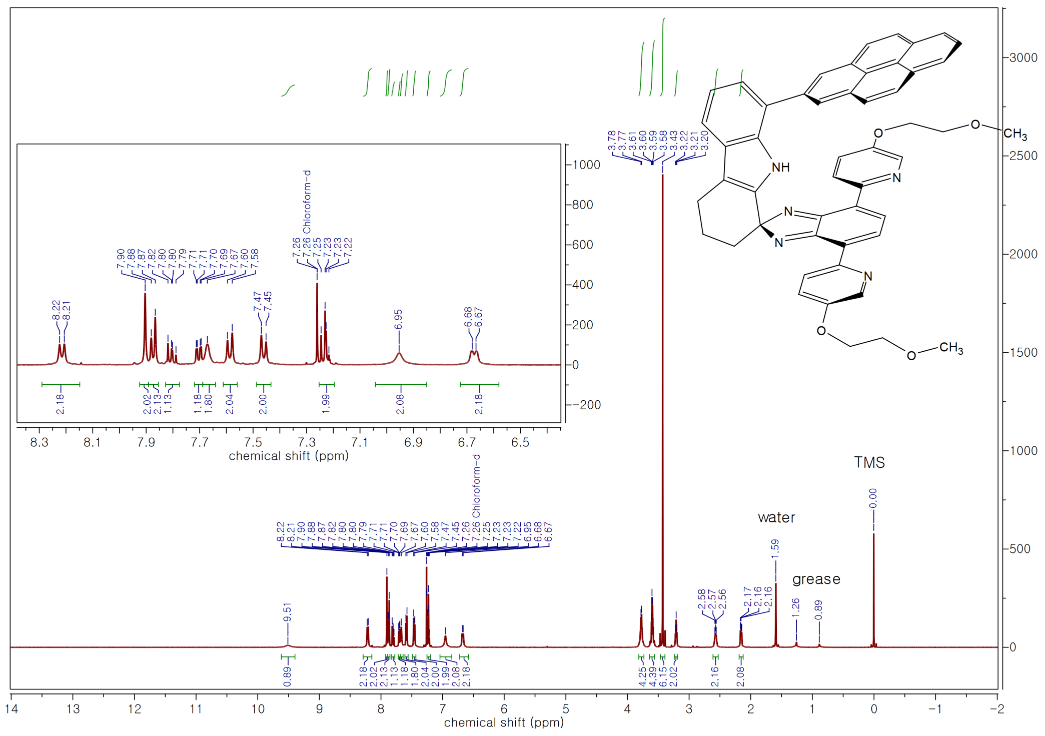

^{1}H NMR (500 MHz) spectrum of **C-P2** in $CDCl_3$ (T = 298 K).

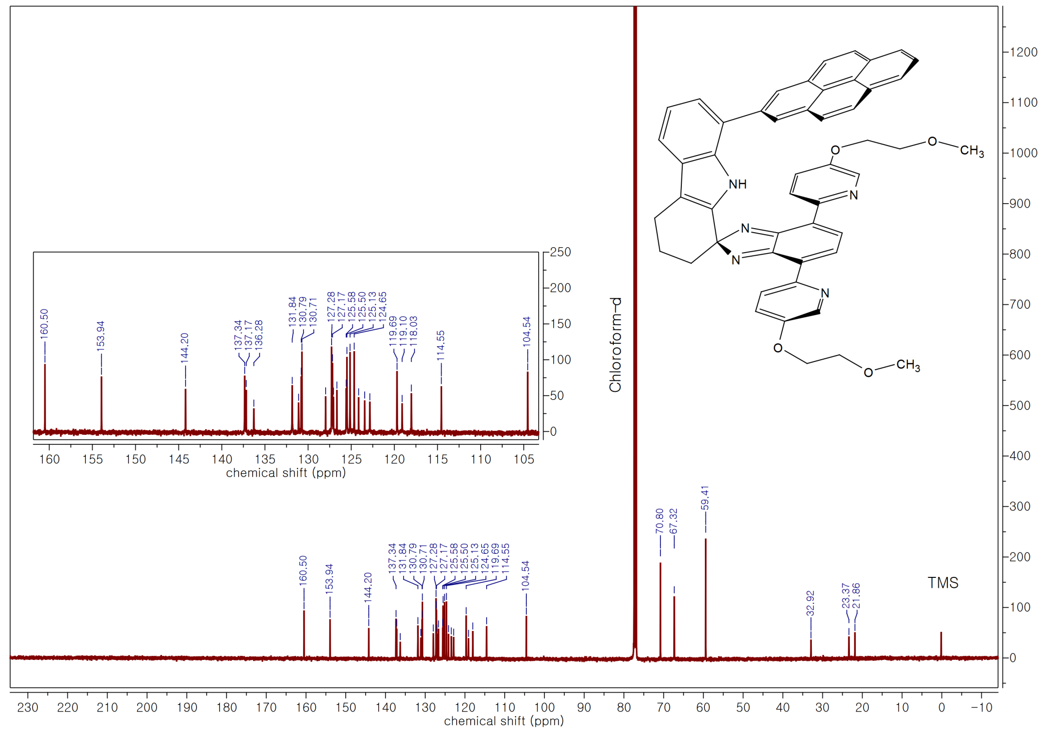

^{13}C NMR (125 MHz) spectrum of **C-P2** in $CDCl_3$ (T = 298 K).

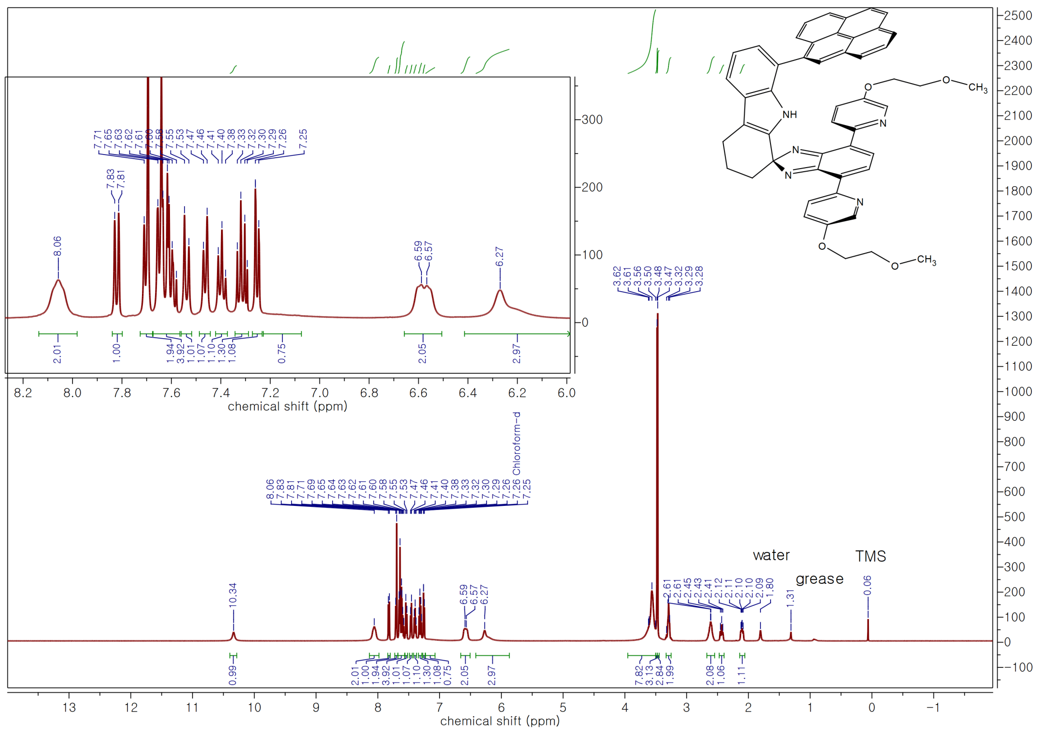

^{1}H NMR (500 MHz) spectrum of **C-P4** in $CDCl_3$ (T = 298 K).

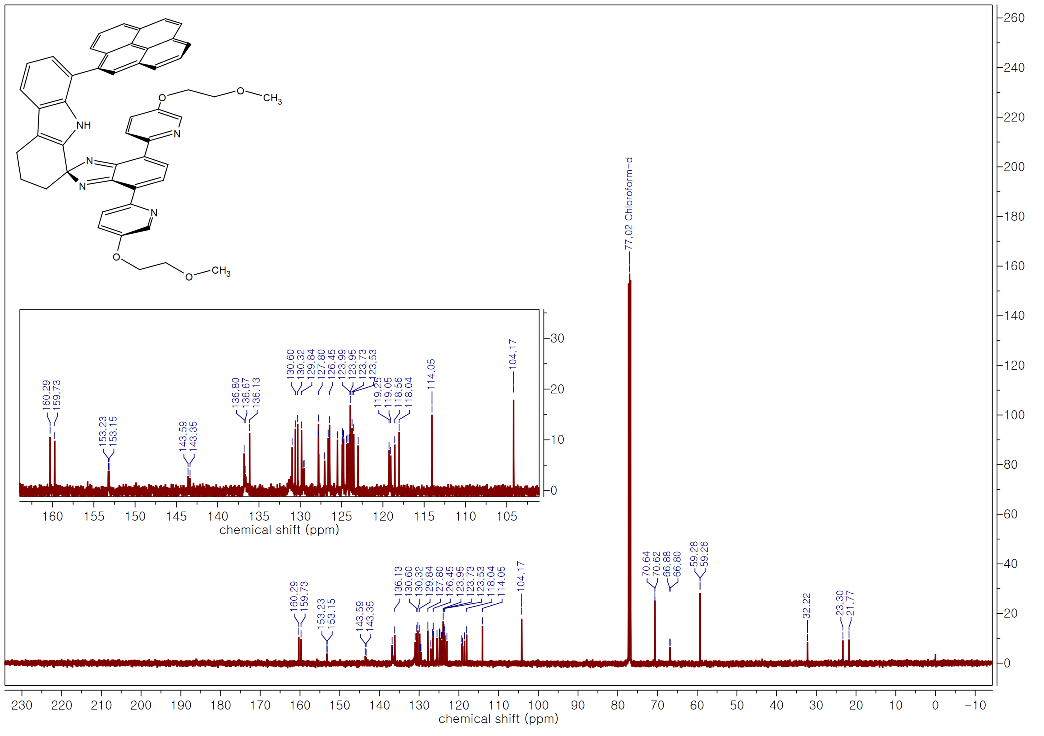

^{13}C NMR (125 MHz) spectrum of **C-P4** in $CDCl_3$ (T = 298 K).

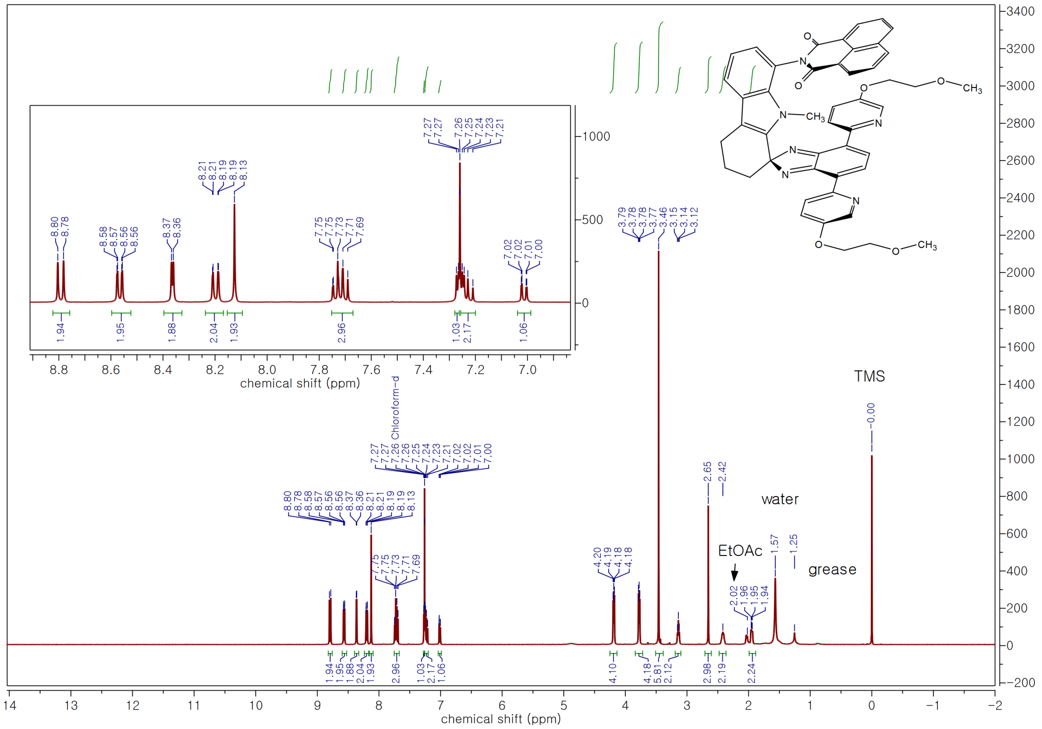

^{1}H NMR (400 MHz) spectrum of **C-NIMe** in $CDCl_3$ (T = 298 K).

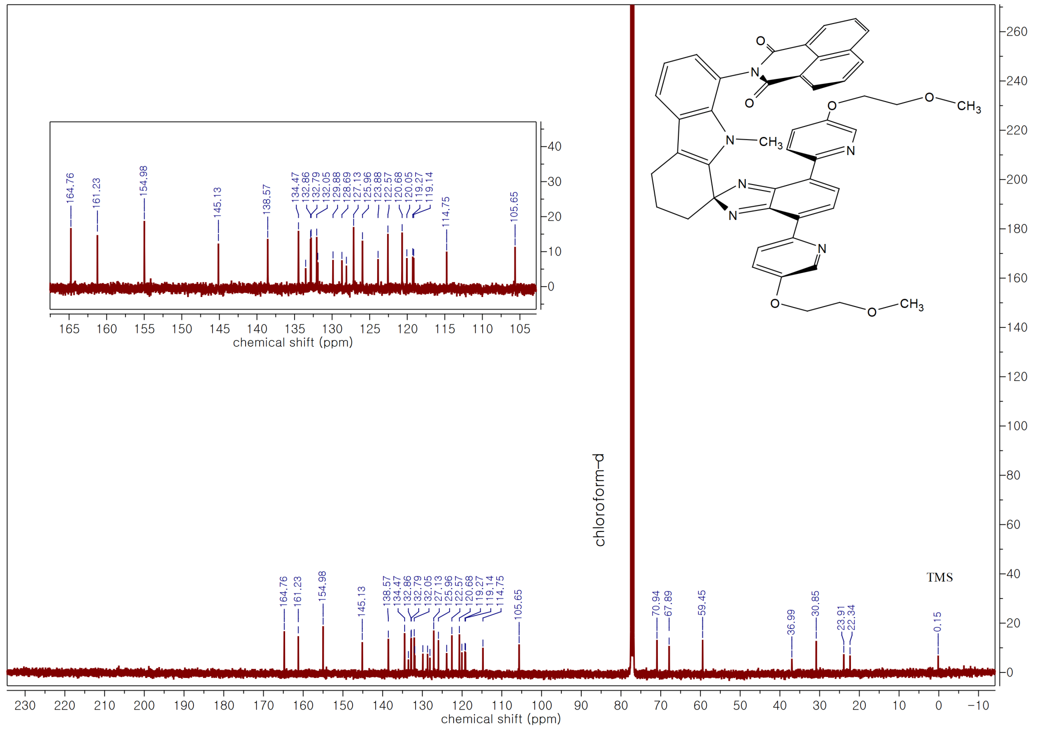

^{13}C NMR (100 MHz) spectrum of **C-NIMe** in $CDCl_3$ (T = 298 K).

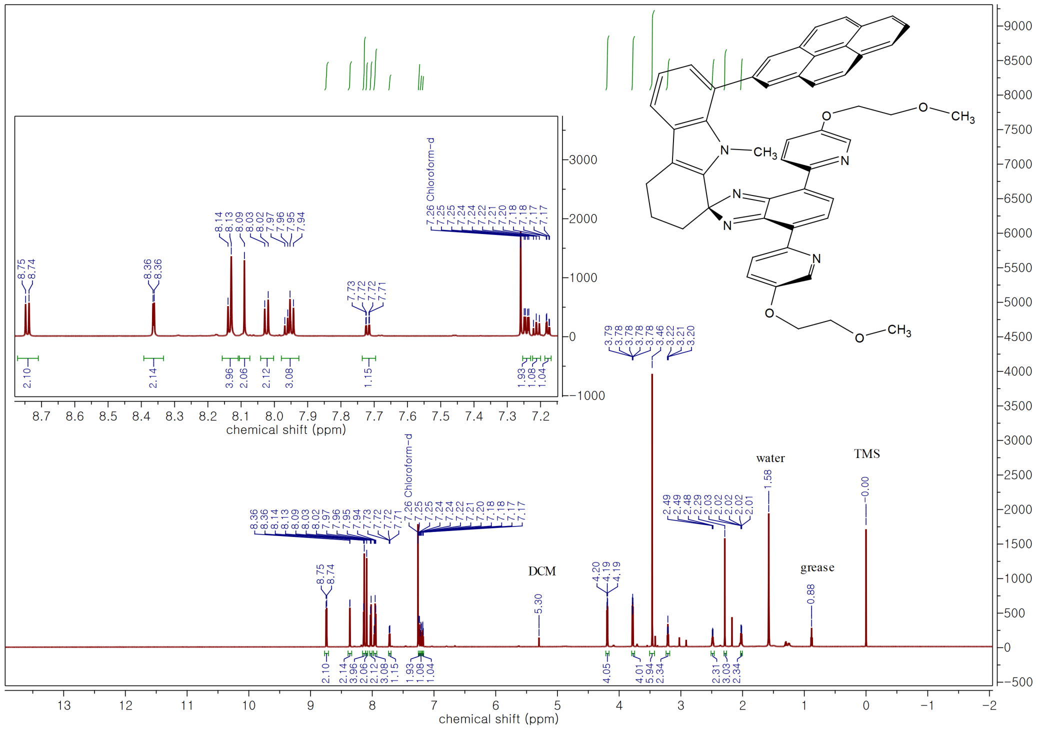

^{1}H NMR (850 MHz) spectrum of **C-P2Me** in $CDCl_3$ (T = 298 K).

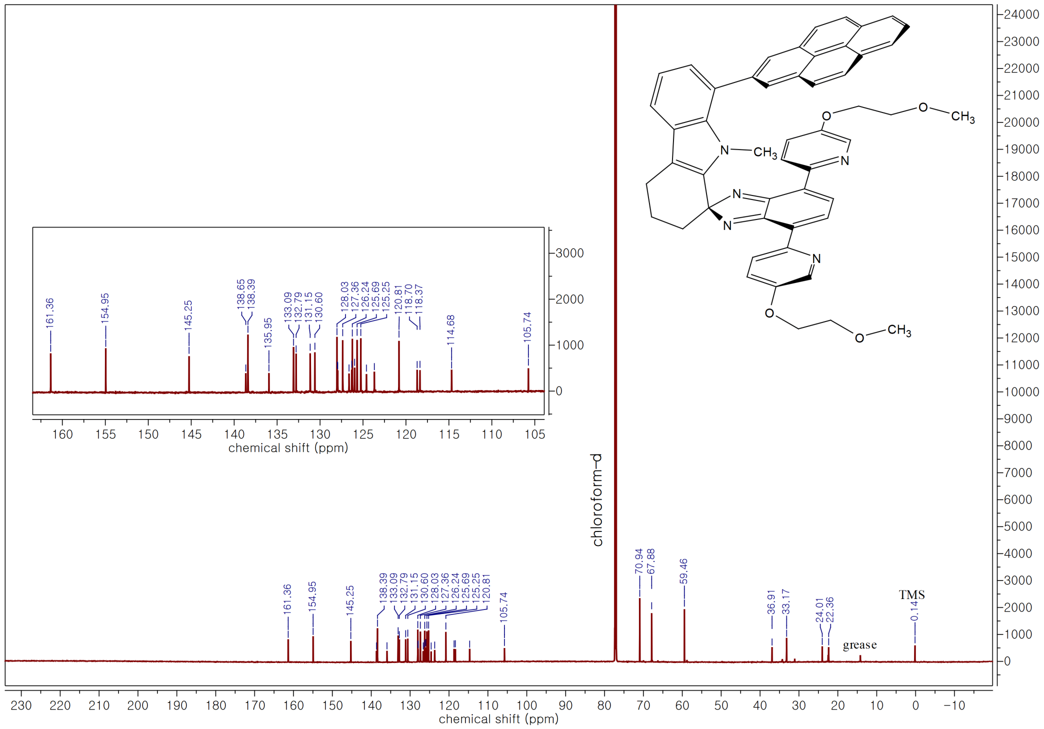

^{13}C NMR (213 MHz) spectrum of **C-P2Me** in $CDCl_3$ (T = 298 K).

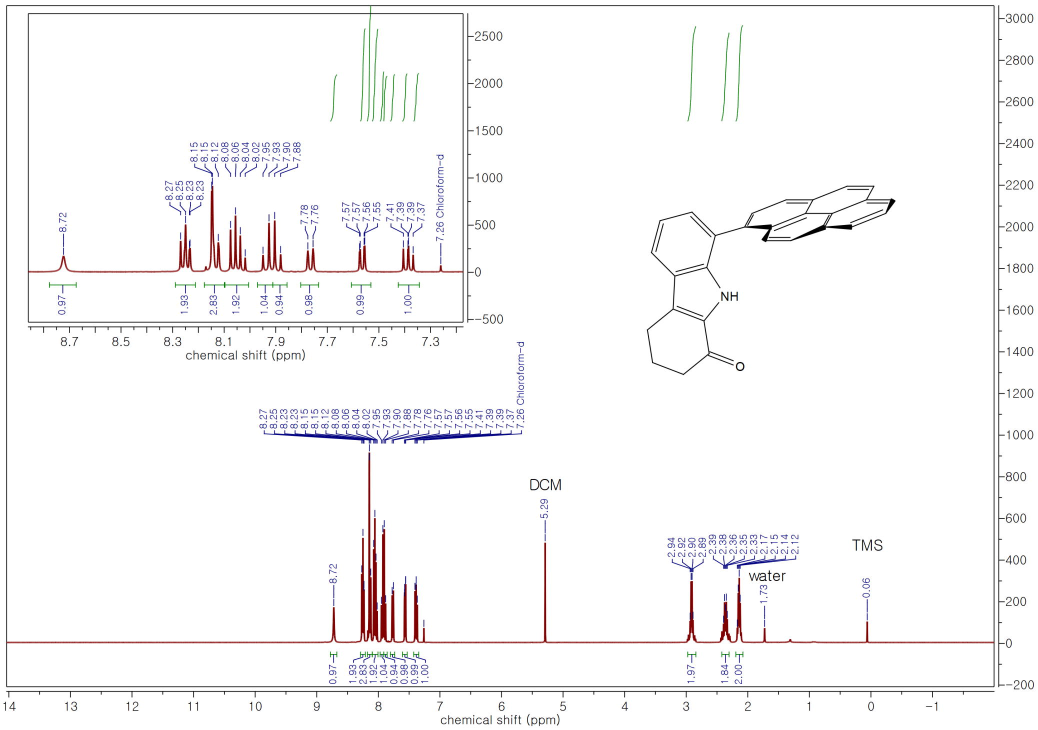

^{1}H NMR (400 MHz) spectrum of **2-P1** in $CDCl_3$ (T = 298 K).

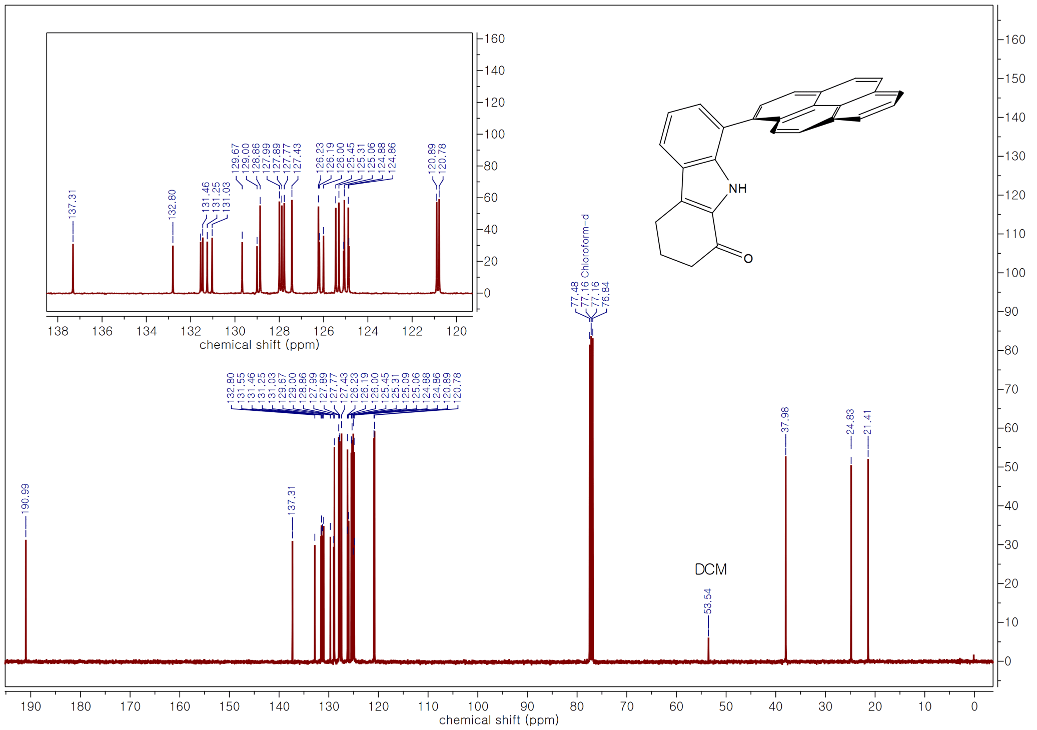

^{13}C NMR (100 MHz) spectrum of **2-P1** in $CDCl_3$ (T = 298 K).

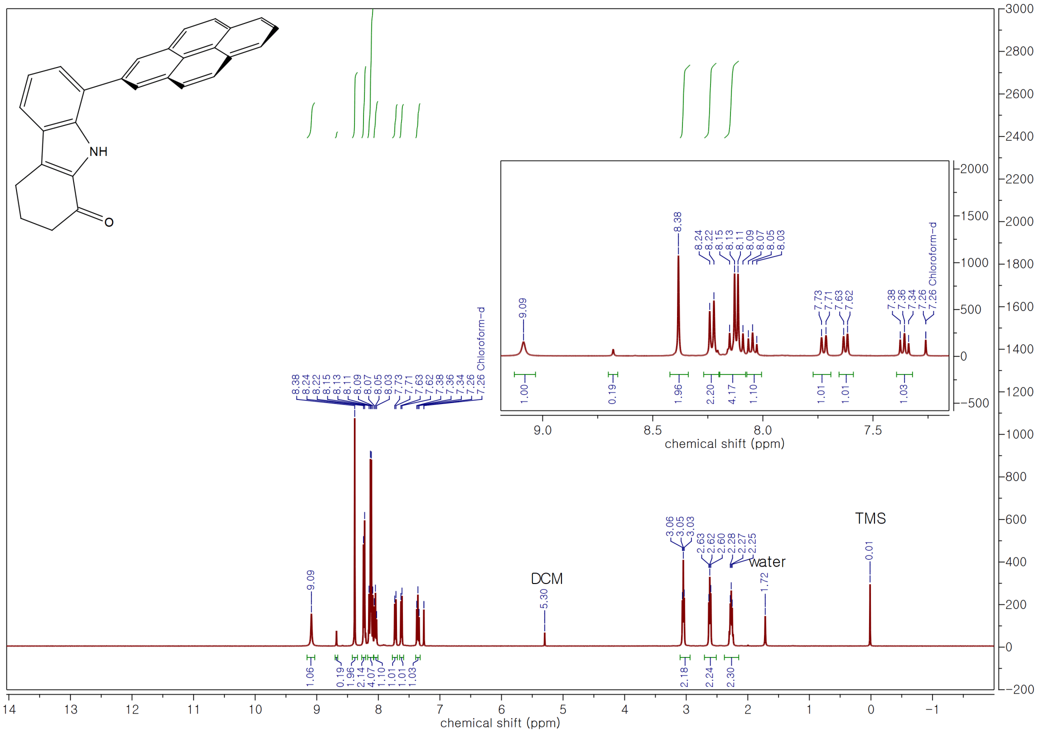

^{1}H NMR (400 MHz) spectrum of **2-P2** in $CDCl_3$ (T = 298 K).

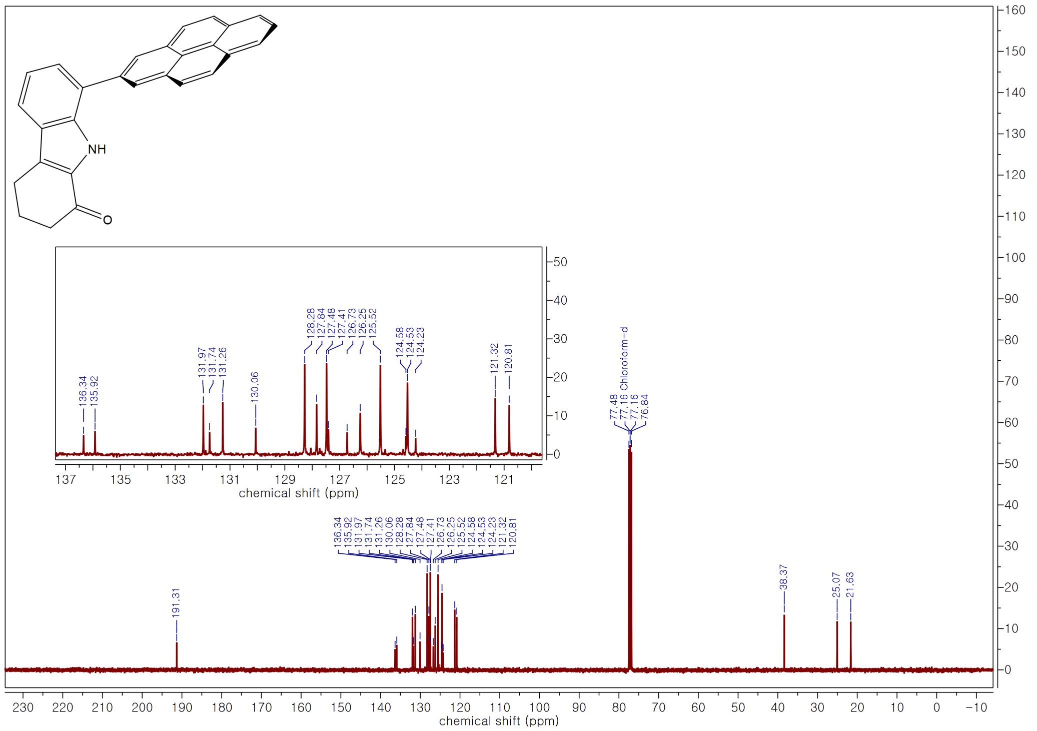

^{13}C NMR (100 MHz) spectrum of **2-P2** in $CDCl_3$ (T = 298 K).

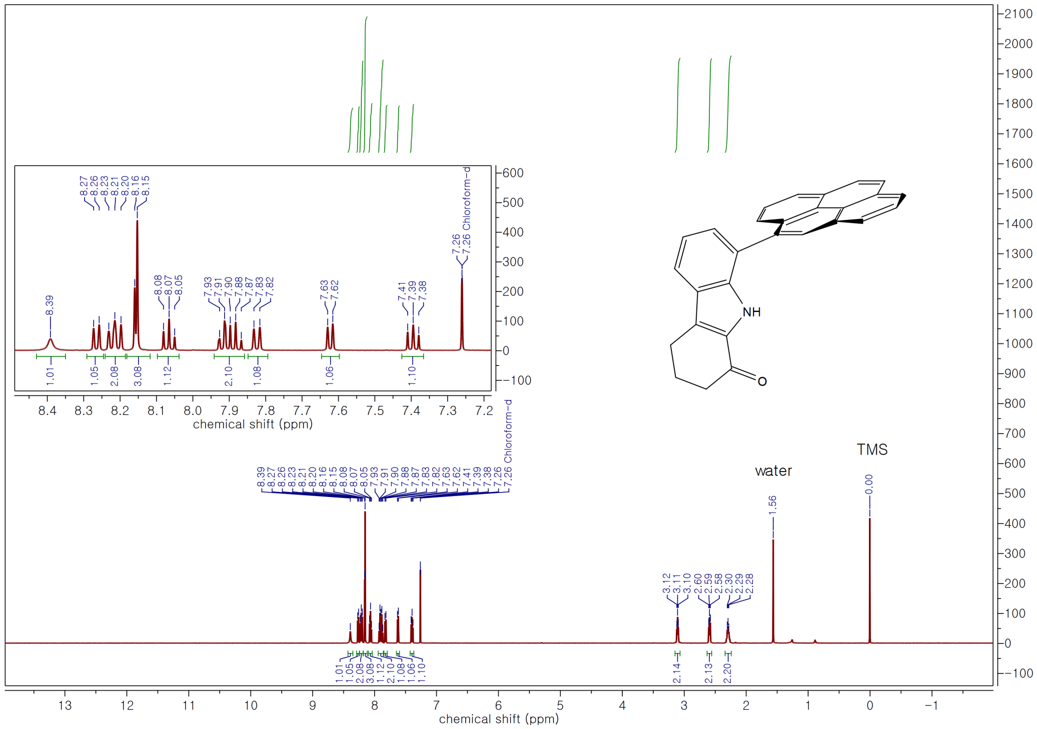

^{1}H NMR (500 MHz) spectrum of **2-P4** in $CDCl_3$ (T = 298 K).

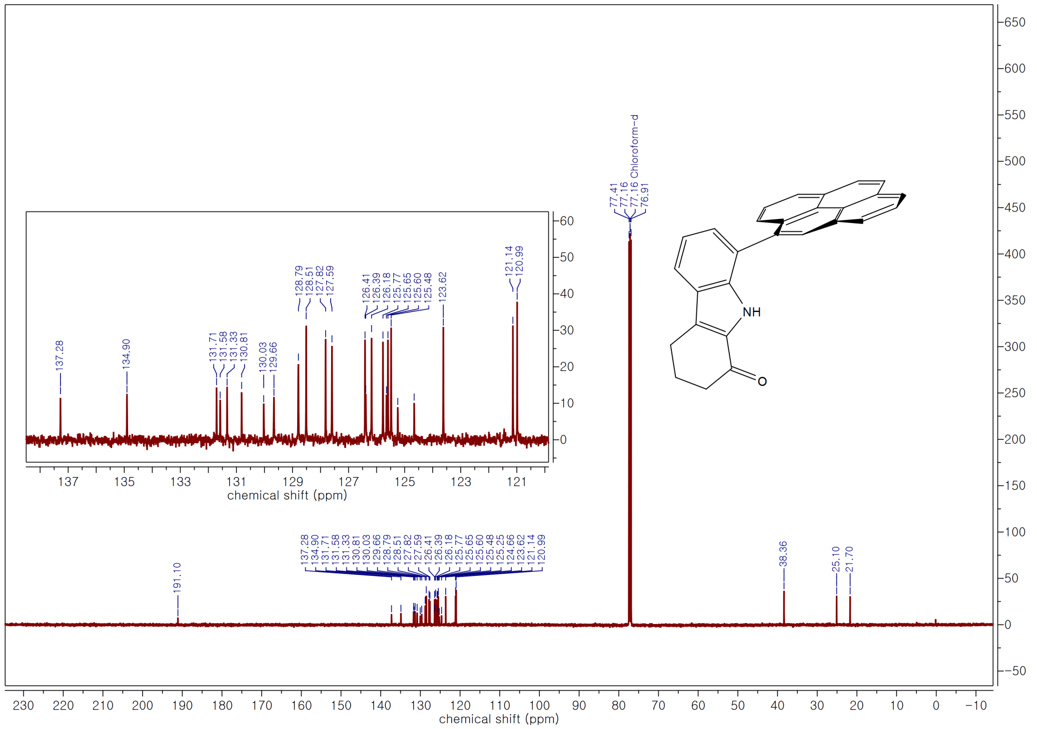

^{13}C NMR (125 MHz) spectrum of **2-P4** in $CDCl_3$ (T = 298 K).

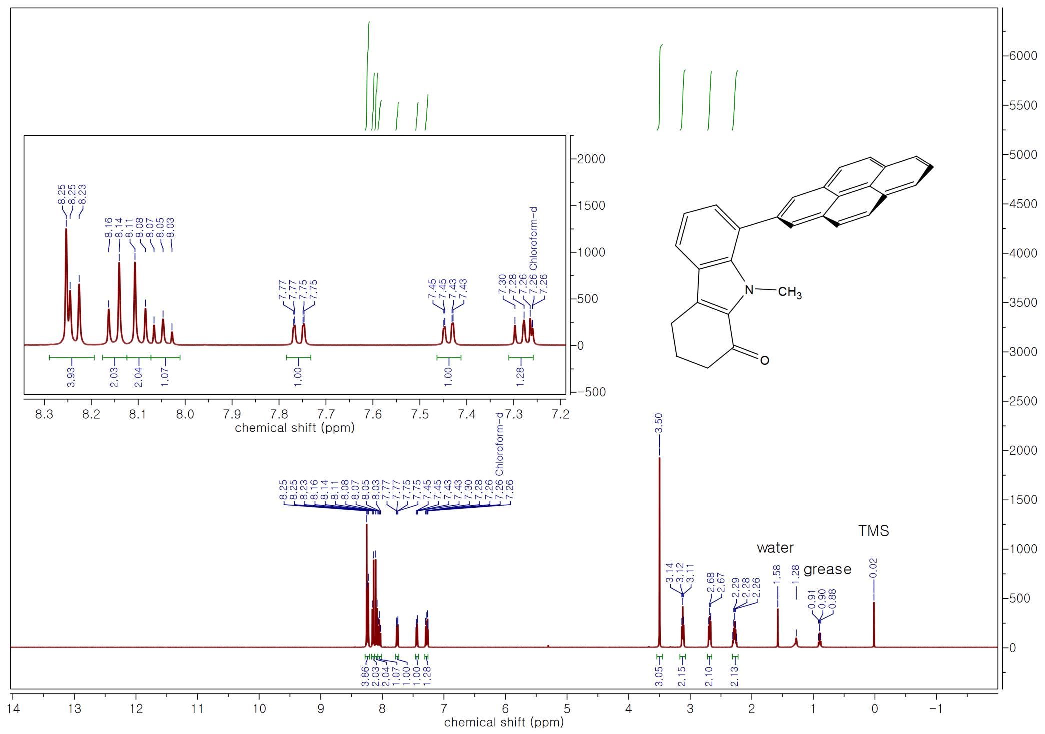

^{1}H NMR (500 MHz) spectrum of **2-P2Me** in $CDCl_3$ (T = 298 K).

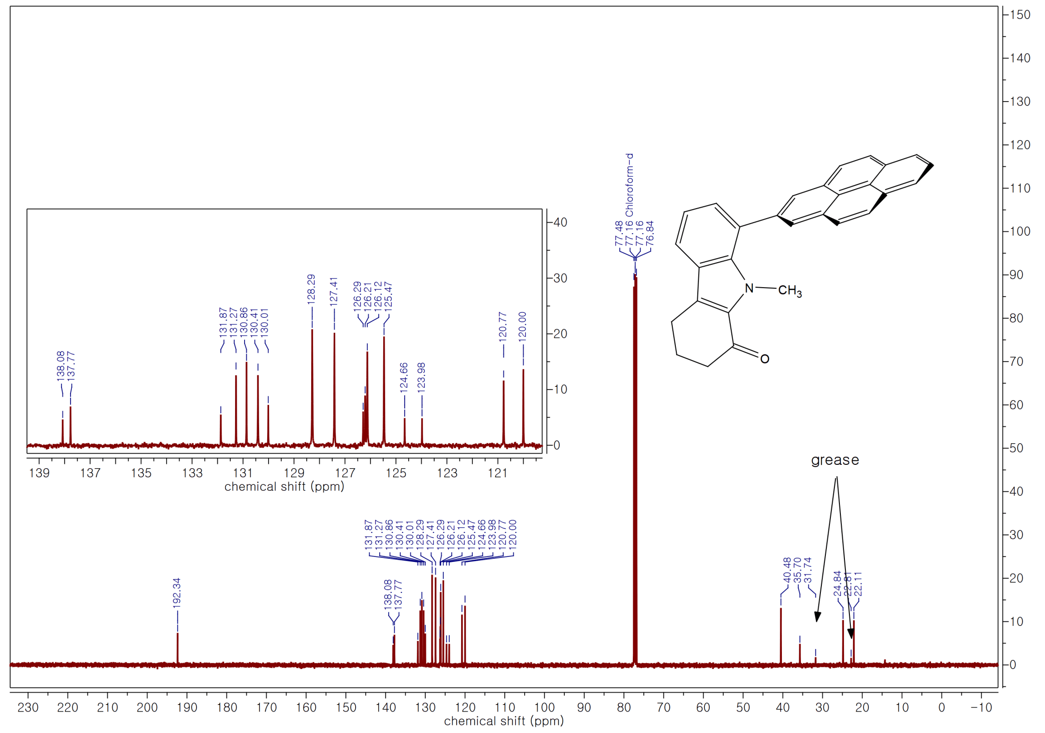

^{13}C NMR (125 MHz) spectrum of **2-P2Me** in $CDCl_3$ (T = 298 K).

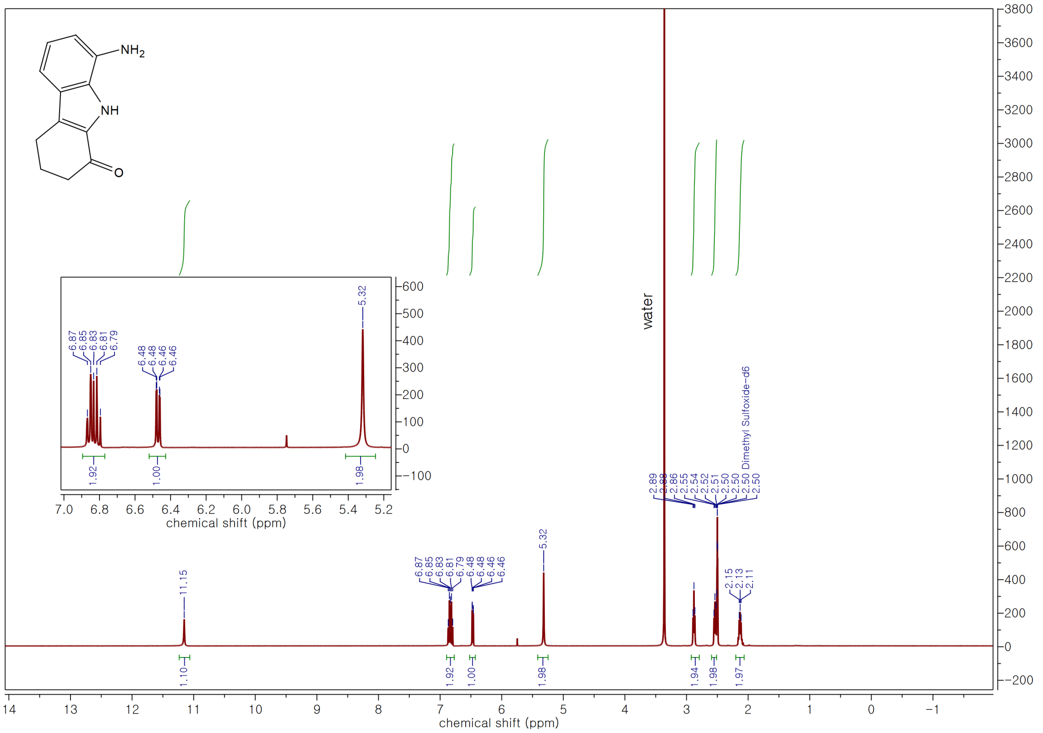

^{1}H NMR (400 MHz) spectrum of **3** in DMSO-d_6 (T = 298 K).

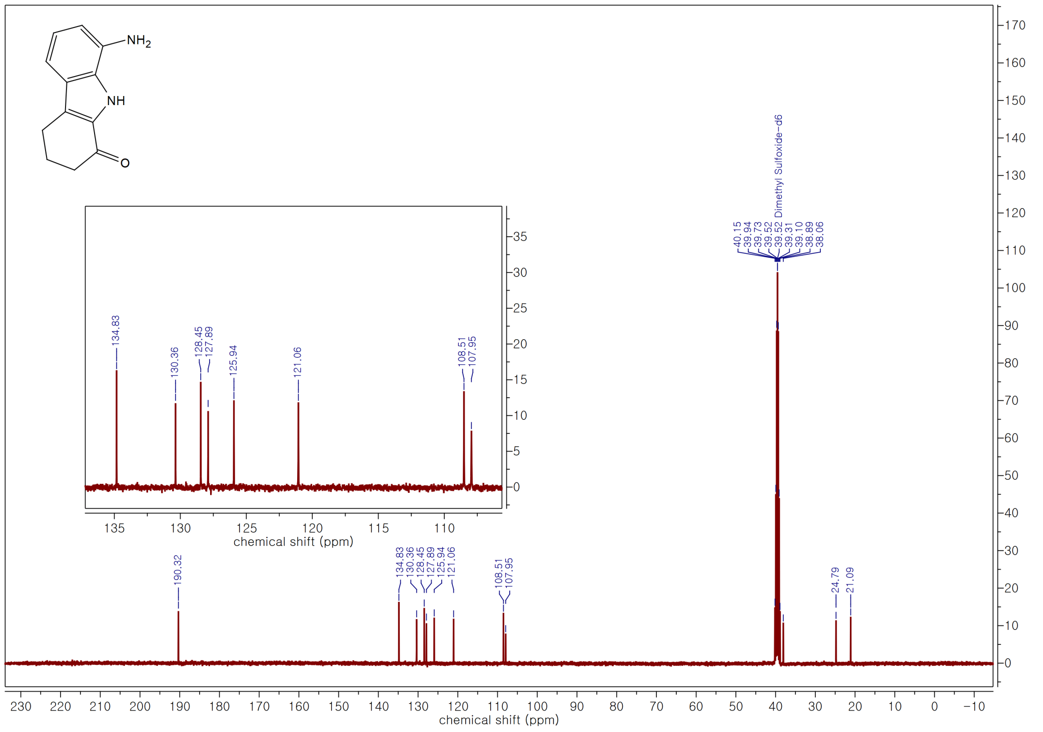

^{13}C NMR (100 MHz) spectrum of **3** in DMSO-d_6 (T = 298 K).

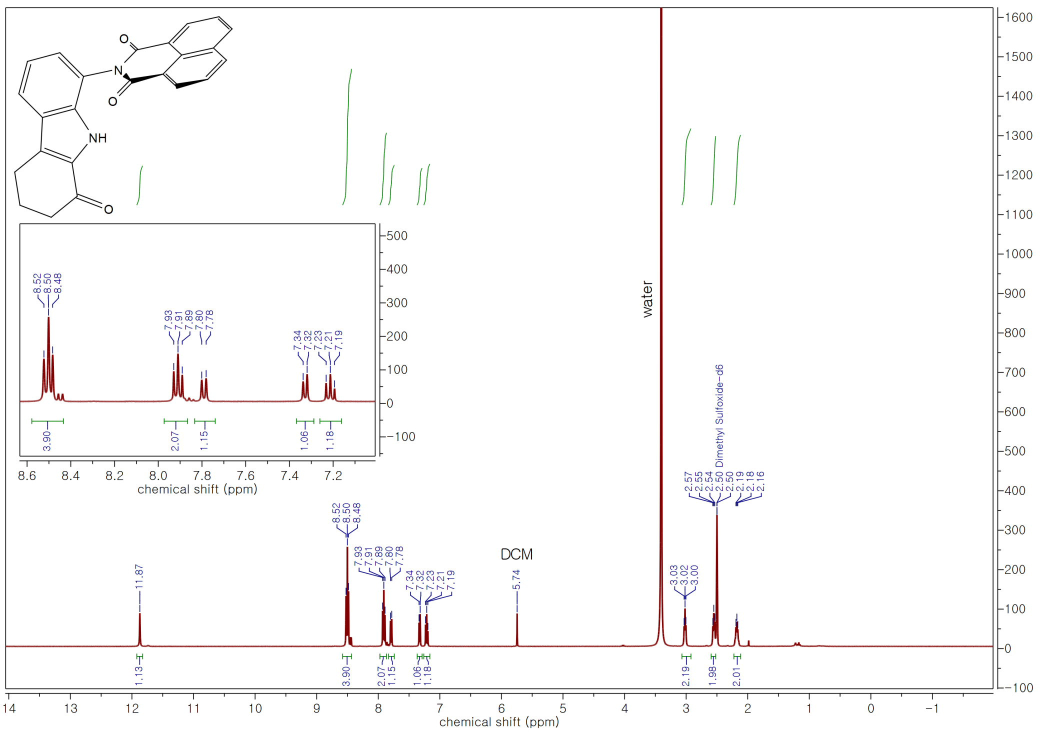

^{1}H NMR (400 MHz) spectrum of **4** in DMSO-d_6 (T = 298 K).

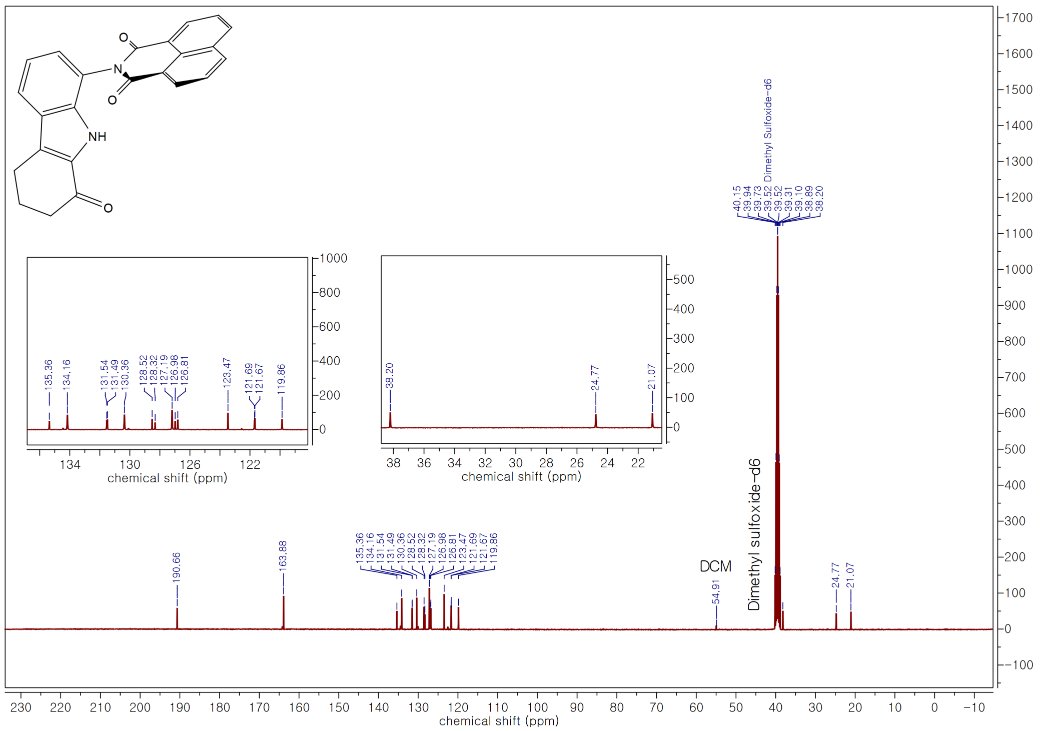

^{13}C NMR (100 MHz) spectrum of **4** in DMSO-d_6 (T = 298 K).

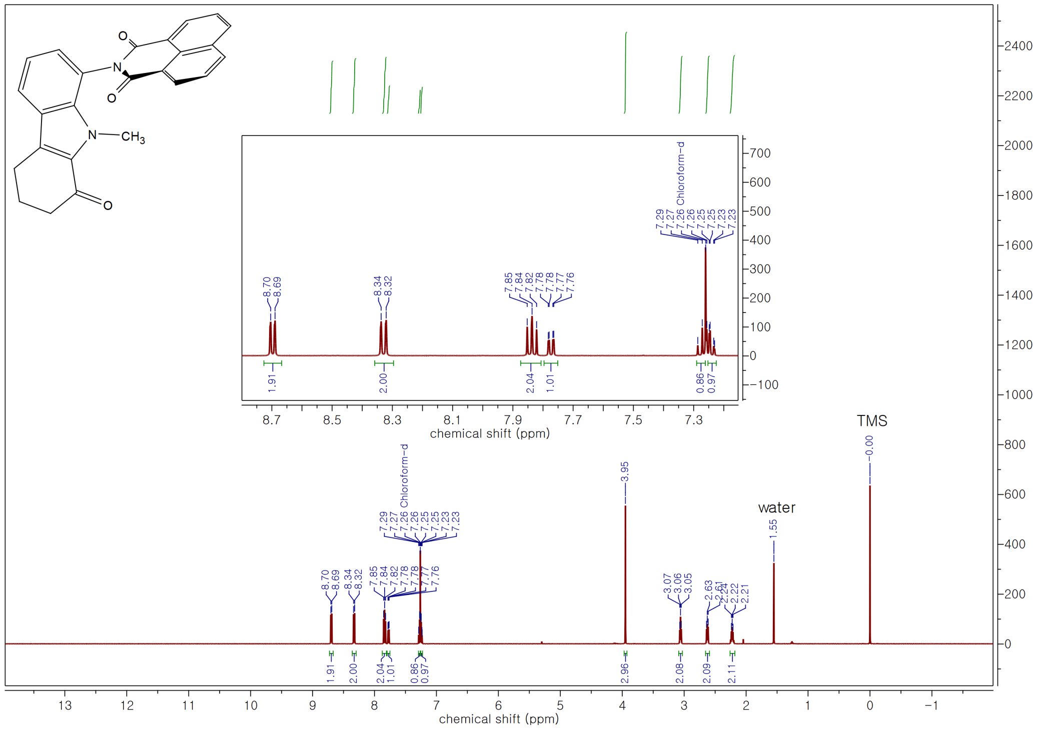

^{1}H NMR (500 MHz) spectrum of **4-Me** in $CDCl_3$ (T = 298 K).

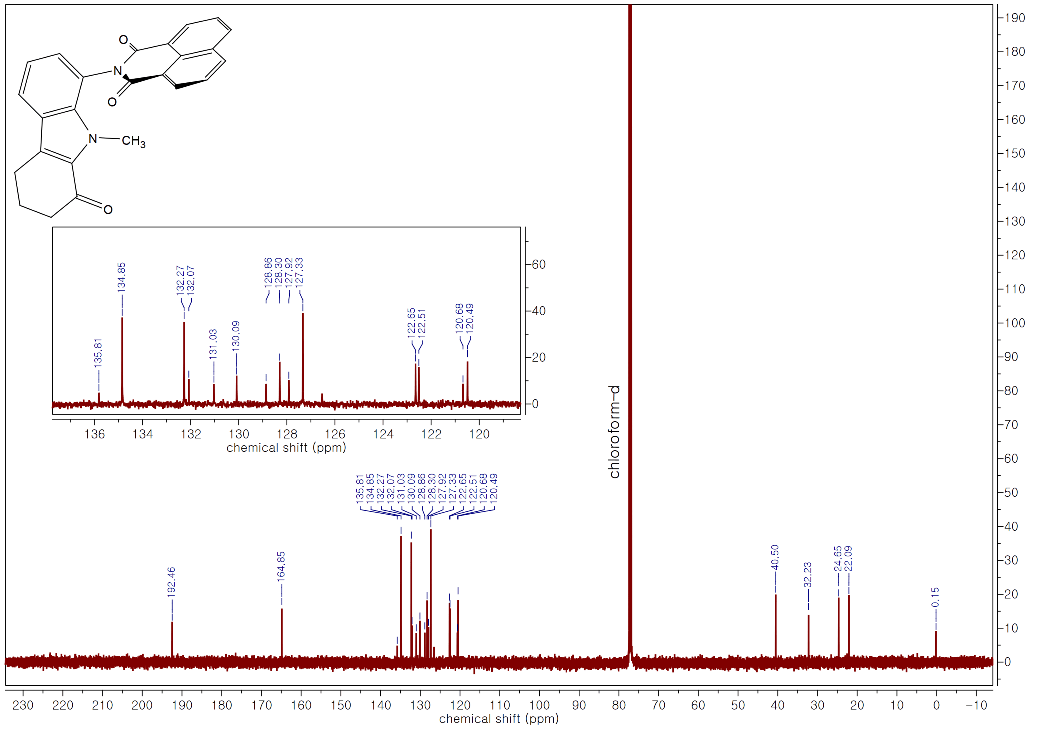

^{13}C NMR (125 MHz) spectrum of **4-Me** in $CDCl_3$ (T = 298 K).

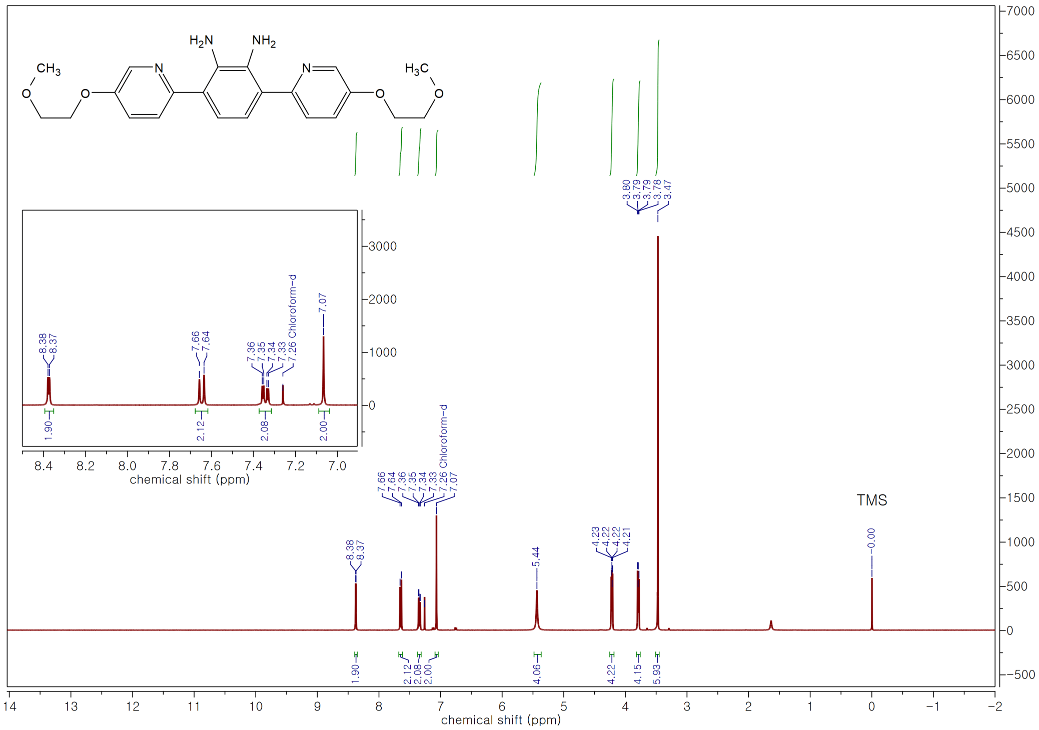

^{1}H NMR (400 MHz) spectrum of **5** in $CDCl_3$ (T = 298 K).

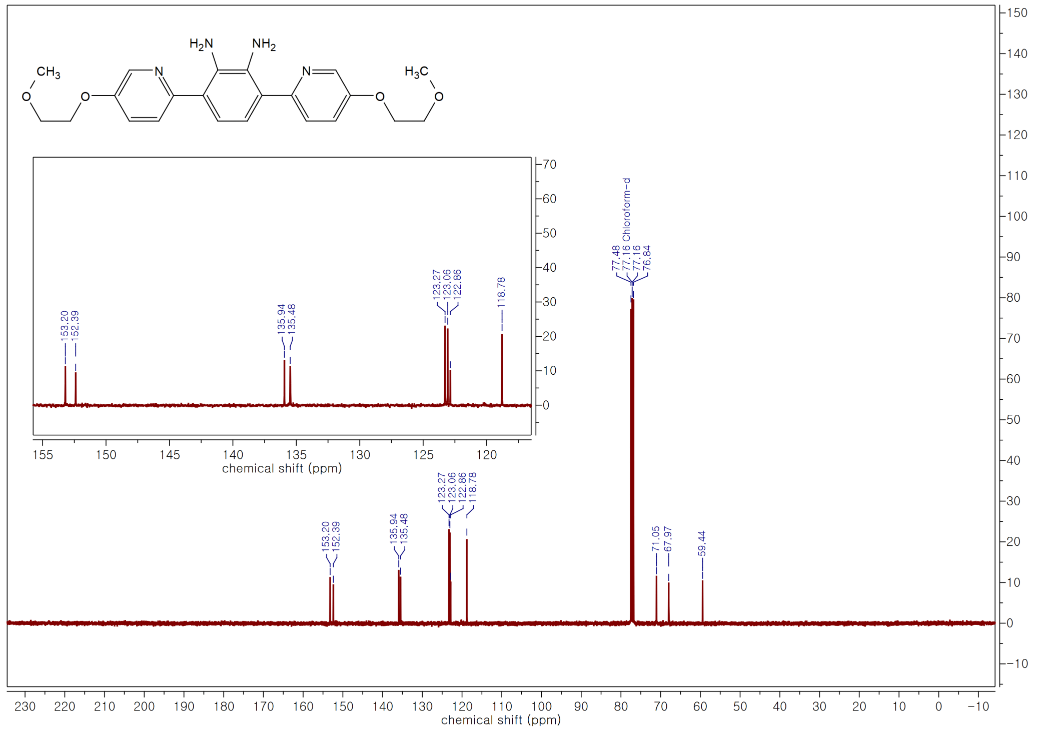

^{13}C NMR (100 MHz) spectrum of **5** in $CDCl_3$ (T = 298 K).

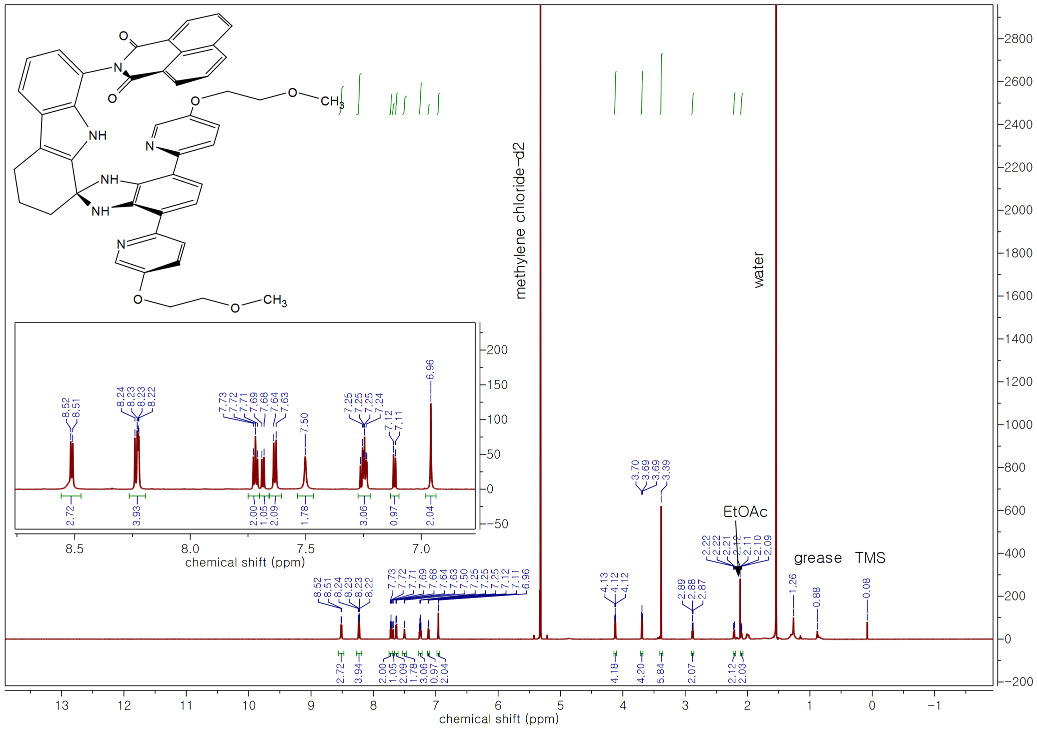

^{1}H NMR (850 MHz) spectrum of **6-NI** in CD_2Cl_2 (T = 298 K).

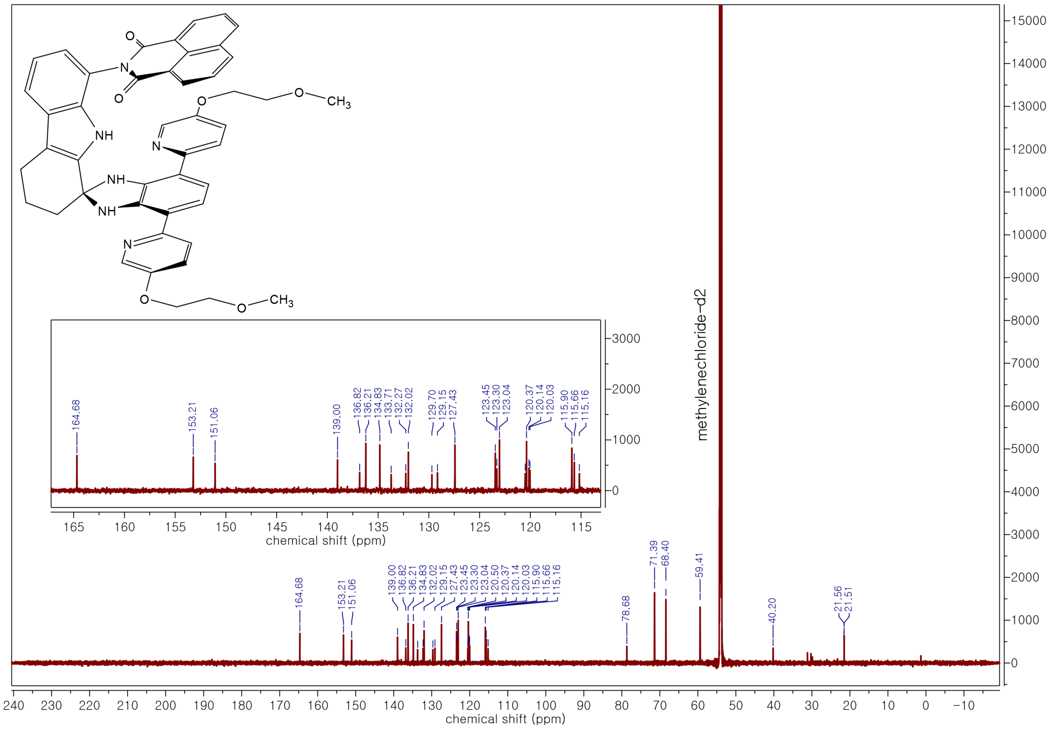

^{13}C NMR (213 MHz) spectrum of **6-NI** in CD_2Cl_2 (T = 298 K).

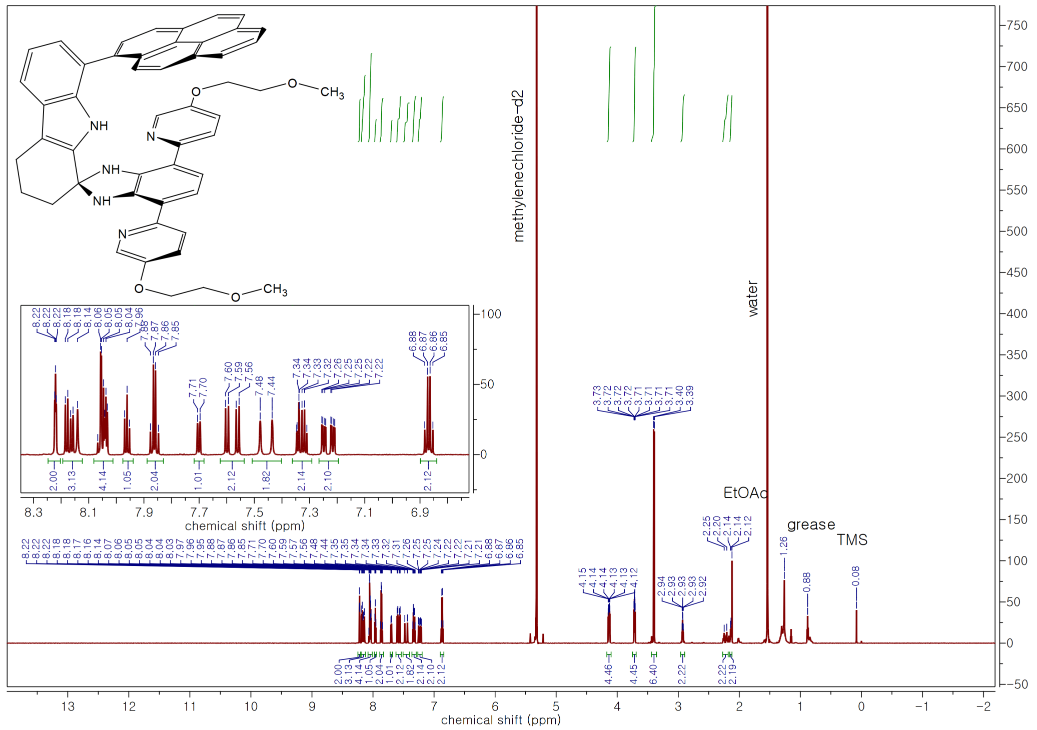

^{1}H NMR (850 MHz) spectrum of **6-P1** in CD_2Cl_2 (T = 298 K).

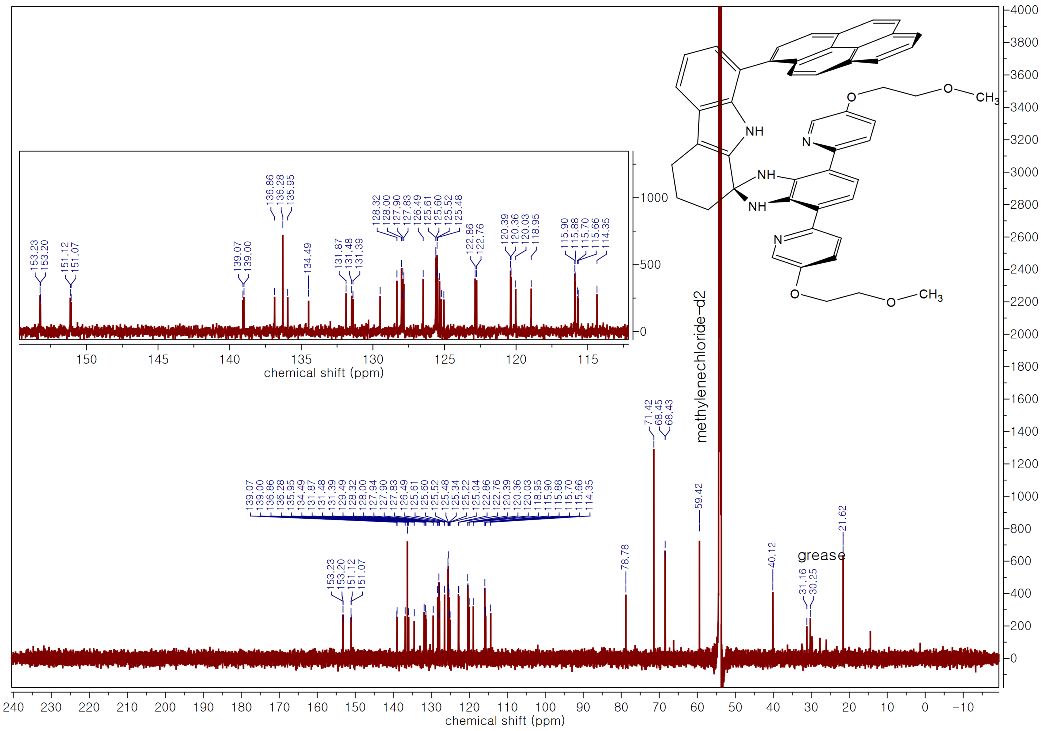

^{13}C NMR (213 MHz) spectrum of **6-P1** in CD_2Cl_2 (T = 298 K).

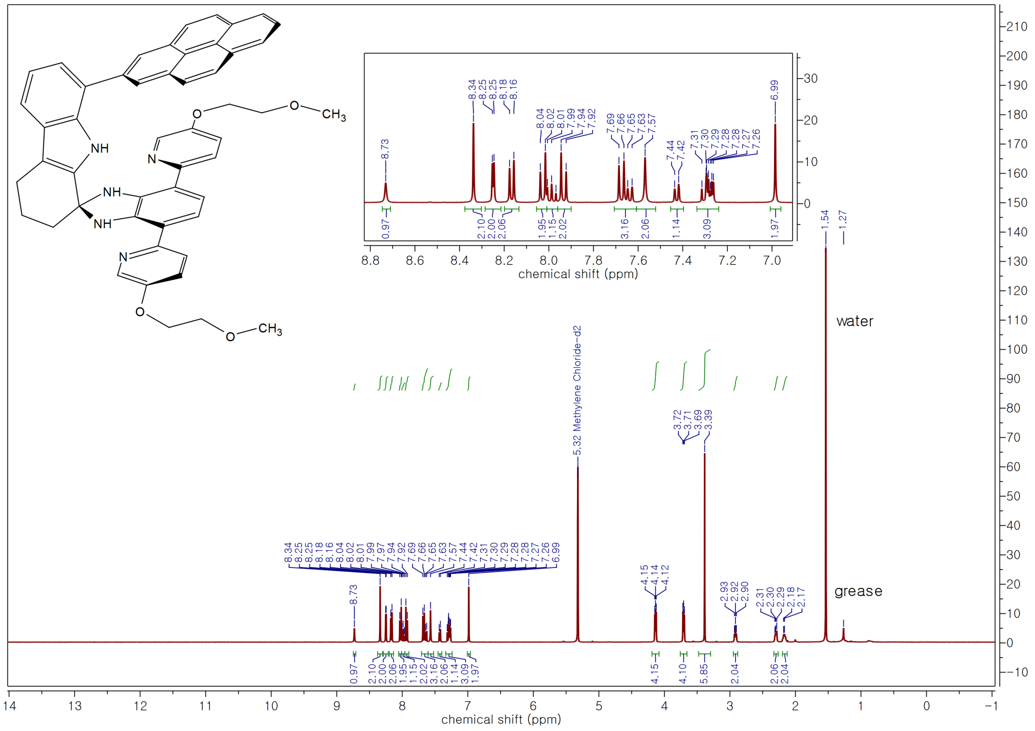

^{1}H NMR (400 MHz) spectrum of **6-P2** in CD_2Cl_2 (T = 298 K).

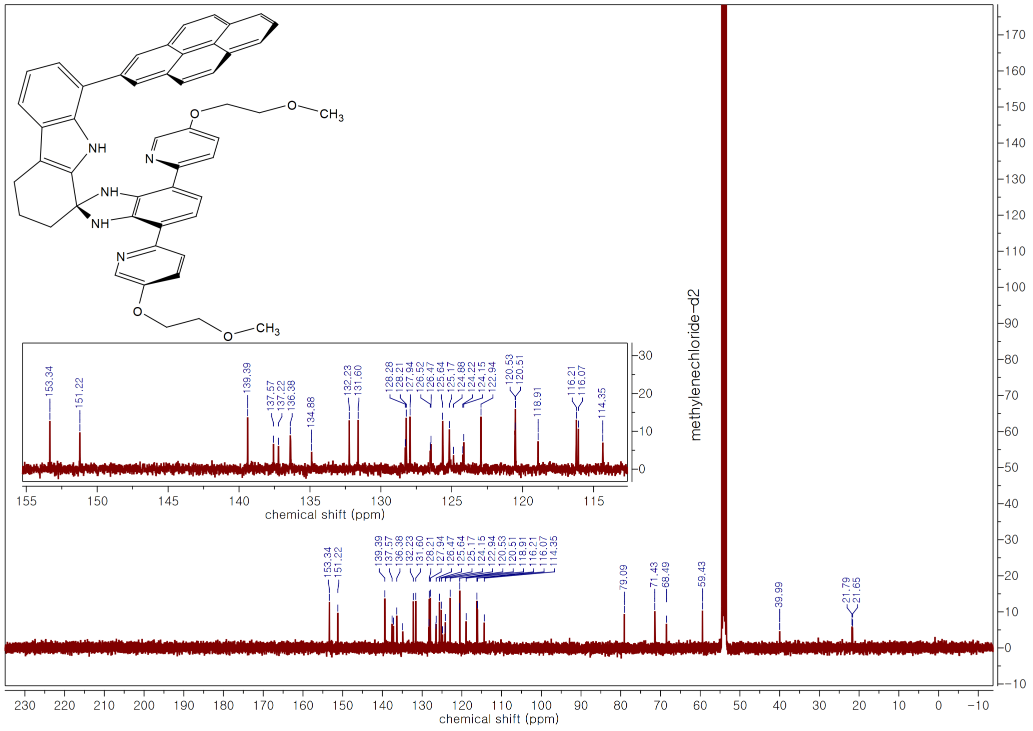

^{13}C NMR (100 MHz) spectrum of **6-P2** in CD_2Cl_2 (T = 298 K).

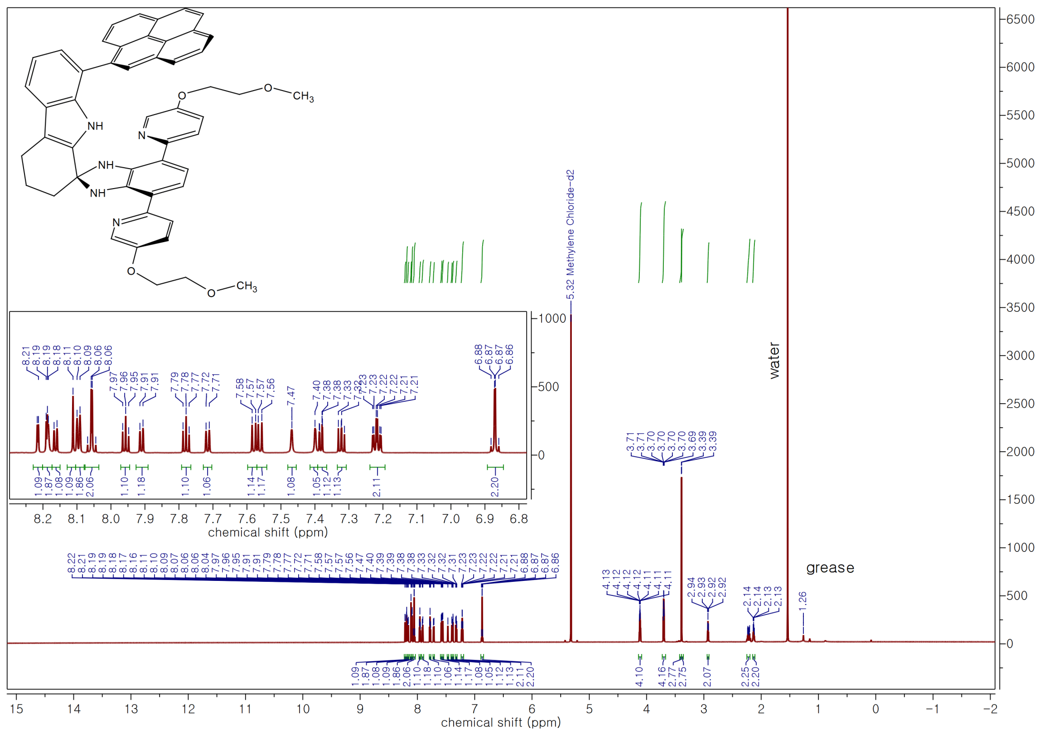

^1H NMR (850 MHz) spectrum of **6-P4** in CD_2Cl_2 (T = 298 K).

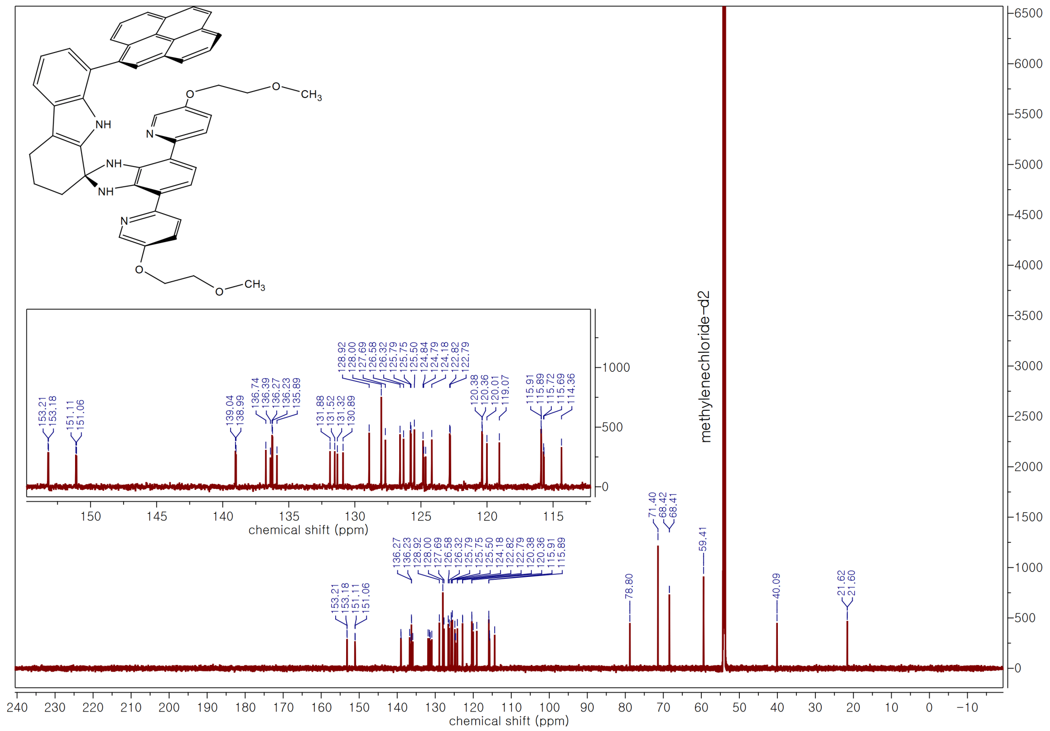

^{13}C NMR (213 MHz) spectrum of **6-P4** in CD_2Cl_2 (T = 298 K).

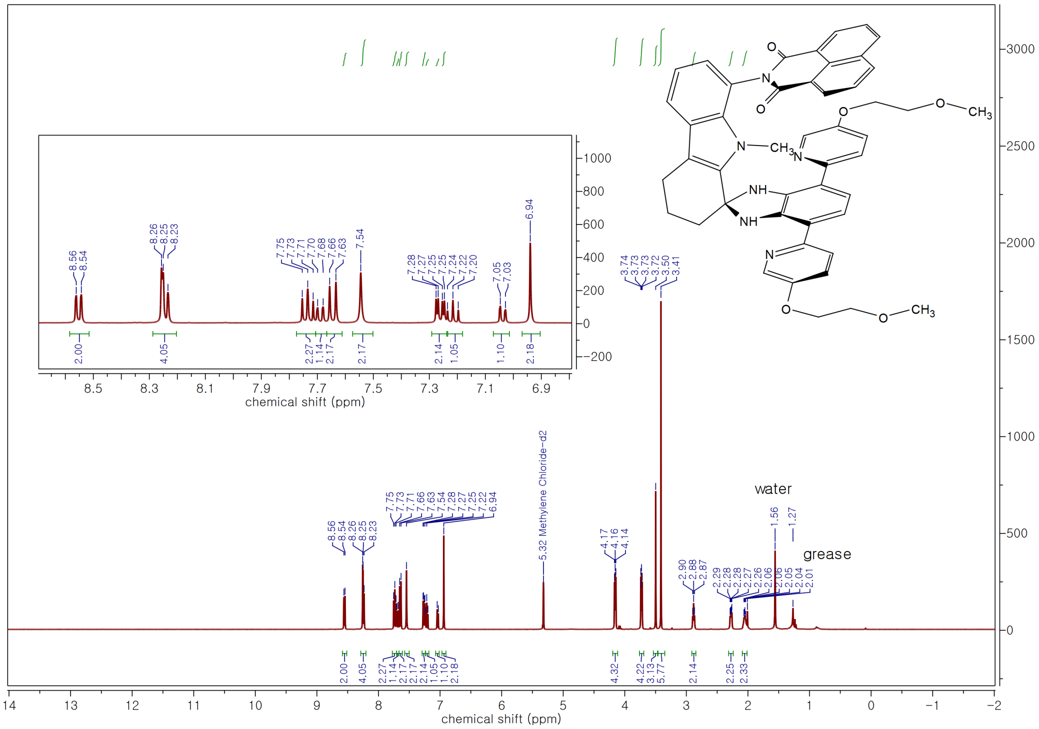

^{1}H NMR (400 MHz) spectrum of **6-NIMe** in CD_2Cl_2 (T = 298 K).

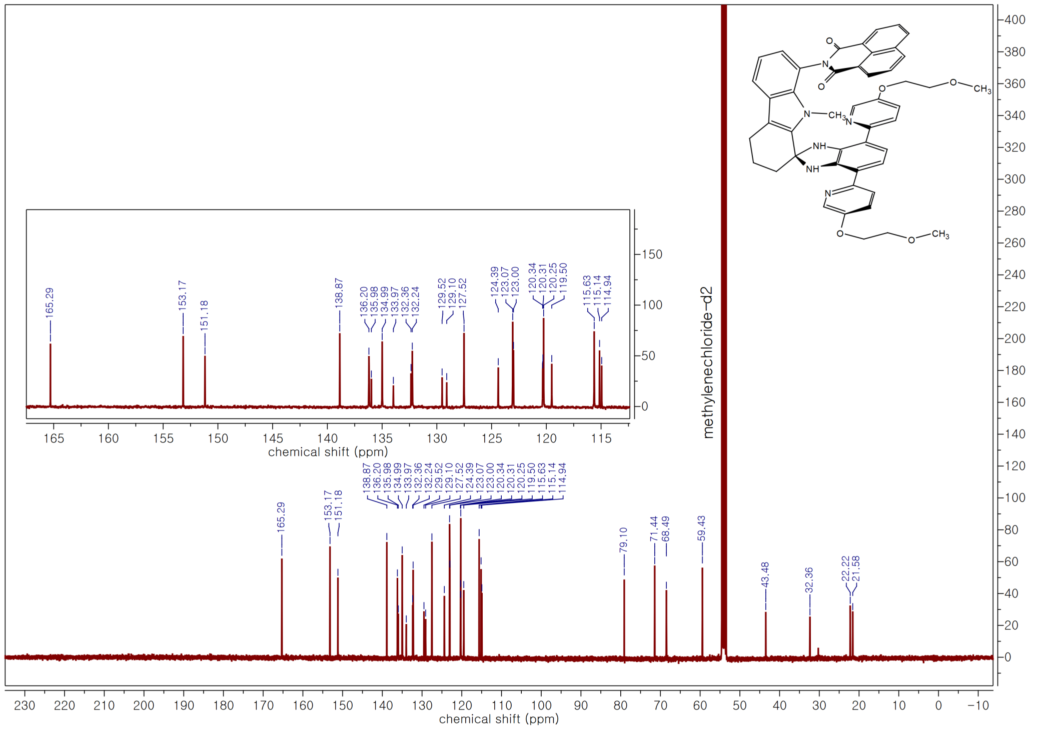

^{13}C NMR (100 MHz) spectrum of **6-NIMe** in CD_2Cl_2 (T = 298 K).

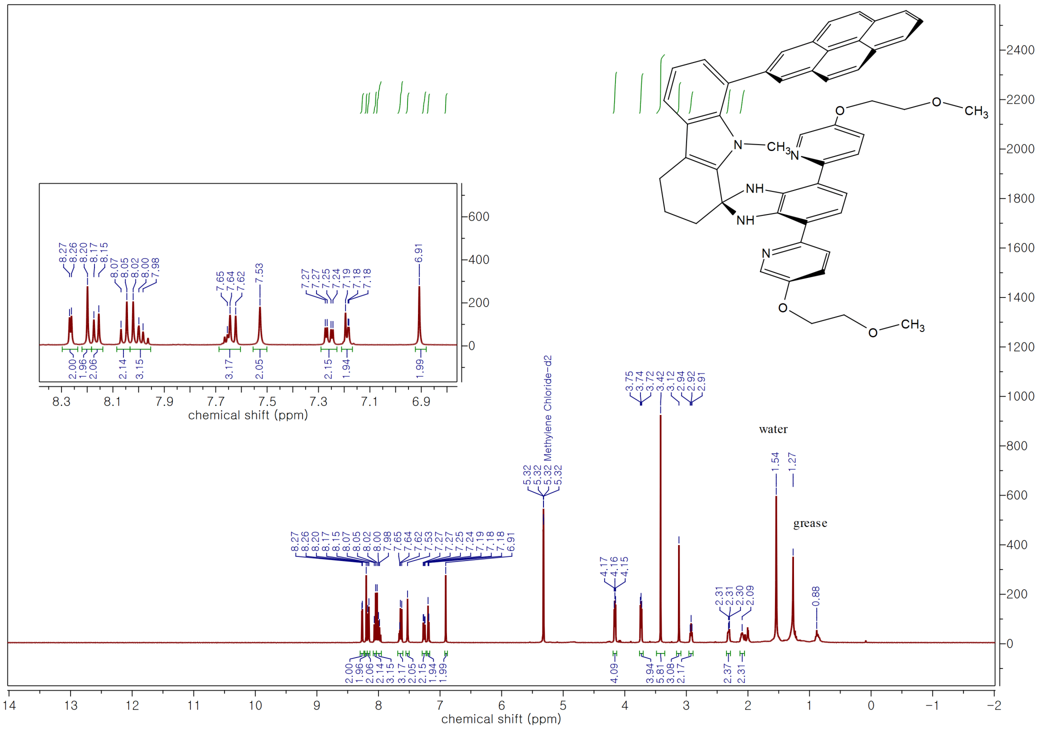

^{1}H NMR (400 MHz) spectrum of **6-P2Me** in CD_2Cl_2 (T = 298 K).

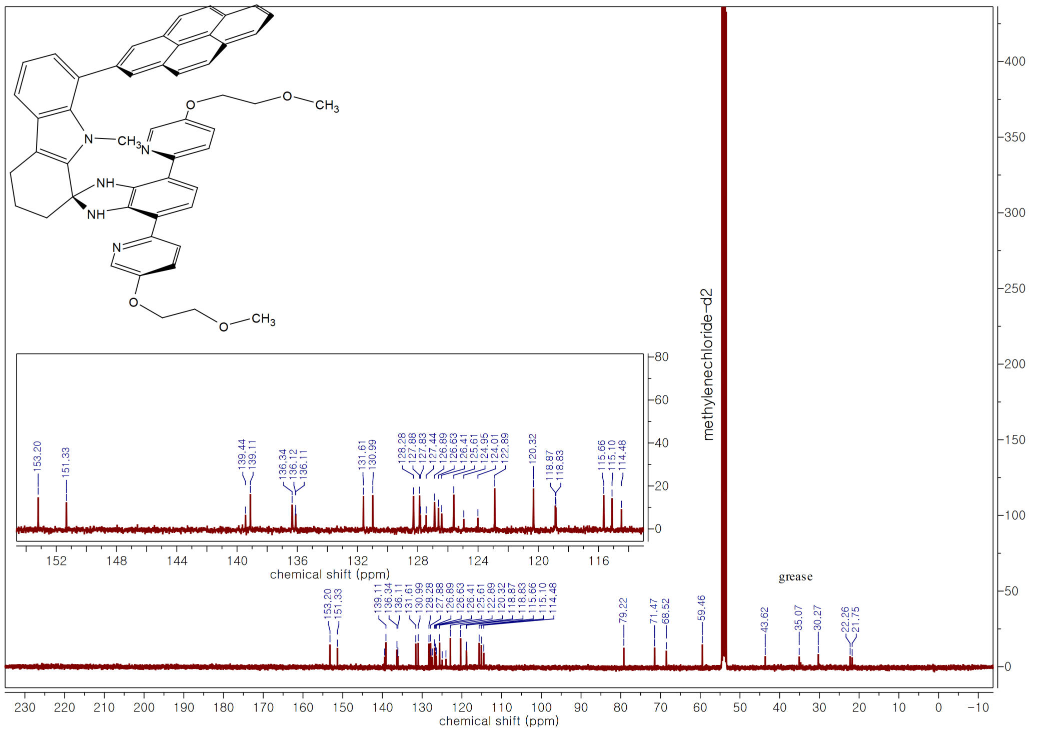

^{13}C NMR (100 MHz) spectrum of **6-P2Me** in CD_2Cl_2 (T = 298 K).

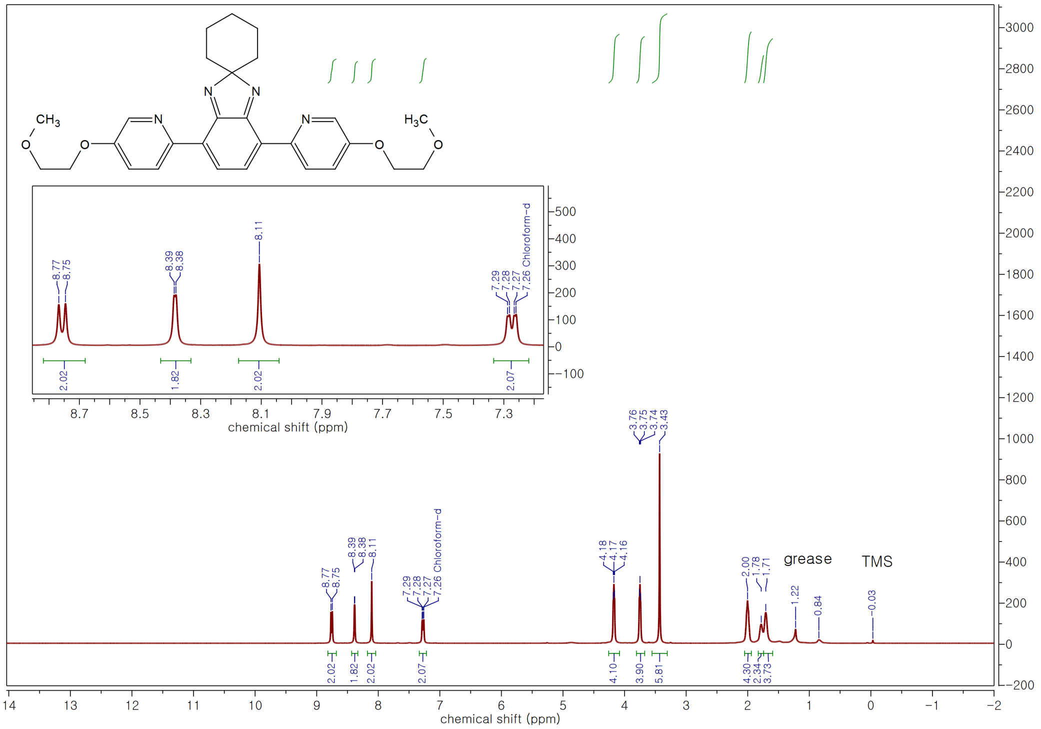

^{1}H NMR (400 MHz) spectrum of **7** in $CDCl_3$ (T = 298 K).

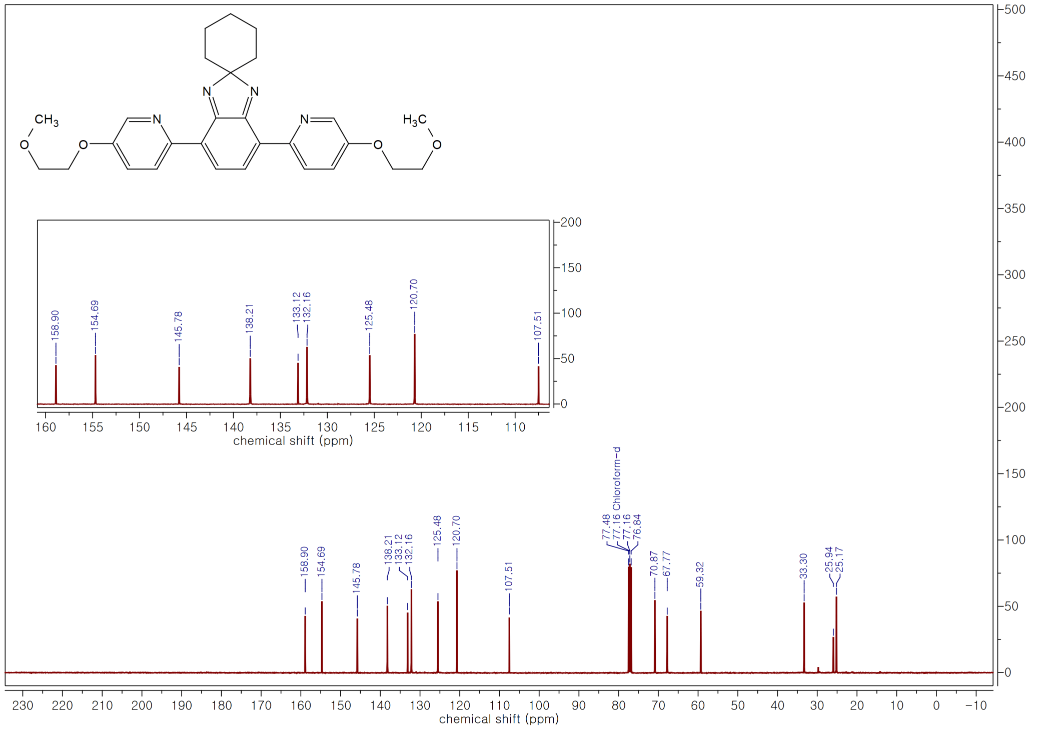

^{13}C NMR (100 MHz) spectrum of **7** in $CDCl_3$ (T = 298 K).

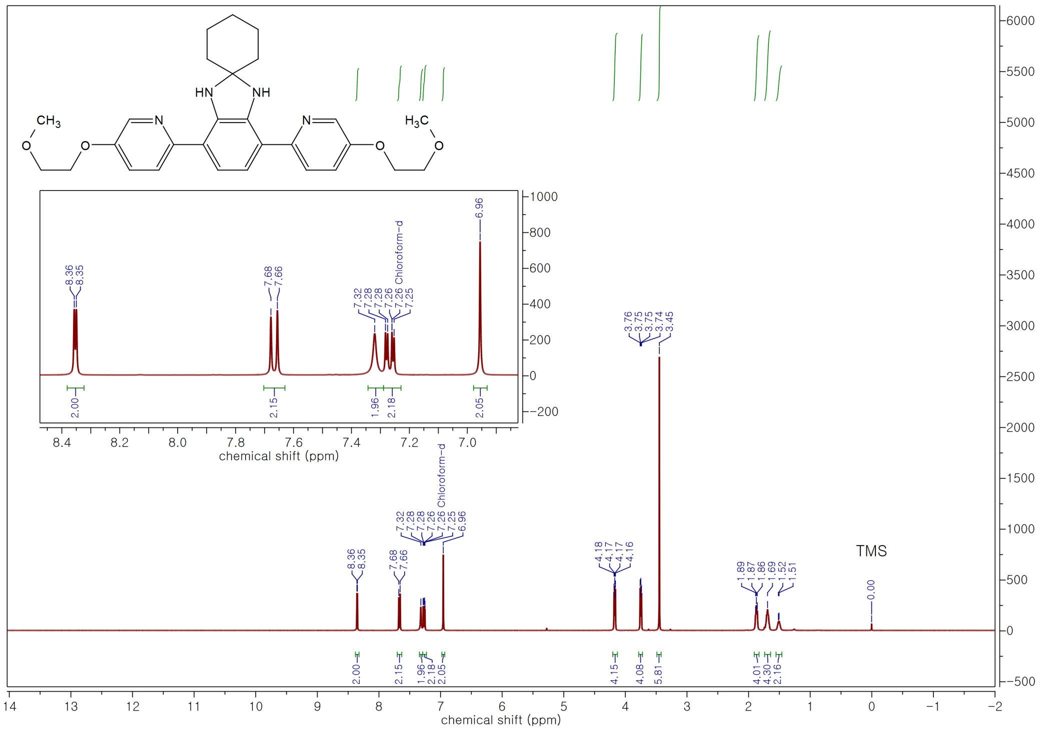

^{1}H NMR (400 MHz) spectrum of **8** in $CDCl_3$ (T = 298 K).

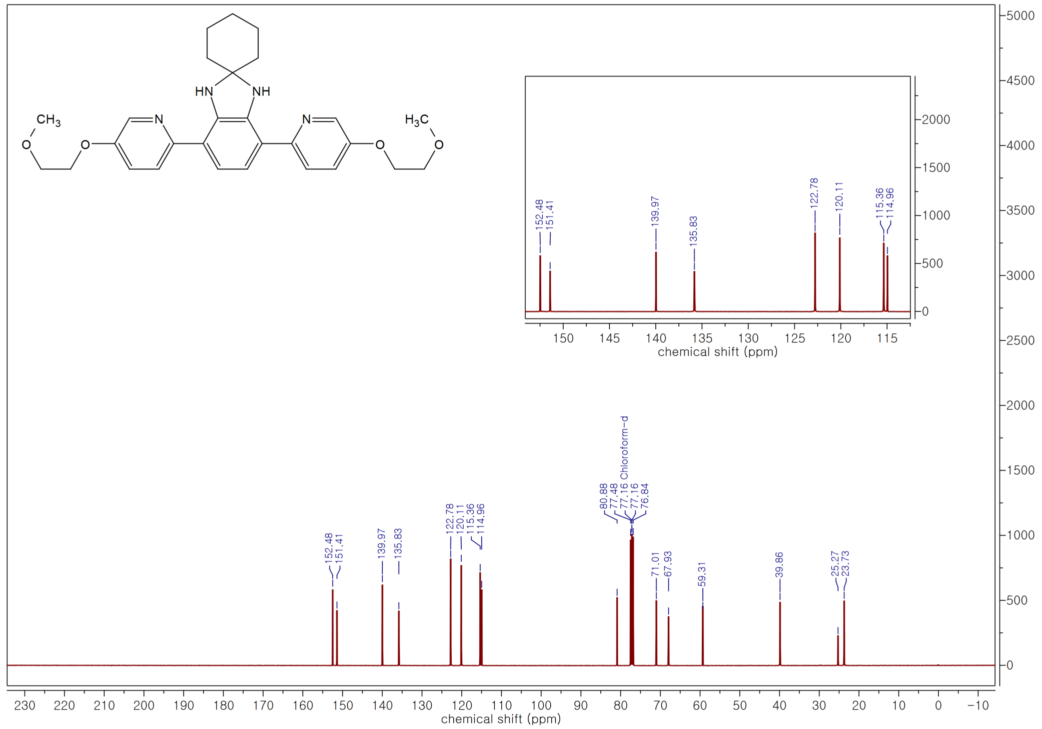

^{13}C NMR (100 MHz) spectrum of **8** in $CDCl_3$ (T = 298 K).

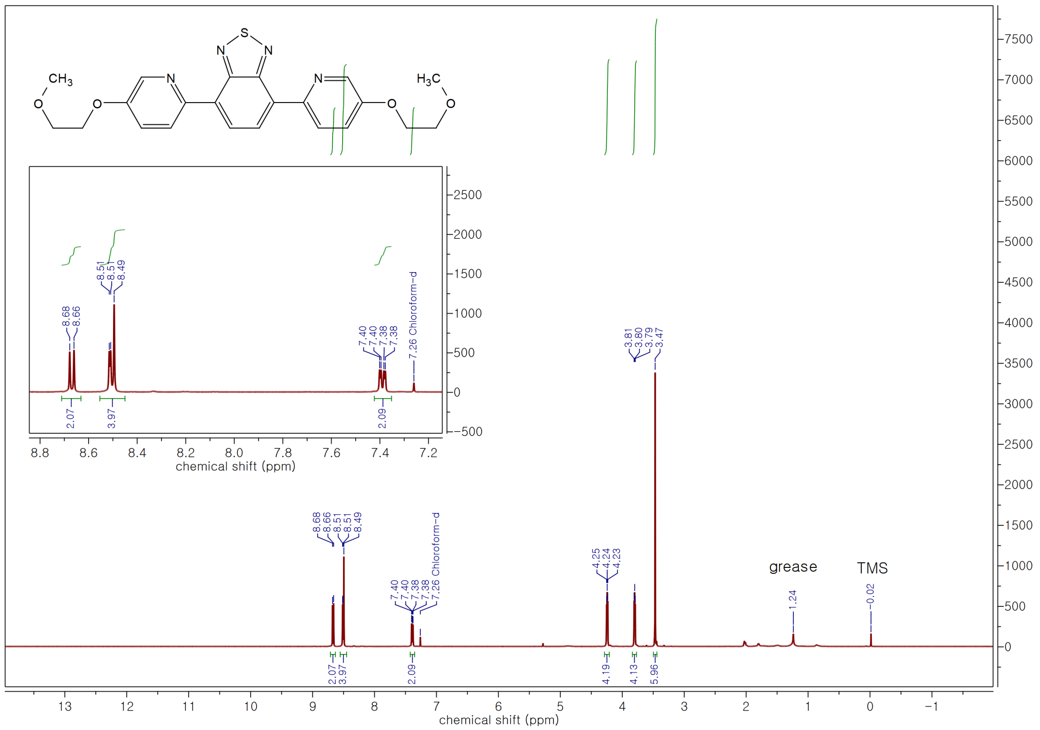

^{1}H NMR (500 MHz) spectrum of **9** in $CDCl_3$ (T = 298 K).

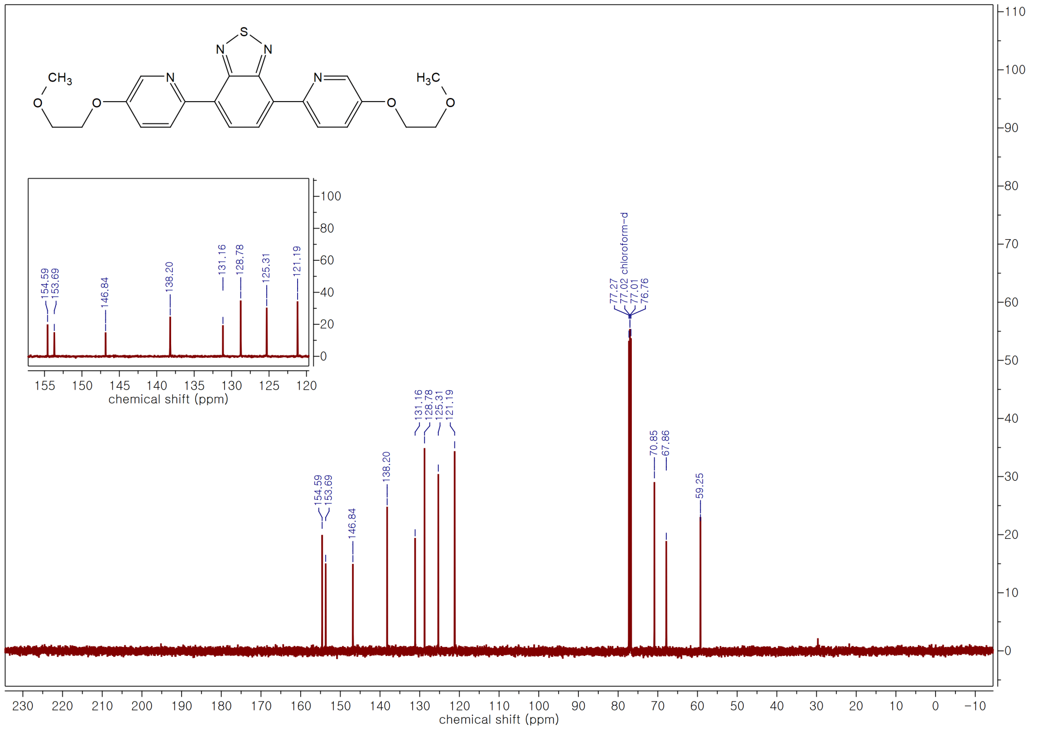

^{13}C NMR (125 MHz) spectrum of **9** in $CDCl_3$ (T = 298 K).
