# Supplementary material for: Molecular Tetris by sequence-specific stacking of hydrogen bonding molecular clips
Source: Commun Chem. 2022 Dec 28;5:180. doi: 10.1038/s42004-022-00802-4 (PMC9814962; doi:10.1038/s42004-022-00802-4)
Supplement: Supplementary file 5 — Supplementary Data 2 [file 42004_2022_802_MOESM5_ESM.pdf]

Supplementary Data 2

Molecular Tetris by sequence-specific stacking of hydrogen bonding molecular clips

Hyun Lee and Dongwhan Lee*

Department of Chemistry, Seoul National University, 1 Gwanak-ro, Gwanak-gu, Seoul 08826, Korea

DFT Cartesian coordinates

Cartesian coordinates of computed DFT model of **C-NI**,
E(B3LYP) = -2555.80131406 Hartree.

O	5.63315	-3.02783	0.42862	C	0.80858	5.84996	1.62261
O	8.24613	-4.02826	1.05240	H	0.96949	6.91536	1.75441
O	-10.91869	-1.70687	-1.14772	C	-8.98178	-0.33184	-1.06504
O	2.93076	1.65859	0.91553	H	-8.59834	0.66153	-1.33917
O	-8.09031	-1.34804	-1.51836	H	-9.09501	-0.37803	0.02617
O	1.21908	4.53942	-2.22776	C	3.73950	-2.13354	1.71388
N	-4.73576	-2.55346	-1.11686	H	4.34980	-1.62007	2.44711
N	-2.46069	-0.12519	1.83824	C	3.10307	2.43146	-5.37107
N	2.11580	-3.42077	-0.15935	H	2.99194	2.74695	-6.40358
N	0.05834	1.80961	1.22391	C	3.85707	1.31584	-5.06684
H	0.69199	1.07860	0.93510	H	4.34081	0.74780	-5.85689
N	2.05867	3.10910	-0.63370	C	3.43187	-3.53552	-0.21085
N	-0.22663	-0.70013	2.43413	H	3.85887	-4.15056	-1.00074
C	-1.06631	2.79270	2.93330	C	-10.32569	-0.56233	-1.72145
C	0.42884	3.13833	1.22081	H	-10.18290	-0.68558	-2.80773
C	-0.28384	3.80372	2.25836	H	-10.95500	0.33193	-1.56612
C	-0.64046	-1.46657	1.47310	C	6.55916	-2.37134	1.29447
C	2.84160	1.99719	-0.25882	H	6.44580	-2.73666	2.32322
C	-2.06307	-1.13401	1.12998	H	6.38712	-1.28559	1.28268
C	-2.78937	-1.91690	0.13843	C	7.95681	-2.67152	0.79952
C	-0.80147	1.61555	2.29463	H	8.01945	-2.44138	-0.27755
C	3.50950	1.24205	-1.34343	H	8.66711	-2.00735	1.32294
C	-1.31317	0.24827	2.67913	C	9.51619	-4.41752	0.56918
C	3.37085	1.64306	-2.69324	H	9.59348	-4.29631	-0.52301
C	1.34384	3.81258	0.40703	H	9.64592	-5.47333	0.81691
C	0.09235	-2.46927	0.71568	H	10.32923	-3.83960	1.03784
C	1.55722	-2.64649	0.80197	C	-12.14969	-2.04784	-1.75235
C	-0.09692	5.18037	2.44108	H	-12.03009	-2.27986	-2.82251
H	-0.64178	5.71299	3.21505	H	-12.89295	-1.23943	-1.65673
C	-1.74964	0.31899	4.16213	H	-12.52663	-2.93531	-1.23907
H	-2.26398	-0.61191	4.41863	C	4.25297	0.12072	-1.02248
H	-0.84022	0.38368	4.76828	H	4.32241	-0.16909	0.01811
C	-6.82667	-1.36518	-1.02317	C	-0.66155	-3.15541	-0.20345
C	1.89925	3.56127	-1.97179	H	-0.15942	-3.90287	-0.80787
C	-1.93532	2.87896	4.15281	C	-4.96109	-0.62431	0.29244
H	-2.67262	3.68663	4.06081	H	-4.51120	0.05941	1.00049
H	-1.31282	3.13604	5.02255	C	2.35103	-2.01050	1.76824
C	-2.65832	1.53678	4.40267	H	1.88533	-1.40097	2.53111
H	-3.04145	1.50739	5.42836	C	4.76388	-0.25935	-3.35513
H	-3.52040	1.45695	3.73219	H	5.24389	-0.84180	-4.13698
C	-4.20114	-1.68211	-0.22761	C	4.01135	0.88819	-3.72247
C	-5.99310	-2.39920	-1.49418	C	1.52893	5.17474	0.62289
H	-6.40248	-3.11228	-2.20713	H	2.22357	5.71602	-0.00815
C	-6.28727	-0.46100	-0.10757	C	4.87893	-0.63795	-2.03256
H	-6.87139	0.35863	0.29384	H	5.43887	-1.52536	-1.75460
C	2.46691	3.16683	-4.34932	C	-2.05473	-2.91250	-0.45645
H	1.86534	4.03999	-4.57717	H	-2.55219	-3.52783	-1.19771
C	4.30331	-2.88745	0.68481	C	2.59612	2.78063	-3.02649

Cartesian coordinates of computed DFT model of **C-P1**,
E(B3LYP) = -2504.84865388 Hartree.

O	7.03794	-2.17814	0.67432	C	-0.68315	3.60146	4.17344
O	-7.01225	-1.36928	1.28966	H	-0.65101	3.96827	5.20530
O	9.66896	-2.80252	1.65035	H	-1.68732	3.19878	4.00166
N	3.48722	-2.66729	0.80902	C	-0.26924	1.65976	-2.67587
N	-0.99153	1.02911	2.44968	C	-0.42716	4.76508	3.19127
N	1.38672	0.95568	2.30763	H	-1.28291	5.45118	3.19556
N	0.32435	2.69047	0.24049	H	0.43752	5.35456	3.53101
H	0.56482	1.80461	-0.17250	C	-0.61185	6.13363	0.05087
N	-3.48561	-2.30973	0.93955	H	-0.84449	6.92884	0.75329
O	-9.79886	-3.29376	0.12370	C	-1.27296	-1.00646	-2.34489
C	-0.61462	-0.12359	1.99377	C	-7.66019	-2.47548	0.66464
C	-2.89857	-1.24969	1.53005	H	-7.42764	-3.41377	1.18473
C	3.03047	-1.47609	1.26515	H	-7.34918	-2.57338	-0.38351
C	-1.42086	-1.25610	1.56288	C	-1.38678	1.37118	-1.81155
C	0.17807	2.92643	1.58702	H	-1.85623	2.19022	-1.27984
C	0.88507	-0.17263	1.90923	C	-0.03338	4.04939	-1.83954
C	1.56461	-1.36056	1.41037	C	0.34769	-1.82537	-4.01660
C	5.74480	-1.83963	0.90940	C	0.26520	0.58578	-3.45588
C	4.78687	-2.83779	0.64074	C	-9.15280	-2.19929	0.73790
H	5.13115	-3.80323	0.27504	H	-9.45709	-2.08548	1.79129
C	0.24296	1.81705	2.60789	H	-9.37952	-1.25121	0.22333
C	5.28708	-0.60947	1.38235	C	1.84114	-0.26854	-5.15208
H	5.96846	0.20175	1.60981	H	2.62992	-0.06563	-5.87173
C	-3.67241	-0.19467	2.05537	C	-0.64140	6.36744	-1.32055
H	-3.17948	0.65446	2.51019	H	-0.90045	7.35517	-1.69034
C	0.73029	-2.38682	1.04333	C	-0.22413	-0.74489	-3.27678
H	1.18699	-3.28860	0.65177	C	0.31265	2.95291	-2.77991
C	3.91549	-0.42946	1.56180	C	1.30787	0.83270	-4.39990
H	3.52713	0.51253	1.92670	C	-0.35887	5.34605	-2.24823
C	-5.05410	-0.25494	1.96393	H	-0.41780	5.55649	-3.31217
H	-5.68405	0.53853	2.35257	C	1.31189	3.16992	-3.74047
C	-0.70247	-2.33670	1.11718	H	1.74742	4.16165	-3.81694
H	-1.25485	-3.20184	0.77037	C	1.78866	2.14300	-4.54602
C	-0.18307	4.22886	1.81085	H	2.57448	2.34200	-5.26991
C	8.05093	-1.21158	0.94356	C	10.87257	-3.50611	1.41894
H	8.01605	-0.90055	1.99605	H	10.99762	-4.21177	2.24328
H	7.91063	-0.32541	0.30796	H	11.74453	-2.83226	1.39259
C	-0.28256	4.84849	0.51140	H	10.84328	-4.06744	0.47161
C	-5.65464	-1.36154	1.35501	C	-0.11982	-3.12987	-3.78939
C	9.39272	-1.84678	0.64989	H	0.32160	-3.95254	-4.34570
H	9.36443	-2.30917	-0.35063	C	-1.70877	-2.32697	-2.14698
H	10.16369	-1.05611	0.63132	H	-2.49009	-2.52123	-1.41784
C	-4.81421	-2.36927	0.85577	C	1.38836	-1.53851	-4.96464
H	-5.22108	-3.24894	0.36594	H	1.81161	-2.36337	-5.53174
C	0.01812	3.83987	-0.44946	C	-11.20746	-3.16697	0.11646
C	0.33311	2.45826	4.01093	H	-11.61766	-3.10839	1.13700
H	0.17022	1.67658	4.75891	H	-11.60937	-4.05544	-0.37576
H	1.35559	2.83129	4.13121	H	-11.53438	-2.27337	-0.43826
C	-1.85666	0.10367	-1.64777	C	-1.12984	-3.37496	-2.86068
H	-2.69322	-0.08483	-0.98248	H	-1.47154	-4.39236	-2.69334

Cartesian coordinates of computed DFT model of **C-P2**,
E(B3LYP) = -2504.84050320 Hartree.

O	-5.73938	4.03219	0.83068	H	4.31512	0.75129	-1.16221
O	7.88059	0.45994	1.56831	C	-3.14523	-4.06309	2.35317
N	-2.29579	3.40452	1.61385	C	2.40658	1.49775	-4.82594
N	-0.05814	1.88780	-1.91783	H	3.30708	1.24258	-4.25722
O	-7.91477	5.87654	0.46794	H	2.70900	2.18172	-5.62642
N	0.11521	-0.99011	-2.37270	C	1.42593	2.21677	-3.88713
H	-0.38135	-0.78737	-1.51959	H	0.48567	2.43515	-4.40369
N	2.23595	1.26801	-1.74865	H	1.84138	3.16239	-3.52672
N	4.45410	1.66219	2.07877	C	-1.22832	-2.93371	3.41205
O	10.61438	1.22487	2.01221	H	-0.74842	-2.56417	4.31435
C	0.36418	2.08479	-0.70694	C	-2.50848	-3.57694	3.53548
C	-4.45803	3.65694	0.58746	C	6.06391	0.58384	0.08309
C	-3.57242	3.74008	1.68049	H	6.70953	0.16234	-0.68039
H	-3.95603	4.09877	2.63365	C	-8.00689	4.48219	0.27284
C	-0.35919	2.58869	0.45153	H	-8.79096	4.23466	-0.46463
C	1.81190	1.69636	-0.60191	H	-8.25508	3.96058	1.21194
C	3.94840	1.45544	0.84773	C	-0.60948	-4.36678	-3.74163
C	-1.79037	2.95628	0.43931	H	-1.05562	-5.32975	-3.51136
C	2.52911	1.82663	0.65890	C	1.79878	0.21487	-5.42941
C	0.39220	2.68456	1.59671	H	1.09724	0.47825	-6.23500
H	-0.10185	3.05277	2.48899	H	2.58616	-0.38793	-5.89866
C	-3.94893	3.19958	-0.62834	C	8.45545	0.69284	2.85290
H	-4.57331	3.11231	-1.50962	H	7.93381	0.10078	3.61840
C	1.77781	2.32160	1.69557	H	8.38613	1.75551	3.11975
H	2.26605	2.43751	2.65683	C	-4.41619	-4.70827	2.44099
C	-0.00876	-2.15419	-3.10478	C	-4.42444	-5.03162	0.02059
C	-0.64279	-3.11089	-0.26339	H	-4.90336	-5.39738	-0.88378
H	0.34133	-2.65464	-0.32277	C	0.60582	-2.96244	-5.32042
C	1.08025	1.33988	-2.65438	H	1.07228	-2.81789	-6.29061
C	-2.60098	2.84771	-0.70003	C	-0.00218	-4.16971	-4.99533
H	-2.17441	2.49041	-1.62831	H	-0.00526	-4.98267	-5.71531
C	0.60067	-1.92768	-4.37208	C	-5.02918	-5.18496	1.23069
C	-1.24346	-3.25955	0.99413	H	-5.99637	-5.67558	1.30243
C	-2.51288	-3.90408	1.08664	C	9.91084	0.28258	2.79199
C	5.72300	1.34941	2.33165	H	10.31102	0.23940	3.82051
H	6.06719	1.54236	3.34369	H	9.98509	-0.72998	2.36193
C	-1.26515	-3.56730	-1.43685	C	-3.15214	-3.74388	4.77175
C	6.58239	0.80232	1.36363	H	-2.67088	-3.37403	5.67313
C	0.75125	-0.04358	-3.15092	C	-5.01994	-4.85288	3.69995
C	-6.67702	3.97580	-0.24201	H	-5.98682	-5.34402	3.77020
H	-6.78478	2.94171	-0.59970	C	-9.09027	6.43121	1.02271
H	-6.34435	4.60593	-1.07746	H	-9.96660	6.27194	0.37325
C	1.08570	-0.57117	-4.36900	H	-8.92142	7.50511	1.12925
C	-0.62519	-3.37067	-2.75958	H	-9.31537	6.00652	2.01385
C	-0.62354	-2.78388	2.20167	C	-4.39282	-4.37486	4.84964
H	0.34334	-2.29330	2.12668	H	-4.87529	-4.49524	5.81514
C	-3.14425	-4.38682	-0.09907	C	11.97478	0.88473	1.83704
C	-2.51027	-4.20439	-1.33548	H	12.09246	-0.07892	1.31647
H	-3.00707	-4.54275	-2.24034	H	12.51081	0.82384	2.79832
C	4.74305	0.90974	-0.18104	H	12.42875	1.67124	1.23000

Cartesian coordinates of computed DFT model of **C-P4**,
E(B3LYP) = -2504.83766604 Hartree.

O	-7.86168	-0.87824	-1.84775	H	-10.75468	0.66781	-1.29920
O	5.56153	-4.58764	0.09154	H	-9.93812	0.31417	-2.84209
N	0.02038	-1.46511	2.08530	C	-0.45349	4.17555	3.98808
N	-4.53267	-2.21503	-1.80645	H	-0.90420	4.32586	4.96486
N	-0.05063	1.44011	1.71617	C	-1.72095	1.18645	4.99833
H	0.37572	1.01089	0.91044	H	-2.47514	1.92736	5.29147
N	-2.25033	-0.82705	1.74861	H	-1.01495	1.12270	5.83981
O	-10.70793	-1.31208	-1.94868	C	7.84910	-4.85253	0.68504
N	2.13748	-4.06172	-0.83902	H	8.54618	-4.76711	1.53754
O	8.35606	-4.19259	-0.45413	H	7.69017	-5.92604	0.49003
C	1.67458	-3.24767	0.14014	C	0.75395	5.04613	2.05490
C	-0.42516	-1.98477	0.98326	H	1.21625	5.89133	1.55447
C	-3.99767	-1.67357	-0.68546	C	1.33261	3.57397	0.06037
C	-0.70381	0.76750	2.72828	C	-2.38863	-0.18534	4.77312
C	-2.59553	-2.03995	-0.39683	H	-2.70638	-0.61014	5.73165
C	-5.78112	-1.92555	-2.13063	H	-3.28617	-0.05841	4.15829
H	-6.19053	-2.37370	-3.03389	C	0.16157	5.22798	3.31922
C	-6.60487	-1.07157	-1.37072	H	0.18556	6.21406	3.77350
C	2.51127	-2.82770	1.18447	C	2.72602	3.87396	-0.23033
H	2.11869	-2.17988	1.95745	C	0.53928	3.04005	-0.92202
C	-1.85753	-1.58288	0.77237	H	-0.50280	2.82363	-0.70106
C	3.84081	-3.24836	1.21991	C	0.18853	2.22870	-3.24265
H	4.48554	-2.92014	2.02658	H	-0.84277	1.98620	-3.00100
C	4.30572	-4.08555	0.20527	C	2.00397	2.30318	-4.84177
C	0.26062	-2.83508	0.02103	H	2.38204	2.11699	-5.84337
C	-1.08730	-0.68616	2.63720	C	9.55334	-4.76951	-0.93456
C	0.11460	2.75567	2.09359	H	9.86401	-4.18939	-1.80639
C	3.39705	-4.46071	-0.80440	H	9.40954	-5.81856	-1.23825
H	3.74707	-5.11313	-1.60200	H	10.35721	-4.73673	-0.18100
C	-0.50999	-3.22800	-1.04522	C	-11.19333	-1.85683	-0.73635
H	-0.04453	-3.86233	-1.79138	H	-11.85903	-1.15432	-0.20901
C	-1.00055	1.62299	3.75683	H	-10.38657	-2.15212	-0.04954
C	-1.88017	-2.84782	-1.24532	H	-11.76302	-2.75132	-0.99826
H	-2.38493	-3.21506	-2.13193	C	3.21895	3.63895	-1.55429
C	-0.48022	2.91132	3.37831	C	2.36839	3.09366	-2.56118
C	-6.06507	-0.50590	-0.21525	C	5.05203	3.69028	-3.20814
H	-6.64106	0.16358	0.41279	H	6.08820	3.92469	-3.43746
C	-4.74836	-0.81275	0.12861	C	1.01404	2.77728	-2.24749
H	-4.29883	-0.39112	1.01824	C	5.42523	4.43821	-0.87042
C	-8.75439	-0.06201	-1.09668	H	6.46068	4.66195	-1.11293
H	-8.37317	0.96769	-1.02489	C	4.58196	3.93308	-1.87149
H	-8.85710	-0.45289	-0.07507	C	4.23483	3.17539	-4.16643
C	6.53113	-4.21506	1.06824	H	4.60858	2.99410	-5.17064
H	6.22340	-4.56550	2.06403	C	3.61993	4.35614	0.74119
H	6.64723	-3.12362	1.09800	H	3.27066	4.50465	1.75582
C	-1.44973	-1.17371	4.06563	C	2.86525	2.85198	-3.87819
H	-1.90518	-2.16472	3.98076	C	0.68210	1.99495	-4.52440
H	-0.51156	-1.27892	4.62003	H	0.03172	1.56793	-5.28200
C	0.74871	3.80582	1.40885	C	4.94738	4.63629	0.42136
C	-10.09440	-0.04720	-1.82121	H	5.61465	5.00972	1.19265

Cartesian coordinates of computed DFT model of PHD,
E(B3LYP) = -720.85701759 Hartree.

C	3.49675	-0.83441	-0.00009	C	-3.49674	-0.83441	0.00011
C	2.85869	0.39709	0.00015	C	-2.69904	-1.98526	0.00031
C	1.45895	0.43509	0.00015	H	4.57871	-0.91352	-0.00008
C	0.74560	-0.78356	0.00003	H	3.40349	1.33555	0.00029
C	2.69904	-1.98526	-0.00038	H	3.16201	-2.97042	-0.00056
C	0.77395	1.75178	0.00022	H	-4.57871	-0.91353	0.00013
C	-0.74560	-0.78356	0.00004	H	-3.16201	-2.97043	0.00050
C	-1.45895	0.43509	-0.00010	O	1.37592	2.80951	0.00061
C	-0.77396	1.75178	-0.00023	O	-1.37593	2.80951	-0.00067
C	-2.85869	0.39709	-0.00010	N	1.36484	-1.97314	-0.00026
H	-3.40349	1.33554	-0.00022	N	-1.36484	-1.97315	0.00023

Cartesian coordinates of computed DFT model of phen,
E(B3LYP) = -571.63879809 Hartree.

C	0.00000	1.41642	0.87050	C	0.00000	-3.48156	-0.37188
C	0.00000	2.70365	-1.54856	C	0.00000	-2.70365	-1.54856
C	0.00000	2.82836	0.84227	N	0.00000	-1.38067	-1.56448
C	0.00000	0.72925	-0.37880	H	0.00000	3.19525	-2.52058
N	0.00000	1.38067	-1.56448	H	0.00000	3.38101	1.77834
C	0.00000	3.48156	-0.37188	H	0.00000	4.56516	-0.43252
C	0.00000	-0.72925	-0.37880	H	0.00000	-1.23439	3.03638
C	0.00000	-1.41642	0.87050	H	0.00000	1.23439	3.03638
C	0.00000	-0.68022	2.10142	H	0.00000	-3.38101	1.77834
C	0.00000	0.68022	2.10142	H	0.00000	-4.56516	-0.43252
C	0.00000	-2.82836	0.84227	H	0.00000	-3.19525	-2.52058

Cartesian coordinates of computed DFT model of PHQ,
E(B3LYP) = -688.79265272 Hartree.

C	0.00002	-0.77286	1.74365	C	0.00019	-2.87900	-1.96548
C	0.00019	1.46493	0.43113	C	0.00030	-1.48640	-1.97856
C	0.00000	-0.74245	-0.78679	O	0.00032	-1.36673	2.80759
C	0.00000	0.74245	-0.78679	O	-0.00032	1.36673	2.80759
C	-0.00019	-1.46493	0.43113	H	-0.00074	0.98414	-2.93831
C	-0.00002	0.77286	1.74365	H	-0.00043	3.41927	-2.90757
C	-0.00030	1.48640	-1.97856	H	0.00034	4.66486	-0.74870
C	-0.00019	2.87900	-1.96548	H	0.00045	3.36825	1.39712
C	0.00020	3.57957	-0.75640	H	-0.00045	-3.36825	1.39712
C	0.00032	2.86662	0.43524	H	-0.00034	-4.66486	-0.74870
C	-0.00032	-2.86662	0.43524	H	0.00043	-3.41927	-2.90757
C	-0.00020	-3.57957	-0.75640	H	0.00074	-0.98414	-2.93831
